# Transition-metal-free decarbonylation–oxidation of 3-arylbenzofuran-2(3*H*)-ones: access to 2-hydroxybenzophenones

**DOI:** 10.3762/bjoc.20.223

**Published:** 2024-10-21

**Authors:** Bhaskar B Dhotare, Seema V Kanojia, Chahna K Sakhiya, Amey Wadawale, Dibakar Goswami

**Affiliations:** 1 Bio-Organic Division, Bhabha Atomic Research Centre, Trombay, Mumbai, PIN-400085, India; 2 NMIMS Sunandan Divatia School of Science, Vile-Parle, Mumbai-400056, India; 3 Chemistry Division, Bhabha Atomic Research Centre, Trombay, Mumbai-400085, India; 4 Homi Bhabha National Institute, Anushaktinagar, Mumbai, PIN-400094, India

**Keywords:** decarbonylation–oxidation, hydroperoxide, 2-hydroxybenzophenone, transition-metal-free, UV-protection

## Abstract

A transition-metal-free decarbonylation–oxidation protocol for the conversion of 3-arylbenzofuran-2(3*H*)-ones to 2-hydroxybenzophenones under mild conditions has been developed. NMR studies confirmed the role of in-situ-generated hydroperoxide in the conversion. The protocol was applied to a diverse range of substrates to access the target products in good to excellent yields. A structure–activity study for the 5-substituted-2-hydroxybenzophenones towards UV-protection abilities has been verified by mathematical calculations.

## Introduction

**Figure 1:**
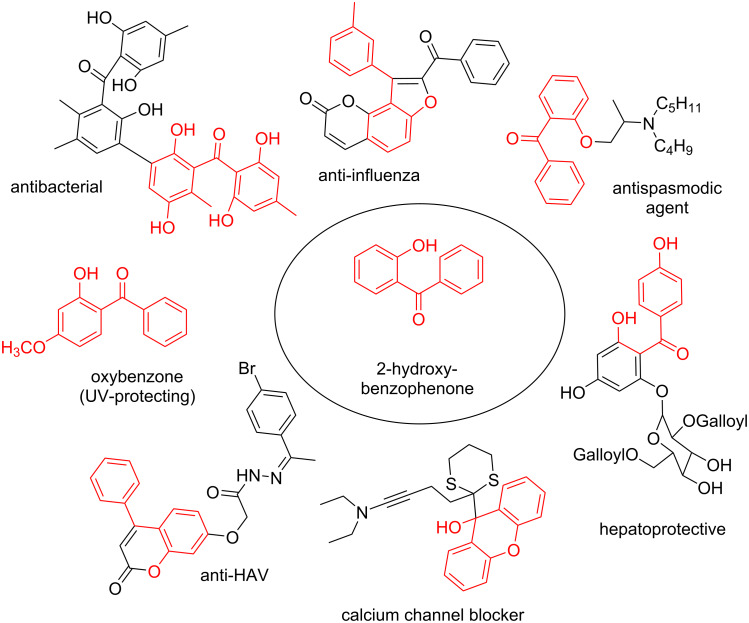
Some 2-hydroxybenzophenone derivatives with varied activities.

Benzophenone compounds are ubiquitous in nature, and show biological activities such as anti-inflammatory, antiviral, and anticancer effects [1]. Amongst these, 2-hydroxybenzophenones are regarded as one of the most important classes of compounds owing to their varied bioactivities, including calcium channel blockers, anti-influenza drugs, anti-HAV drugs, and antispasmodic agents (Figure 1) [2]. Along with that, oxybenzone, a 2-hydroxybenzophenone, has been widely used as a sun-protecting material in cosmetics [3]. Moreover, in general, 2-hydroxybenzophenones are regarded as an important ultraviolet absorber, as well as an important template in synthesis [4].

Mechanistically, it has been well accepted that 2-hydroxybenzophenones show UV-absorbing as well as photo-antioxidant properties via an intramolecular hydrogen transfer [5]. The structure–activity relationship between the substituents of 2-hydroxybenzophenones and their UV-absorption properties has been reported previously [6]. It was concluded that the ability to absorb UV light and the corresponding abilities to prevent photodegradation are substantially improved by a substituent in the 5-position rather than by the presence of a substituent in the 4-position. Moreover, either oxybenzone, which contains a methoxy group in the 5-position and is the most commonly used commercial UV-protector, or its metabolites, have been associated with estrogenic activities [7]. A further structure–activity relationship study revealed that a 5-substitution decreases estrogenic activity. Similar results were obtained in another study where 2-hydroxy-5-methylbenzophenone was found to exhibit very weak estrogenic activity [8]. Although a detailed SAR is still warranted, the initial reports prompted us to find a suitable synthetic method for 5-substituted 2-hydroxybenzopheneones, and to evaluate their UV-absorbing properties.

**Figure 2:**
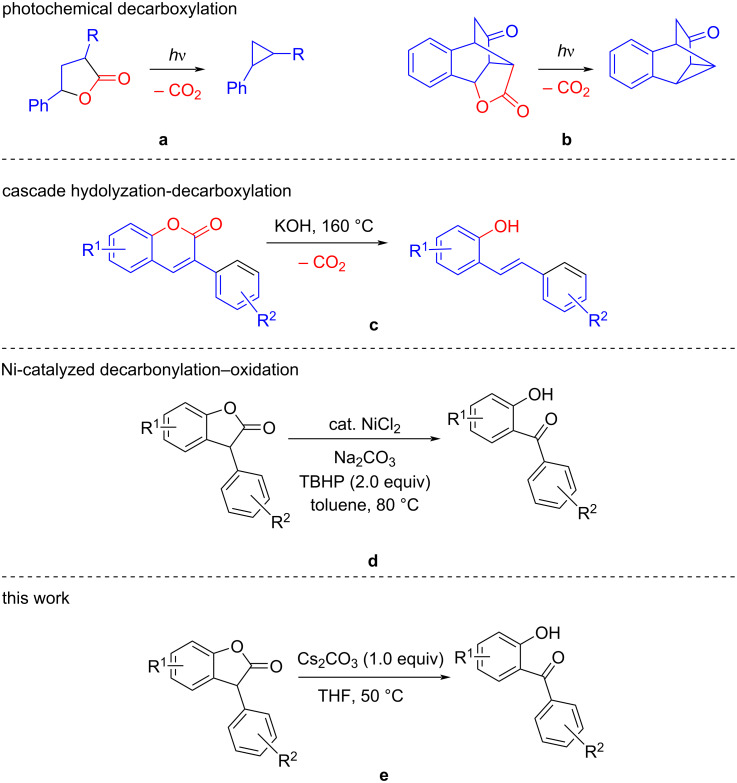
Decarbonylation–oxidation of lactones.

Various methods for the synthesis of benzophenones have been reported over the years (Figure 2) [2]. Conventionally, benzophenones are synthesized by Friedel–Crafts acylation of benzoyl halides and aromatic compounds. However, the regioselectivity of Friedel–Crafts benzoylation at the desired position is difficult to control [9]. On the other hand, 2-hydroxybenzophenones are conventionally prepared via Fries rearrangement of a phenyl ester [10]. Organocatalytic methods have also been reported for the synthesis of 2-hydroxybenzophenones [11]. In addition, several metal-mediated methods for their synthesis have been reported. For example, the Rh-catalyzed rearrangement of 2-aryloxybenzaldehydes yielded 2-hydroxybenzophenone [12]. Pd-catalyzed *o*-hydroxylation of benzophenones gave moderate yield of the title compound, and Br-substituted substrates were found to be not compatible with this method [13]. Various metals (Rh, Cu, Ir etc.) were applied to catalyze the oxidative coupling of salicylaldehyde with arylboronic acids to successfully produce 2-hydroxybenzophenones [14]. Recently, a Ni-catalyzed decarbonylation–oxidation of 3-arylbenzofuran-2(3*H*)-ones emerged as an innovative route to access 2-hydroxybenzophenones [2].

The use of transition metals poses environmental hazards, and this can only be circumvented using a transition-metal-free synthesis protocol. Although eco-friendly methods for the synthesis of 2-hydroxybenzophenones have been reported via a cycloaromatization of α-alkenoyl ketene dithioacetals and nitroethane in water [15], an alternate and more versatile method using decarbonylation of benzofuranone followed by oxidation, that produces only CO_2_ as a non-toxic side product, is considered less hazardous. Although decarboxylation of aldehydes, carboxylic acids, and ketones are well known, albeit using metal catalysts, decarboxylation methods for lactones are limited. Both photochemical decarboxylation methods reported for α,γ-butyrolactone [16] and γ-butyrolactones [17] yielded the products in very poor to moderate yields (Figure 2a and 2b). Recently, a transition-metal-free decarboxylation of α,β-unsaturated aromatic lactones was reported for the synthesis of *E*-*o*-hydroxystilbenes, albeit via a cascade hydrolyzation–decarboxylation reaction at a very high temperature (Figure 2c) [18]. However, a metal-free decarbonylation–oxidation method for benzofuranones is still unprecedented. The earlier report [2] on the Ni-catalyzed decarbonylation–oxidation protocol using a hydroperoxide (Figure 2d) gave us an idea that the use of an in-situ-generated hydroperoxide may trigger the reaction, and may avoid the use of transition metal in the reaction. Many solvents, e.g., tetrahydrofuran (THF), dioxanes etc. are known for producing hydroperoxides in situ on long standing. Also, it has been reported that the generation of the hydroperoxide may be accelerated by heating the solvent under open atmospheric conditions [19]. We envisaged that such autooxidation of THF [20] will produce THF hydroperoxide, and this will facilitate the transformation of 3-arylbenzofuran-2(3*H*)-ones to 2-hydroxybenzophenones via decarbonylation–oxidation quickly and without the need of a transition-metal catalyst. Herein, a novel decarbonylation–oxidation method for 3-arylbenzofuran-2(3*H*)-ones has been developed for the synthesis of 2-hydroxybenzophenones via a transition-metal-free synthetic route.

## Results and Discussion

**Scheme 1:**
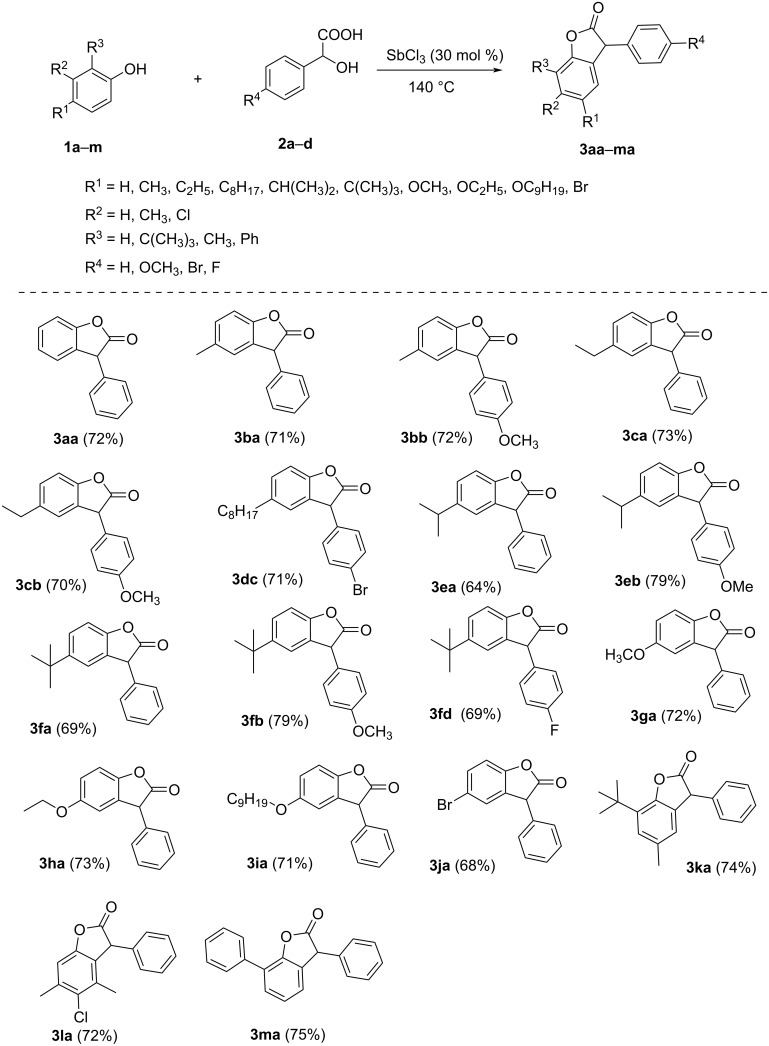
Synthesis of 3-arylbenzofuran-2(3*H*)-ones.

Initially, 3-arylbenzofuran-2(3*H*)-ones **3aa**–**ma** were prepared following a SbCl_3_-catalyzed Friedel–Crafts alkylation of phenols **1a**–**m** with benzylic alcohols **2a**–**d**, earlier reported by us (Scheme 1) [21–23]. All the synthesized 3-arylbenzofuran-2(3*H*)-ones were characterized using ^1^H NMR, ^13^C NMR, FTIR spectroscopy, and elemental analysis.

Next, in a model experiment, we carried out the decarbonylation–oxidation reaction of 5-methyl-3-phenylbenzofuran-2(3*H*)-one (**3ba**) using different bases in different solvents (Table 1) under open atmospheric conditions. In the presence of Cs_2_CO_3_ (2.0 equiv) in THF, the reaction gave 92% yield of product **4ba** (Table 1, entry 1). When the amount of Cs_2_CO_3_ was decreased to 1 equiv, the yield of the reaction did not decrease appreciably (Table 1, entry 2). However, further decreasing the amount of Cs_2_CO_3_ decreased the reaction yield drastically (Table 1, entry 3). The yield of the product using K_2_CO_3_ in THF as a base was not high (46–54%, Table 1, entries 4–6). Other bases like KO*t*-Bu or KOH, or a mixture of both gave moderate to good yields (59–84%, Table 1, entries 7–9). Use of BuLi as a base decreased the yield drastically (Table 1, entry 10). Other bases like NaOMe, triethylamine, DBU, DMAP, sodium acetate, or DABCO also produced the product in moderate to good yields (42–87%) when used in excess (Table 1, entries 11–16). Interestingly, the reaction did not proceed in the presence of pyridine as a base (Table 1, entry 17). Finally, after getting the best yield of the product using Cs_2_CO_3_, we changed the solvent to dichloromethane, 1,4-dioxane, and acetonitrile. Except for 1,4-dioxane (Table 1, entries 18–20), where hydroperoxide is known to be produced in situ, the yields were very low. However, the reaction in 1,4-dioxane took a long time to complete. Hence, it was established that only those solvents which can produce hydroperoxides in situ were suitable for the reaction. Therefore, we chose Cs_2_CO_3_ as the base of choice, and performed the further reactions using 1.0 equiv of Cs_2_CO_3_ in THF at 50 °C in an open atmosphere. However, it must be noted that the yields recorded in Table 1 are isolated yields. For the sake of eliminating errors in isolation, we have carried out every reaction in triplicate, and have considered the average yield as the isolated yield.

**Table 1:** Optimization of reaction conditions.^a^

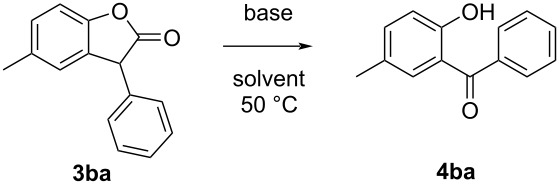				

entry	base (equiv)	solvent (2 mL)	time (h)	yield^b^ (%)

1	Cs_2_CO_3_ (2.0)	THF	4	92
2	Cs_2_CO_3_ (1.0)	THF	4	91
3	Cs_2_CO_3_ (0.5)	THF	4	61
4	K_2_CO_3_ (2.0)	THF	6	54
5	K_2_CO_3_ (1.0)	THF	6	51
6	K_2_CO_3_ (0.5)	THF	6	46
7	KO*t*-Bu (2.0)	THF	6	84
8	KOH (2.0)	THF	6	79
9	KOH (1.0)^c^	THF	6	59
10	BuLi (2.0)	THF	6	22
11	NaOMe (2.0)	THF	6	81
12	Et_3_N (2.0)	THF	6	68
13	DBU (2.0)	THF	9	79
14	DMAP (2.0)	THF	9	46
15	CH_3_COONa (2.0)	THF	9	87
16	DABCO (2.0)	THF	9	42
17	pyridine (2.0)	THF	NR^d^	–
18	Cs_2_CO_3_ (1.0)	DCM	6	11
19	Cs_2_CO_3_ (1.0)	1,4-dioxane	8	82
20	Cs_2_CO_3_ (1.0)	CH_3_CN	14	25

^a^The reactions were carried out in 0.5 mmol scale. ^b^Isolated yields of the products. ^c^0.5 equiv KO*t*-Bu was added. ^d^NR = no reaction.

Earlier, Qui et al. reported [2] that a transition-metal catalyst was essential for this reaction to happen at a higher temperature, and the products were obtained in negligible yields without the catalyst. Our protocol established that the reaction proceeds without the need for a transition-metal catalyst, as well as at a lower temperature. Additionally, the use of a hydroperoxide generating solvent made the protocol operationally simple.

**Scheme 2:**
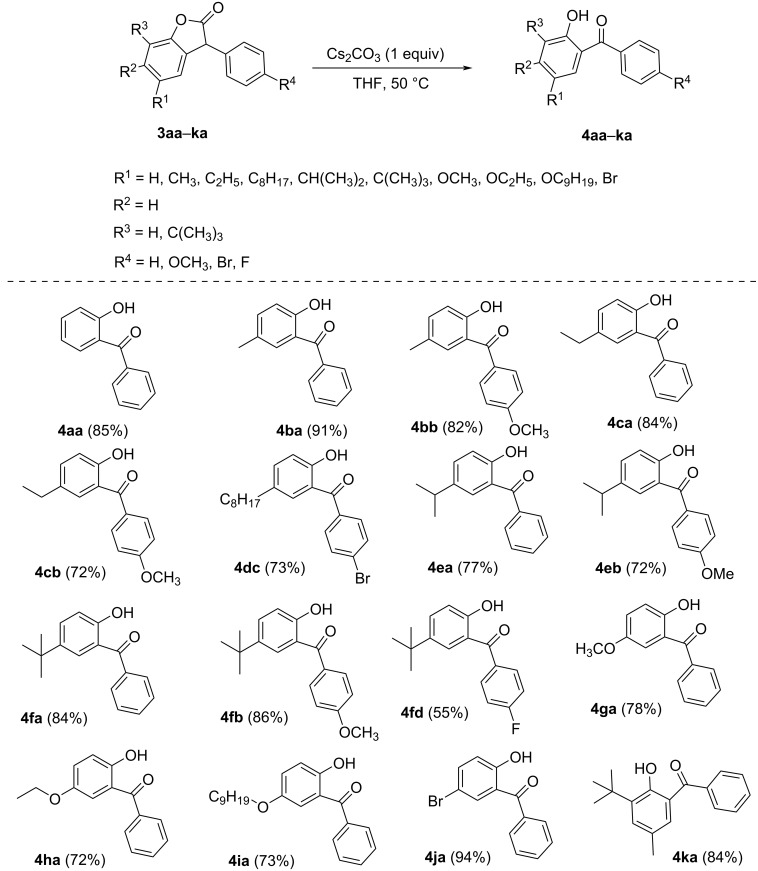
Synthesis of 2-hydroxybenzophenones.

To investigate the generality of the protocol, the previously synthesized 3-arylbenzofuran-2(3*H*)-ones **3aa**–**ka** were treated with 1.0 equiv Cs_2_CO_3_ in THF under open atmospheric heating. As revealed in Scheme 2, the reaction of 3-arylbenzofuran-2(3*H*)-ones with both electron-donating as well as electron-withdrawing substituents reacted smoothly to afford the corresponding benzophenones **4aa–ka** in good to high yields. Substitutions at the 5, 6, or 7 positions of the benzofuranones did not hamper the reaction, although a substitution at the 4-position of benzofuranone hindered the reaction, leading to a very poor yield of the desired benzophenone product.

**Figure 3:**
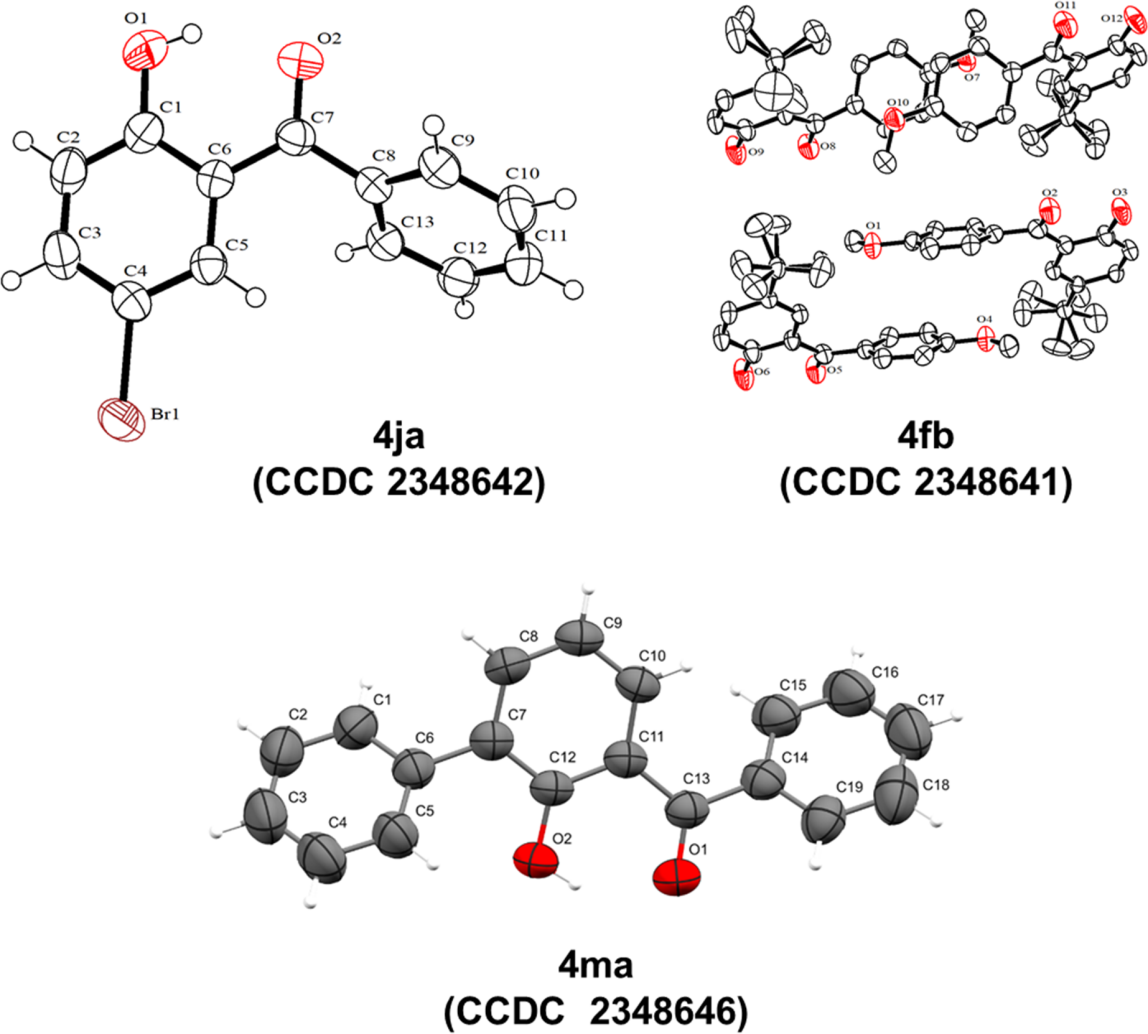
The ORTEP view of the compounds **4ja**, **4fb**, and **4ma**.

Further, to confirm the structure and the substitution pattern in the 2-hydroxybenzophenones, single crystal XRD data were collected for the representative compounds **4ja**, **4fb**, and **4ma**. Whereas compound **4ja** and **4ma** showed an ideal single crystal behavior, compound **4fb** showed a dynamic disordered structure due to intramolecular vibrations in the unit cells, particularly because of the presence of the *tert*-butyl group, which can assume any rotation angle [24]. Nevertheless, all the structures corroborated with the expected structure, as shown in Figure 3.

To confirm the formation of hydroperoxide in THF via autooxidation, freshly distilled THF was heated under open atmosphere at 50 °C for 4 h, and was concentrated under vacuum to obtain the hydroperoxide residue. ^1^H NMR analysis of the residue, recorded in CDCl_3_ (Supporting Information File 1, Figure S1) confirmed the presence of hydroperoxide. Resonances attributable to O–CH–O were observed as a quartet centered at δ 5.40 ppm in the NMR spectrum. The –O–OH group in hydroperoxide gave rise to a singlet at 10.91 ppm. The NMR spectrum was consistent with the generation of tetrahydrofuran-2-hydroperoxide in THF, and was similar to the NMR reported earlier [25].

**Scheme 3:**
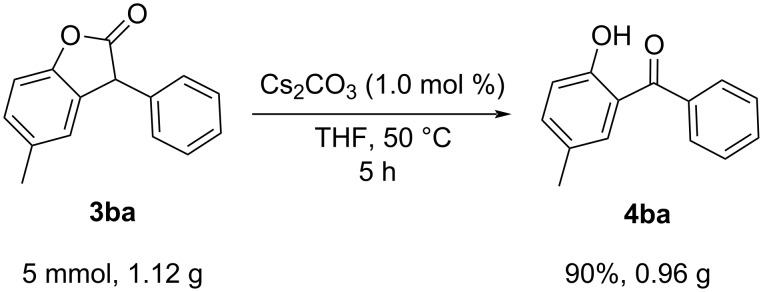
Gram-scale experiment.

Further, the reaction of **3ba** was scaled up to demonstrate the synthetic utility of our protocol. The reactions involving hydroperoxides are known to be difficult to scale-up due to their violent reactions at elevated temperatures. Our protocol involved mild conditions, and to our expectations, a gram-scale conversion of **3ba** to **4ba** proceeded smoothly without appreciable loss in the reaction yield (Scheme 3). The reduced reaction time and a lower reaction temperature proved to be advantageous compared to the reported Ni-catalyzed decarbonylation–oxidation method of benzofuranones [2].

**Scheme 4:**
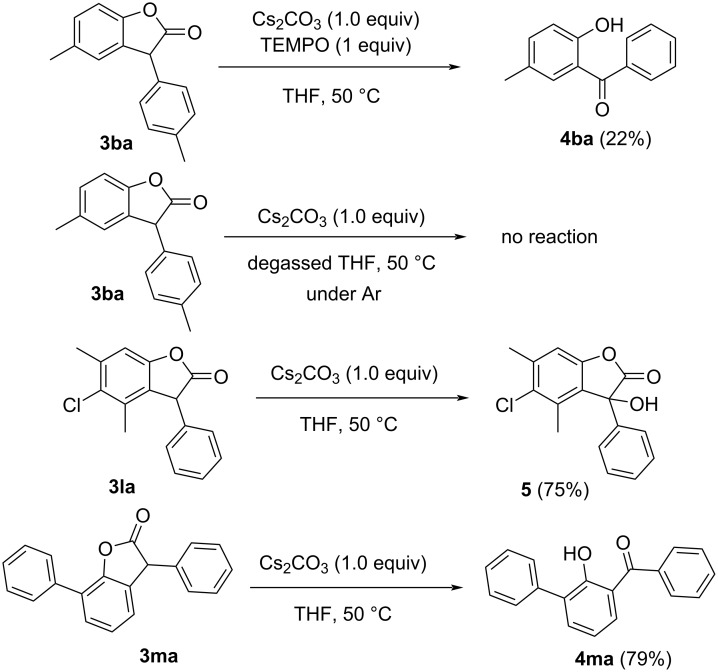
Control experiments.

Next, to elucidate the mechanism of the decarbonylation–oxidation reaction of 3-arylbenzofuran-2(3*H*)-ones **3aa**–**ka**, control experiments were performed. The fact that the reaction proceeded well only in solvents which can produce hydroperoxides in situ, we hypothesized that hydroperoxides have a pivotal role in the reaction mechanism. In order to confirm that the reaction proceeds through a radical mechanism, the decarbonylation–oxidation reaction of **3ba** was performed in the presence of TEMPO, a known radical quencher [26]. To our expectations, the reaction did not proceed well in presence of TEMPO, giving a very poor yield of **4ba**, confirming the role of radical in the reaction (Scheme 4). In addition, since it has been reported that the formation of hydroperoxides in THF is catalyzed by both dissolved oxygen and atmospheric oxygen [27], we envisaged that degassed THF solvent kept under inert atmosphere would not produce hydroperoxides, and hence the reaction will not proceed under such circumstances. To confirm this hypothesis, we degassed freshly distilled THF with Ar for 2 h, and then the reaction was performed under inert atmosphere. As expected, the reaction did not proceed under these conditions, confirming the role of hydroperoxide in the reaction. Additionally, as discussed earlier, we observed that the yields of the 2-hydroxybenzophenone product using our protocol was very poor when a 4-substituted benzofuranone was treated even for 24 h under these conditions. To follow this, we prepared 5-chloro-4,6-dimethyl-3-phenylbenzofuran-2(3*H*)-one (**3la**) and submitted it to similar conditions. To our surprise, 5-chloro-3-hydroxy-4,6-dimethyl-3-phenylbenzofuran-2(3*H*)-one (**5**) was obtained in the reaction. This showed that the bulky THF hydroperoxide could not react with **3la** due to steric reasons. Similar poor yields for 6-substituted-2-hydroxybenzophenones were previously reported in decarbonylation–oxidation reactions of benzofuranones [2]. To further confirm whether a bulky substitution at the 7-position of the benzofuranone also hinders the formation of 2-hydroxybenzophenone, we prepared 3,7-diphenylbenzofuran-2-one (**3ma**). However, on heating **3ma** with Cs_2_CO_3_ in THF, the corresponding benzophenone **4ma** was obtained, which confirmed that a bulky substituent in the 7-position does not hinder the reaction.

**Figure 4:**
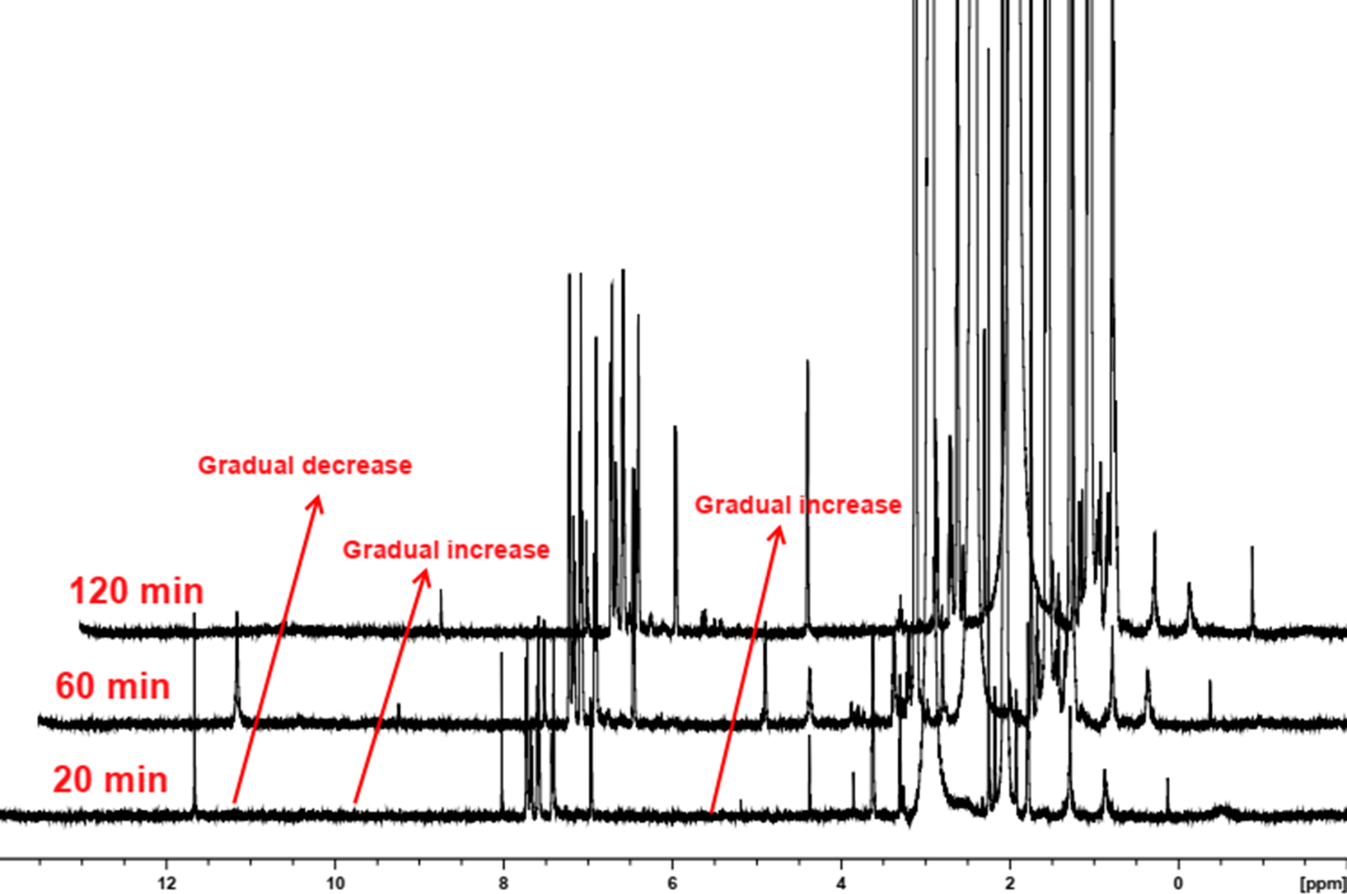
Partial ^1^H NMR spectra of the aliquots (taken at different time intervals) from the reaction mixture.

Further, to understand the origin of the product, we monitored the progress of the reaction of **3aa** using ^1^H NMR spectroscopy. Aliquots from the reaction mixture were taken at different time intervals (Figure 4), THF evaporated under vacuum, and the residue was extracted with CHCl_3_ and the ^1^H NMR spectra were recorded in CDCl_3_.

**Figure 5:**
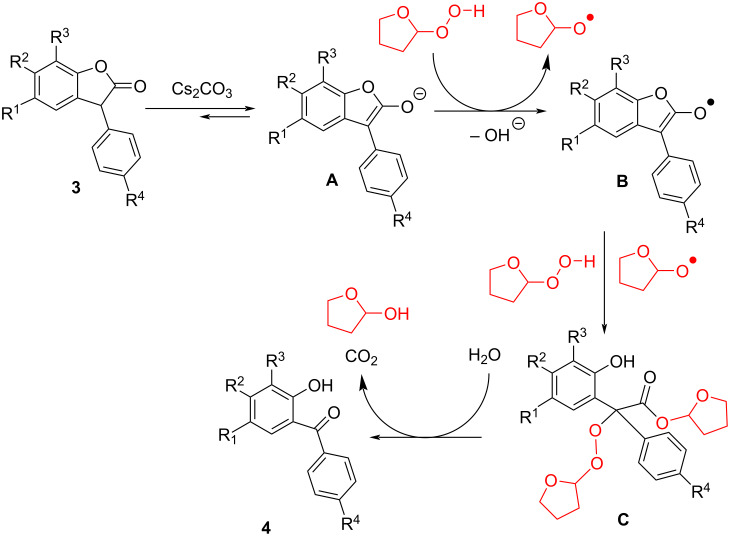
Plausible mechanism for the transition-metal-free decarbonylation–oxidation.

As can be seen from Figure 4, a gradual increase of the peak at δ 5.3 ppm with concomitant decrease of the peak at δ 11.6 ppm is observed and indicated the gradual formation of THF-hydroperoxide, along with the concomitant insertion of the hydroperoxide into the substrate leading to the decrease in the –O–OH peak. An increase in the peak at δ 9.6 ppm indicated the formation of a phenolic moiety over time. Based on these observations, a plausible reaction mechanism is proposed (Figure 5). Proton abstraction followed by enolization of benzofuranone **3** in the presence of a base produced intermediate **A**. The latter reacted with hydroperoxide to form **B** with the concomitant generation of the radicals, which further reacted with intermediate **B** to form intermediate **C**. Finally, **C** is hydrolysed with the release of one molecule CO_2_ and two molecules 2-hydroxytetrahydrofuran to give the target product **4**.

**Figure 6:**
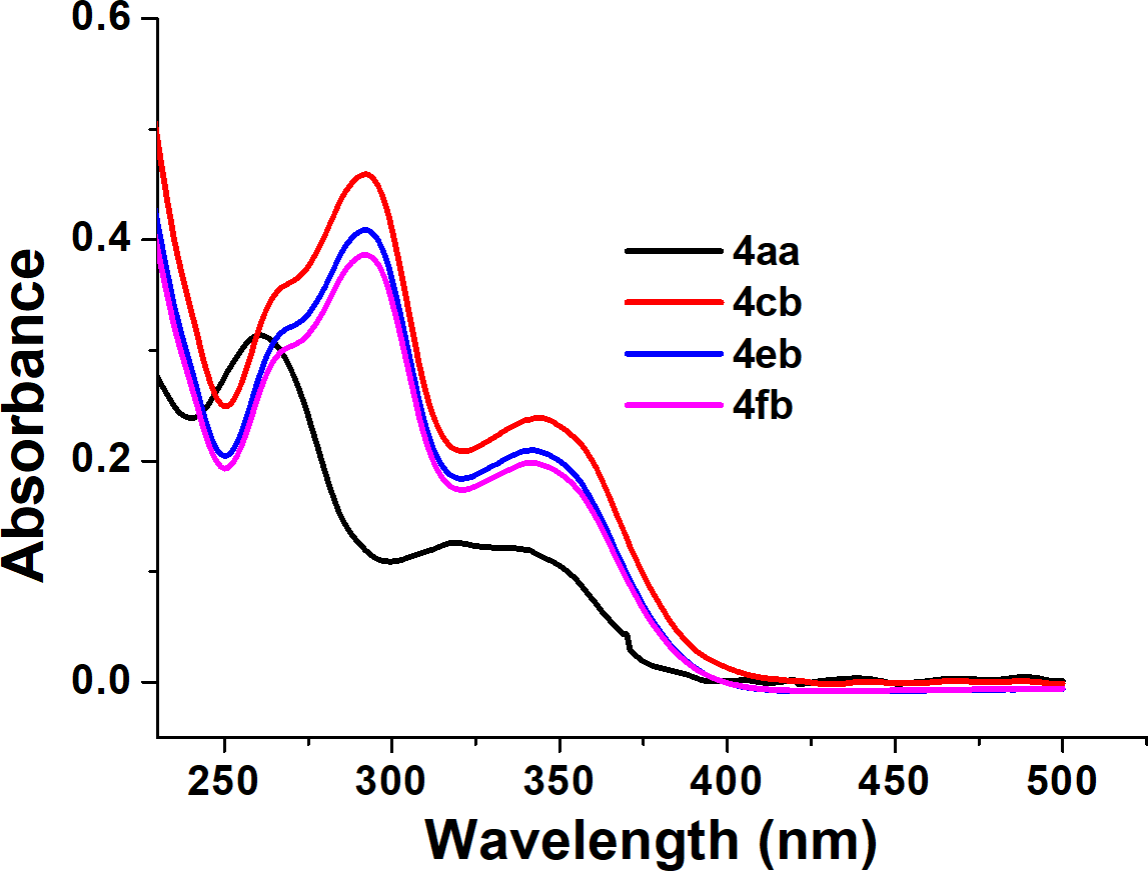
UV–vis absorption spectra of selected synthesized compounds **4aa**, **4cb**, **4eb**, and **4fb** from 225–500 nm.

One of the commercially important 2-hydroxybenzophenones, oxybenzone, is widely used as an ingredient in sunscreen lotions, due to its ability to absorb UV light, both in the UV-A and UV-B region [28–29]. However, oxybenzone, having a 4-methoxy substituent, has been reported to be associated with allergy reactions, Hirschsprung's disease, as endocrine disruptor, and toxic to the environment [30]. This has led researchers to search for alternatives, particularly regarding the backbone of 2-hydroxybenzophenones [11]. As reported earlier, 5-substituted-2-hydroxybenzophenones showed a better photoantioxidant ability, and can be an alternative to the commercially used UV-absorbers. Towards this, the UV absorption properties of the synthesized compounds were evaluated (Figure 6 and Figure S30 in Supporting Information File 1). UV absorption spectra of the most promising compounds (**4aa**, **4cb**, **4eb**, and **4fb**) are depicted in Figure 6.

The abilities of the synthesized compounds for use as a protector from UV radiation were evaluated based on several parameters as tabulated in Table 2. Initially, the λ_max_ values for different absorption peaks were calculated. The critical wavelength (λ_c_) were calculated using the Diffey method [31]. Here, λ_c_ values higher than 370 nm qualify a substrate to be used for extensive UV protection [11]. Further, the in vitro sun protection factor (SPF) was calculated for the compounds to determine their suitability as UV-protector.

**Table 2:** Optical properties of the compounds **4aa**–**ma**.

Compound	λ_max_ (nm)	ε (L mol^−1^ cm^−1^)	λ_C_ (nm)	Broad spectrum	UV-A/UV-B	SPF

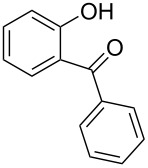 **4aa**	261	7856	387	Y	0.91	10.45
	334	3051				

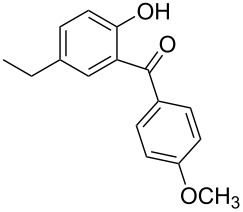 **4cb**	291	11495	380	Y	0.64	10.55
	345	6050				

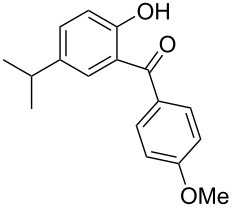 **4eb**	292	10190	365	Y	1.17	10.79
	243	5223				

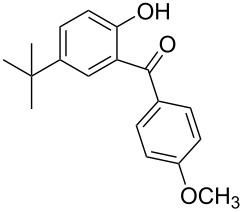 **4fb**	292	9650	364	Y	0.42	11.02
	342	4940				

Compounds **4cb**, **4eb**, and **4fb** showed SPF values comparable with the commercially available sunscreen product containing structurally similar oxybenzone (calculated SPF value 11.74) [32]. The data for the compounds with the most promising SPF values, **4aa**, **4cb**, **4eb**, and **4fb**, are tabulated in Table 2, and compiled data for all compounds are included in Supporting Information File 1, Table S12. As can be seen from Table 2, an electron-donating substituent on either of the benzene rings increases the compound’s UV protection abilities, via increasing the electron density. However, the presence of a strong-electron donating substituent, e.g., a -OCH_3_ on the benzene ring attached to the carbonyl center has a more pronounced effect on the UV-protection abilities (**4bb**, **4cb**, **4eb** and **4fb**) since it increases the electron density on the carbonyl center, and hence the hydrogen bonding between the carbonyl and the phenol residue is weakened. A similar phenomenon has been reported for 4**′**-substituted- 2-hydroxybenzophenones [6]. On the contrary, a strong electron-withdrawing group viz. –F at the 4'-position (**4fd**) rendered the compound to be a poor UV-protector. It was also observed that the presence of an alkoxy group at the 5-position of the 2-hydroxybenzophenones (**4ga**, **4ha** and **4ia**) did not improve the UV-protection abilities to an appreciable extent, and the SPF values decreased with increasing alkyl chain length.

## Conclusion

In conclusion, we have developed a transition-metal-free procedure to afford substituted 2-hydroxybenzophenones in good to excellent yields. The method utilizes hydroperoxide-generated in situ autoxidation of tetrahydrofuran. The mechanism of the transformation of benzofuranone to benzophenone are proposed based on control experiments. Further, the UV-protection abilities of the synthesized benzophenones were evaluated mathematically. Although the mathematical procedure adopted herein is a very preliminary way to assess the UV-protection abilities of the synthesized compounds, the data obtained clearly emphasize that 5′-substituted 2-hydroxybenzophenones, with an electron-donating group at the 4′-position, are good candidates for further evaluation in vitro and in vivo, after validating possible sunscreen formulations which improve the effects in a synergistic way. We believe that this work will open up avenues towards evaluating 5′-substituted 2-hydroxybenzophenones as efficient UV-protectors.

## Experimental

All chemicals were procured from Sigma-Aldrich (AR grade) and used without any further purification. All solvents were purchased from Merck, and were distilled or dried, wherever applicable, following standard procedures. The organic extracts were dried over anhydrous Na_2_SO_4_. FTIR spectra were recorded as films with a Bruker Tensor II spectrophotometer. The ^1^H and ^13^C NMR spectra were recorded with a Varian 500 MHz NMR spectrometer, and were processed using Bruker TOPSPIN software. Melting points (mp) were measured on a Büchi B-540 apparatus. X-ray data were collected either on an Agilent Supernova system equipped with a microfocus Cu source (λ = 1.54184 Å) and a titan CCD detector, or on a XtaLAB Synergy, Dualflex, HyPix four-circle diffractometer with a micro-focus sealed X-ray tube using a mirror as monochromator and a HyPix detector. The elemental analyses were carried out with an Elementar Vario micro cube.

**Generation of hydroperoxide in THF.** 35 mL of freshly distilled THF were heated under open atmosphere at 50 °C for 4 h. After 4 h, it was allowed to cool to room temperature, and THF was removed by distillation under vacuum to obtain the residue (8.2 g), which was further characterized using ^1^H NMR.

**General procedure for the synthesis of 3-arylbenzofuran-2(3*H*)-ones (3aa–ma).** 3-Arylbenzofuran-2(3*H*)-ones **3aa**–**ma** were synthesized using a method previously reported by us [20]. A mixture of SbCl_3_ (1.5 mmol), phenol/substituted phenol **1a**–**m** (5 mmol) and mandelic acid derivatives **2a**–**d** (6 mmol) was stirred and heated to 50 °C under nitrogen atmosphere until the mixture turned into a viscous oil. The viscous oil/paste was further heated to 140 °C until the reaction was complete (cf. TLC). The mixture was then brought to room temperature and a cold 10% aqueous NaHCO_3_ solution was added. The reaction mixture was diluted with EtOAc and filtered through a Celite bed. The filtrate was extracted with ethyl acetate (3 × 40 mL), the organic extract was washed with water and brine, and dried with Na_2_SO_4_. The organic extract was concentrated under vacuum, and the residue was purified using silica-gel column chromatography. Compounds **3aa** [21], **3ba** [21], **3ea** [21], **3eb** [21], **3fa** [21], **3fb** [22], **3ga** [21], and **3ja** [21] were synthesized using the similar method as reported previously by us.

**General procedure for the synthesis of 2-hydroxybenzophenones 4aa–ka**. 1 mmol Cs_2_CO_3_ was added to a solution of 3-arylbenzofuran-2(3*H*)-ones **3aa**–**ka** (1 mmol) in 2 mL of freshly distilled THF, and the mixture was heated at 50 °C with stirring under open atmosphere. The progress of the reaction was monitored by thin-layer chromatography (TLC). After completion of the reaction (3–4 h, cf. TLC), the mixture was cooled to room temperature and THF present in the reaction mixture was evaporated. The residue was purified by preparative TLC (hexane/EtOAc) to obtain pure 2-hydroxybenzophenones.

Cautions must be taken during the synthesis of 2-hydroxybenzophenones, since THF tends to form explosive compounds upon heating in open air atmosphere. Although the hydroperoxide formed during the heating immediately reacts with the substrates, and no accumulation of hydroperoxide has been observed, these reactions must be done under a fume hood, and the THF volume should be restricted to the recommended one. The reactions should be monitored continuously, and to be quenched as and when recommended in this section. Prolonged heating without monitoring is not recommended. A picture of the experimental set-up is included in Supporting Information File 1 (Figure S31).

### Single-crystal XRD

The data were collected using a similar method as reported by us earlier [23], using a XtaLAB Synergy, Dualflex, HyPix four-circle diffractometer, a micro-focus sealed X-ray tube and a HyPix detector. The data were corrected using SCALE3 ABSPACK and the structure were solved using SHELXT. Disordered moieties were refined using bond lengths restraints and displacement parameter restraints. Crystallographic data (including structure factors) for the structures reported in this paper have been deposited with the Cambridge Crystallographic Data Centre (CCDC Nos. 2348642, 2348641 and 2348646).

### UV-absorption studies

UV–vis absorbance spectra of the 5-substituted-2-hydroxybenzophenones **4aa**–**ka** were recorded in triplicates at room temperature (298 K) in ethanol at a concentration of 40 µM, in the range of 225–500 nm at 1 nm intervals [11]. The obtained data were corrected using calibration methods with ethanol as a blank. The critical wavelength (λ*_c_*) and UVA/UVB ratio were calculated using Equation 1 and Equation 2, respectively, as shown below.


[1] $$\int_{290}^{\lambda \text{c}}A(\lambda )d\lambda =0.9\int_{290}^{400}A(\lambda )d\lambda$$

[2] UVAUVB=∫320400A(λ)dλ∫320400dλ∫290320A(λ)dλ∫290320dλ

### Determination of SPF (sun protection factor)

An ethanolic solution of the compounds **4aa**–**ka** was prepared at a concentration of 200 μg/mL. The UV–vis absorption spectra of samples were measured in the range of 290 to 450 nm, every 5 nm, using ethanol as a blank [11]. The absorption data were obtained in three replicates at each point, and the SPF value was calculated using Equation 3.


[3] $$SPF_{spectrometric}=CF\times \sum_{290}^{320}EE(\lambda )\times I(\lambda )\times Abs(\lambda )$$

Where, *CF* (correction factor) = 10; *EE*(λ) is the erythemal effect spectrum; *I*(λ) is the solar intensity spectrum; *Abs*(λ) is the experimental absorbance values at corresponding wavelength. The values of [*EE*(λ) × *I*(λ)] are constants as shown in Table 3 [9].

**Table 3:** Values of [*EE*(λ) × *I*(λ)] used in calculating the SPF.

wavelength (λ) [nm]	*EE*(λ) × *I*(λ)

290	0.0150
295	0.0817
300	0.2874
305	0.3278
310	0.1874
315	0.0839
320	0.0180

total	1.000

## Supporting Information

File 1 Characterization data of compounds **3aa**–**ma**, **4aa–ma**, and **5**. ^1^H and ^13^C NMR spectra of **3aa**–**ma**, **4aa**–**ma**, and **5**; single crystal data of **4ja**, **4fb**, and **4ma**; UV–vis absorption spectra and optical properties of **4aa**–**ma**.

## Data Availability

The data that supports the findings of this study is available from the corresponding author upon reasonable request.
