# Supplementary material for: Transition-metal-free decarbonylation–oxidation of 3-arylbenzofuran-2(3H)-ones: access to 2-hydroxybenzophenones
Source: Beilstein J Org Chem. 2024 Oct 21;20:2655–67. doi: 10.3762/bjoc.20.223 (PMC11514451; doi:10.3762/bjoc.20.223)
Supplement: File 1 — Characterization data of compounds 3aa–ma, 4aa–ma, and 5. 1H and 13C NMR spectra of 3aa–ma, 4aa–ma, and 5; single crystal data of 4ja, 4fb, and 4ma; UV–vis absorption spectra and optical properties of 4aa–ma. [file Beilstein_J_Org_Chem-20-2655-s001.pdf]

BEILSTEIN JOURNAL OF ORGANIC CHEMISTRY

Supporting Information

for

Transition-metal-free decarbonylation–oxidation of 3-arylbenzofuran-2(3*H*)-ones: access to 2-hydroxybenzophenones

Bhaskar B. Dhotare, Seema V. Kanojia, Chahna K. Sakhiya, Amey Wadawale and Dibakar Goswami

Beilstein J. Org. Chem. **2024,** *20,* 2655–2667. doi:10.3762/bjoc.20.223

Characterization data of compounds 3aa–ma, 4aa–ma, and 5. ^{1}H and ^{13}C NMR spectra of 3aa–ma, 4aa–ma, and 5; single crystal data of 4ja, 4fb, and 4ma; UV–vis absorption spectra and optical properties of 4aa–ma

License and Terms: This is a supporting information file under the terms of the Creative Commons Attribution License (https://creativecommons.org/licenses/by/4.0). Please note that the reuse, redistribution and reproduction in particular requires that the author(s) and source are credited and that individual graphics may be subject to special legal provisions.
The license is subject to the *Beilstein Journal of Organic Chemistry* terms and conditions: (https://www.beilstein-journals.org/bjoc/terms)

Page No.	Description	
S2–S11		Analytical data of the synthesized compounds
S12	**Fig. S1**	^{1}H NMR showing formation of THF-hydroperoxide in situ in THF
S13	**Fig. S2**	^{1}H and ^{13}C NMR spectrum of **3bb**.
S14	**Fig. S3**	^{1}H and ^{13}C NMR spectrum of **3ca**.
S15	**Fig. S4**	^{1}H and ^{13}C NMR spectrum of **3cb**.
S16	**Fig. S5**	^{1}H and ^{13}C NMR spectrum of **3dc**.
S17	**Fig. S6**	^{1}H and ^{13}C NMR spectrum of **3fd**.
S18	**Fig. S7**	^{1}H and ^{13}C NMR spectrum of **3ha**.
S19	**Fig. S8**	^{1}H and ^{13}C NMR spectrum of **3ia**.
S20	**Fig. S9**	^{1}H and ^{13}C NMR spectrum of **3ka**.
S21	**Fig. S10**	^{1}H and ^{13}C NMR spectrum of **3la**.
S22	**Fig. S11**	^{1}H and ^{13}C NMR spectrum of **3ma**.
S23	**Fig. S12**	^{1}H and ^{13}C NMR spectrum of **4aa**.
S24	**Fig. S13**	^{1}H and ^{13}C NMR spectrum of **4ba**.
S25	**Fig. S14**	^{1}H and ^{13}C NMR spectrum of **4bb**.
S26	**Fig. S15**	^{1}H and ^{13}C NMR spectrum of **4ca**.
S27	**Fig. S16**	^{1}H and ^{13}C NMR spectrum of **4cb**.
S28	**Fig. S17**	^{1}H and ^{13}C NMR spectrum of **4dc**.
S29	**Fig. S18**	^{1}H and ^{13}C NMR spectrum of **4ea**.
S30	**Fig. S19**	^{1}H and ^{13}C NMR spectrum of **4eb**.
S31	**Fig. S20**	^{1}H and ^{13}C NMR spectrum of **4fa**.
S32	**Fig. S21**	^{1}H and ^{13}C NMR spectrum of **4fb**.
S33	**Fig. S22**	^{1}H and ^{13}C NMR spectrum of **4fd**.
S34	**Fig. S23**	^{1}H and ^{13}C NMR spectrum of **4ga**.
S35	**Fig. S24**	^{1}H and ^{13}C NMR spectrum of **4ha**.
S36	**Fig. S25**	^{1}H and ^{13}C NMR spectrum of **4ia**.
S37	**Fig. S26**	^{1}H and ^{13}C NMR spectrum of **4ja**.
S38	**Fig. S27**	^{1}H and ^{13}C NMR spectrum of **4ka**.
S39	**Fig. S28**	^{1}H and ^{13}C NMR spectrum of **4ma**.
S40	**Fig. S29**	^{1}H and ^{13}C NMR spectrum of **5**.
S41–S44	Single crystal XRD data for **4ja**	
S45–S52	Single crystal XRD data for **4fb**	
S53–S58	Single crystal XRD data for **4ma**	
S59	**Fig. S30**	UV–vis absorption spectra of synthesized compounds
S60	Table S12	Optical properties of the compounds **4aa**–**ma**.
S61	**Fig. S31**	Picture of the reaction set-up for the synthesis of **4aa**–**ma**.
S62	References	

1. Analytical data of the synthesized compounds

3-(4-Methoxyphenyl)-5-methylbenzofuran-2(3H)-one (**3bb**) [1]. Reaction of **1b** with **2b** following the general procedure followed by purification by column chromatography (silica gel, 0–15% EtOAc-hexane) yielded pure **3bb** as a colourless solid (yield: 0.92 g, 72%). mp 137-138 °C; FT-IR (neat) ν_{max} 2900, 1789, 1611, 1484, 1304, 1061 cm^{-1}; ^{1}H NMR (500 MHz, $CDCl_3$) δ (ppm): 7.16-7.11 (m, 3H), 7.07-7.04 (m, 1H), 7.00 (s, 1H), 6.91-6.87 (m, 2H), 4.80 (s, 1H), 3.79 (s, 3H), 2.32 (s, 3H); $^{13}C\{^{1}H\}$ NMR (125 MHz, $CDCl_3$) δ = 175.7, 159.4, 151.8, 134.1, 129.6, 129.3, 127.3, 127.2, 125.7, 114.5, 110.4, 55.3, 49.1, 21.0. Anal. Found: C, 75.30; H, 5.26. Calcd. for $C_{16}H_{14}O_3$: C, 75.58; H, 5.55 %.

5-Ethyl-3-phenylbenzofuran-2(3H)-one (**3ca**) [2]. Reaction of **1c** with **2a** following the general procedure followed by purification by column chromatography (silica gel, 0–15% EtOAc-hexane) yielded pure **3ca** as a colourless solid (yield: 0.87 g, 73%). mp 66-67 °C; FT-IR (neat) ν_{max} 2965, 1800, 1615, 1479, 1140 cm^{-1}; ^{1}H NMR (500 MHz, $CDCl_3$) δ (ppm): 7.41-7.31 (m, 3H), 7.25-7.22 (m, 2H), 7.21-7.16 (m, 1H), 7.12-7.08 (m, 1H), 7.03 (s, 1H), 4.88 (s, 1H), 2.63 (q, J = 7.5 Hz, 2H), 1.20 (t, J = 8.0 Hz, 3H); $^{13}C\{^{1}H\}$ NMR (125 MHz, $CDCl_3$) δ = 175.4, 152.0, 140.7, 135.3, 129.1, 128.6, 128.3, 128.1, 127.0, 124.6, 110.5, 49.9, 28.5, 15.8. Anal. Found: C, 80.67; H, 5.53. Calcd. for $C_{16}H_{14}O_2$: C, 80.65; H, 5.92 %.

5-Ethyl-3-(4-methoxyphenyl)benzofuran-2(3H)-one (**3cb**). Reaction of **1c** with **2b** following the general procedure followed by purification by column chromatography (silica gel, 0–15% EtOAc-hexane) yielded pure **3cb** as a colourless solid (yield: 0.94 g, 70%). mp 113-114 °C; FT-IR (neat) ν_{max} 2969, 1808, 1510, 1247, 1067 cm^{-1}; ^{1}H NMR (500 MHz, $CDCl_3$) δ (ppm): 7.19-7.12 (m, 3H), 7.09-7.06 (m, 1H), 7.01 (s, 1H), 6.91-6.87 (m, 2H), 4.80 (s, 1H), 3.79 (s, 3H), 2.62 (q, *J* = 7.5 Hz, 2H), 1.19 (t, *J* = 7.5 Hz, 3H); $^{13}C\{^{1}H\}$ NMR (125 MHz, $CDCl_3$) δ = 175.7, 159.5, 151.9, 140.7, 129.4, 128.5, 127.3, 127.2, 124.6, 114.5, 110.5, 55.3, 49.2, 28.5, 15.8. Anal. Found: C, 76.29; H, 5.64. Calcd. for $C_{17}H_{16}O_3$: C, 76.10; H, 6.01 %.

3-(4-Bromophenyl)-5-octylbenzofuran-2(3H)-one (**3dc**). Reaction of **1d** with **2c** following the general procedure followed by purification by column chromatography (silica gel, 0–15% EtOAc-hexane) yielded pure **3dc** as a colourless viscous liquid (yield: 1.42 g, 71%). FT-IR (neat) v_{max} 2957, 1807, 1485, 1189, 1060 cm^{-1}; ^{1}H NMR (500 MHz, $CDCl_3$) δ (ppm): 7.49 (d, *J* = 8.5 Hz, 2H), 7.19-7.15 (m, 1H), 7.14-7.07 (m, 3H), 6.99 (s, 1H), 4.82 (s, 1H), 2.58 (t, *J* = 7.5 Hz, 2H), 1.29-1.21 (m, 12H), 0.88 (t, *J* = 7.0 Hz, 3H); ^{13}C{^{1}H} NMR (125 MHz, $CDCl_3$) δ = 174.8, 152.0, 139.6, 134.3, 132.2, 130.0, 129.4, 126.2, 125.0, 122.3, 110.6, 49.3, 35.5, 31.8, 31.6, 29.3, 29.2, 29.1, 22.6, 14.0. Anal. Found: C, 65.73; H, 6.47. Calcd. for $C_{22}H_{25}BrO_2$: C, 65.84; H, 6.28 %.

5-(tert-Butyl)-3-(4-fluorophenyl)benzofuran-2(3H)-one (**3fd**). Reaction of **1f** with **2d** following the general procedure followed by purification by column chromatography (silica gel, 0–15% EtOAc-hexane) yielded pure **3fd** as a colourless viscous liquid (yield: 0.98 mg, 69%). FT-IR (neat) v_{max} 2960, 1813, 1559, 1508, 1056 cm^{-1}; ^{1}H NMR (500 MHz, $CDCl_3$) δ (ppm): 7.41 (d, *J* = 8.5 Hz, 1H), 7.25-7.19 (m, 3H), 7.14-7.04 (m, 3H), 4.86 (s, 1H), 1.31 (s, 9H); ^{13}C{^{1}H} NMR (125 MHz, $CDCl_3$) δ = 175.2, 163.5, 161.5, 151.7, 131.1, 131.0, 130.0, 129.8, 126.3, 126.2, 122.1, 116.1, 115.9, 49.2, 34.7, 31.5. Anal. Found: C, 76.31; H, 5.65. Calcd. for $C_{18}H_{17}FO_2$: C, 76.04; H, 6.03%.

5-Ethoxy-3-phenylbenzofuran-2(3H)-one (**3ha**). Reaction of **1h** with **2a** following the general procedure followed by purification by column chromatography (silica gel, 0–15% EtOAc-hexane) yielded pure **3ha** as a colourless solid **(**yield: 0.93 g, 73%). mp: 60 -61 °C; FT-IR (neat) v_{max} 2982, 1797, 1486, 1144, 1038 cm^{-1}; ^{1}H NMR (500 MHz, $CDCl_3$) δ (ppm): 7.38-7.31 (m, 3H), 7.24-7.21 (m, 2H), 7.07 (d, *J* = 9.0 Hz, 1H) 6.86 (dd, *J* = 2.5 and 3.0 Hz, 1H), 6.74 (d, *J* = 2.0 Hz, 1H), 4.86 (s, 1H), 3.98-3.93 (m, 2H), 1.38 (t, 6.5 Hz, 3H); ^{13}C{^{1}H} NMR (125 MHz, $CDCl_3$) δ = 175.4, 156.1, 147.7, 135.1, 129.1, 128.3, 128.2, 127.9, 115.2, 111.7, 111.3, 64.2, 50.4, 14.8. Anal. Found: C, 75.72; H, 5.21. Calcd. for $C_{16}H_{14}O_3$: C, 75.58; H, 5.55 %.

5-(Nonyloxy)-3-phenylbenzofuran-2(3H)-one (**3ia**). Reaction of **1i** with **2a** following the general procedure followed by purification by column chromatography (silica gel, 0–15% EtOAc-hexane) yielded pure **3ia** as a colourless viscous liquid (yield: 1.25 g, 71%); FT-IR (neat) ν_{max} 2923, 1806, 1485, 1394, 1060 cm^{-1}; ^{1}H NMR (500 MHz, $CDCl_3$) δ (ppm): 7.39-7.30 (m, 3H), 7.23 (d, *J* = 7.0 Hz, 2H), 7.09 (d, *J* = 9.0 Hz, 1H), 6.86 (dd, *J* = 2.5 and 2.5 Hz, 1H), 7.0 (d, *J* = 2.0 Hz, 1H), 4.85 (s, 1H), 3.87 (t, 6.5 Hz, 2H), 1.75-1.70 (m, 2H) 1.43-1.37 (m, 2H), 1.31-1.25 (m, 10H), 0.87 (t, 6.5 Hz, 3H); $^{13}C\{^{1}H\}$ NMR (125 MHz, $CDCl_3$) δ = 175.4, 156.4, 147.7, 135.2, 129.1, 128.3, 128.2, 127.8, 115.2, 111.7, 111.3, 68.8, 50.4, 31.8, 29.5, 29.3, 29.2, 26.0, 22.6, 14.1. Anal. Found: C, 78.62; H, 8.01. Calcd. for $C_{23}H_{28}O_3$: C, 78.38; H, 8.01 %.

7-(tert-Butyl)-5-methyl-3-phenylbenzofuran-2(3H)-one (**3ka**). Reaction of **1k** with **2a** following the general procedure followed by purification by column chromatography (silica gel, 0–15% EtOAc-hexane) yielded pure **3ka** as a colourless solid (yield: 1.04 g, 74%); mp: 160-161 ^{o}C; FT-IR (neat) ν_{max} 2959, 1781, 1453, 1267, 1072 cm^{-1}; ^{1}H NMR (500 MHz, $CDCl_3$) δ (ppm): 7.40-7.30 (m, 3H), 7.27-7.22 (m, 2H), 7.11 (s, 1H), 6.86 (s, 1H), 4.83 (s, 1H), 2.33 (s, 3H), 1.45 (s, 9H); $^{13}C\{^{1}H\}$ NMR (125 MHz, $CDCl_3$) δ = 175.5, 149.8, 135.6, 133.9, 133.7, 129.0, 128.3, 128.0, 127.4, 127.0, 123.1, 49.5, 34.1, 29.6, 21.2. Anal. Found: C, 81.69; H, 7.12. Calcd. for $C_{19}H_{20}O_2$: C, 81.40; H, 7.19 %.

5-Chloro-4,6-dimethyl-3-phenylbenzofuran-2(3H)-one (**3la**). Reaction of 4-chloro-3,5-dimethylphenol (5 mmol) and mandelic acid (6 mmol) following the general procedure followed by purification by column chromatography (silica gel, 0–15% EtOAc-hexane) yielded pure **3la** as a colourless solid (yield: 0.98 g, 72%). mp: 138-139 °C; FT-IR (neat) ν_{max} 2964, 1794, 1452, 1393, 1027 cm^{-1}; ^{1}H NMR (300 MHz, $CDCl_3$) δ (ppm): 7.39-7.34 (m, 3H), 7.18-7.16 (m, 2H), 6.97 (s, 1H), 4.82 (s, 1H), 2.45 (s, 3H), 2.04 (s, 3H); $^{13}C\{^{1}H\}$ NMR (75 MHz, $CDCl_3$) δ = 174.8, 151.9, 137.9, 134.4, 133.9, 130.4, 129.3, 128.3, 128.1, 124.4, 110.6, 49.9, 21.5, 17.1. Anal. Found: C, 70.62; H, 4.45. Calcd. for $C_{19}H_{20}O_2$: C, 70.46; H, 4.80 %.

3,7-Diphenylbenzofuran-2(3H)-one (**3ma**). Reaction of [1,1'-biphenyl]-2-ol (5 mmol) and mandelic acid (6 mmol) following the general procedure followed by purification by column chromatography (silica gel, 0–15% EtOAc-hexane) yielded pure **3ma** as a highly viscous liquid (yield: 1.07 g, 75%). FT-IR (neat) ν_{max} 2961, 1792, 1293, 1058 cm^{-1}; ^{1}H NMR (500 MHz, $CDCl_3$) δ (ppm): 7.77-7.65 (two d, 3H, *J* = 7.5 Hz), 7.57-7.51 (d, 1H, *J* = 8.0 Hz), 7.51-7.46 (t, 2H, *J* = 7.5 Hz), 7.43-7.32 (m, 4H), 7.31-7.23 (m, 2H), 7.21-7.16 (m, 1H), 4.97 (s, 1H); ^{13}C{^{1}H} NMR (75 MHz, $CDCl_3$) δ = 175.1, 152.4, 150.9, 137.1, 135.3, 135.1, 130.6, 130.3, 129.5, 129.4, 129.3, 129.3=2, 129.1, 129.0, 128.9, 128.8, 128.7, 128.6, 128.5, 128.4, 128.3, 128.1, 127.9, 127.8, 127.6, 125.1, 124.9, 124.2, 120.8, 115.9, 115.8, 49.8. Anal. Found: C, 83.72; H, 4.68. Calcd. for $C_{19}H_{20}O_2$: C, 83.90; H, 4.93 %.

(2-Hydroxyphenyl)(phenyl)methanone (**4aa**) [3]. Reaction of **3aa** following the general procedure followed by purification by column chromatography (silica gel, 0–20% EtOAc-hexane) yielded pure **4aa** as a colourless viscous liquid (yield: 168 mg, 85%). FT-IR (neat) ν_{max} 3059, 2957, 1625, 1444, 1032 cm^{-1}; ^{1}H NMR (500 MHz, $CDCl_3$) δ (ppm): 12.03 (s, 1H), 7.70-7.66 (m, 2H), 7.62-7.56 (m, 2H), 7.51 (t, J = 7.0 Hz, 3H), 7.07 (d, J = 8.5 Hz, 1H), 6.88 (t, J = 7.5, Hz, 1H); ^{13}C{^{1}H} NMR (125 MHz, $CDCl_3$) δ = 201.6, 163.2, 137.9, 136.3, 133.6, 131.9, 129.1, 128.3, 119, 118.6, 118.4. Anal. Found: C, 78.44; H, 5.14. Calcd. for $C_{13}H_{10}O_2$: C, 78.77; H, 5.09 %.

(2-Hydroxy-5-methylphenyl)(phenyl)methanone (**4ba**) [3]. Reaction of **3ba** following the general procedure followed by purification by column chromatography (silica gel, 0–20% EtOAc-hexane) yielded pure **4ba** as a colourless solid (Yield: 193 mg, 91%). mp: 81-82 °C; FT-IR (neat) ν_{max} 3200, 2918, 1625, 1445, 1225, 958 cm^{-1}; ^{1}H NMR (500 MHz, $CDCl_3$) δ (ppm): 11.85 (s, 1H), 7.69-7.64 (m, 2H), 7.62-7.56 (m, 1H), 7.54-7.48 (m, 2H), 7.37-7.30 (m, 2H), 6.98 (d, J = 9.0 Hz, 1H), 2.25 (s, 3H); ^{13}C{^{1}H} NMR (125 MHz, $CDCl_3$) δ = 201.6, 161.1, 138.0, 137.3, 131.2, 131.7, 129.1, 128.3, 127.7, 118.7, 118.1, 20.4. Anal. Found: C, 79.30; H, 5.47. Calcd. for $C_{14}H_{12}O_2$: C, 79.23; H, 5.70 %.

3-(4-Methoxyphenyl)-5-methylbenzofuran-2(3H)-one (**4bb**) [4]. Reaction of **3bb** following the general procedure followed by purification by column chromatography (silica gel, 0–20% EtOAc-hexane) yielded pure **4bb** as a colourless solid (yield: 199 mg, 82%). mp: 109-110 °C; FT-IR (neat) ν_{max} 2917, 1631, 1478, 1248, 1024 cm^{-1}; ^{1}H NMR (500 MHz, $CDCl_3$) δ (ppm): 11.75 (s, 1H), 7.70 (d, *J* = 8.5 Hz, 2H), 7.39 (s, 1H), 7.29 (d, *J* = 8,5 Hz, 1H), 7.00-6.95 (m, 3H), 3.89 (s, 3H), 2.26 (s, 3H); ^{13}C{^{1}H} NMR (125 MHz, $CDCl_3$) δ = 200.0, 162.8, 160.8, 136.7, 132.9, 131.7, 130.5, 127.6, 119.1, 118.0, 113.6, 55.4, 20.5. Anal. Found: C, 74.30; H, 5.65. Calcd. for $C_{15}H_{14}O_3$: C, 74.36; H, 5.82 %.

(5-Ethyl-2-hydroxyphenyl)(phenyl)methanone (**4ca**) [3]. Reaction of **3ca** following the general procedure followed by purification by column chromatography (silica gel, 0–20% EtOAc-hexane) yielded pure **4ca** as a colourless viscous liquid (yield: 190 mg, 84%). mp: 109-110 °C; FT-IR(neat) ν_{max} 2964, 1630, 1481, 950 cm^{-1}; ^{1}H NMR (500 MHz, $CDCl_3$) δ (ppm): 11.86 (s, 1H), 7.68 (d, *J* = 7.5 Hz, 2H), 7.59 (t, *J* = 7.5 Hz, 1H), 7.52 (t, *J* = 7.5 Hz, 2H), 7.40-7.34 (m, 2H), 7.01 (d, *J* = 8.0 Hz, 1H), 2.55 (q, *J* = 7.5 Hz, 2H), 1.16 (t, *J* = 7.5 Hz, 3H); ^{13}C{^{1}H} NMR (125 MHz, $CDCl_3$) δ = 200.6, 161.3, 138.1, 136.2, 134.3, 132.1, 131.8, 129.1, 128.3, 118.8, 118.2, 27.9, 15.7. Anal. Found: C, 79.35; H, 6.25. Calcd. for $C_{15}H_{14}O_2$: C, 79.62; H, 6.24%.

(5-Ethyl-2-hydroxyphenyl)(4-methoxyphenyl)methanone (**4cb**). Reaction of **3cb** following the general procedure followed by purification by column chromatography (silica gel, 0–20% EtOAc-hexane) yielded pure **4cb** as a colourless viscous liquid (yield: 184 mg, 72%); FT-IR (neat) ν_{max} 2959, 1627, 1481, 1243, 1028 cm^{-1}; ^{1}H NMR (500 MHz, $CDCl_3$) δ (ppm): 11.77 (s, 1H), 7.72 (d, *J* = 8.5 Hz, 2H), 7.42 (d, *J* = 2.0 Hz, 1H), 7.34 (dd, *J* = 2.0 and 6.5 Hz, 1H), 7.03-6.97 (m, 3H), 3.90 (s, 3H), 2.57 (q, *J* = 6.5 Hz, 2H), 1.18 (t, *J* = 7.5 Hz, 3H); ^{13}C{^{1}H} NMR (125 MHz, $CDCl_3$) δ = 200.0, 162.8, 160.9, 135.6, 134.1, 131.8, 131.7, 130.5, 119.1, 118.1, 113.6, 55.5, 27.9, 15.7. Anal. Found: C, 74.97; H, 6.31. Calcd. for $C_{16}H_{16}O_3$: C, 74.98; H, 6.29 %.

(4-Bromophenyl)(2-hydroxy-5-octylphenyl)methanone (**4dc**). Reaction of **3dc** following the general procedure followed by purification by column chromatography (silica gel, 0–20% EtOAc-hexane) yielded pure **4dc** as a colourless viscous liquid (yield: 284 mg, 73%); FT-IR (neat) v_{max} 2923, 1630, 1480, 1244, 1012 cm^{-1}; ^1H NMR (500 MHz, $CDCl_3$) δ (ppm): 11.68 (s, 1H), 7.65 (d, *J* = 8.5 Hz, 2H), 7.54 (d, *J* = 8.5 Hz, 2H), 7.34 (dd, *J* = 1.5 and 8.5 Hz, 1H), 7.29-7.27 (m, 1H), 7.26-7.25 (m, 1H), 2.49 (t, *J* = 7.5 Hz, 2H), 1.64-1.47 (m, 2H), 1.33-1.19 (m, 10H), 0.86 (t, *J* = 7.0 Hz, 3H); $^{13}C\{^1H\}$ NMR (125 MHz, $CDCl_3$) δ = 200.3, 161.3, 137.0, 136.8, 133.2, 132.3, 130.7, 126.7, 118.6, 118.3, 34.9, 31.8, 31.5, 29.3, 29.2, 29.0, 22.6, 14.0. Anal. Found: C, 64.66; H, 6.51. Calcd. for $C_{21}H_{25}BrO_2$: C, 64.79; H, 6.47 %.

(2-Hydroxy-5-isopropylphenyl)(phenyl)methanone (**4ea**) [3]. Reaction of **3ea** following the general procedure followed by purification by column chromatography (silica gel, 0–20% EtOAc-hexane) yielded pure **4ea** as a colourless viscous liquid (yield: 185 mg, 77%); FT-IR (neat) v_{max} 3300, 2956, 1809, 1597, 1244, 1074 cm^{-1}; ^1H NMR (500 MHz, $CDCl_3$) δ (ppm): 11.87 (s, 1H), 7.69 (d, *J* = 7.5 Hz, 2H), 7.60 (t, 7.5 Hz, 1H), 7.52 (t, 7.0 Hz, 2H), 7.44-7.38 (m, 2H), 7.02 (d, *J* = 8.0 Hz, 1H) 2.84-2.79 (m, 1H), 1.18 (d, *J* = 7.0 Hz, 6H); $^{13}C\{^1H\}$ NMR (125 MHz, $CDCl_3$) δ = 201.5, 161.3, 138.9, 138.0, 134.7, 131.8, 130.8, 129.2, 128.3, 118.7, 118.2, 33.2, 23.9. Anal. Found: C, 80.15; H, 6.56. Calcd. for $C_{16}H_{16}O_2$: C, 79.97; H, 6.71 %.

(2-Hydroxy-5-isopropylphenyl)(4-methoxyphenyl)methanone (**4eb**). Reaction of **3eb** following the general procedure followed by purification by column chromatography (silica gel, 0–20% EtOAc-hexane) yielded pure **4eb** as a colourless viscous liquid (Yield: 195 mg, 72%). mp: 108-109 °C; FT-IR (neat) v_{max} 2959, 1627, 1337, 1028 cm^{-1}; ^1H NMR (500 MHz, $CDCl_3$) δ (ppm): 11.78 (s, 1H), 7.21 (d, *J* = 9.0 Hz, 2H), 7.45 (d, *J* = 2.0 Hz, 1H), 7.38-7.35 (m, 1H), 7.00-6.98 (m, 3H), 3.89 (s, 3H), 2.85-2.8(m, 1H), 1.19 (d, *J* = 6.5 Hz, 6H); $^{13}C\{^1H\}$ NMR (125 MHz, $CDCl_3$) δ = 199.9, 162.8, 160.9, 138.7, 134.2, 131.8, 130.5, 130.4, 118.9, 118.0, 113.6, 55.4, 33.2, 23.9. Anal. Found: C, 75.22; H, 6.45. Calcd. for $C_{17}H_{18}O_3$: C, 75.53; H, 6.71 %.

(5-(tert-Butyl)-2-hydroxyphenyl)(phenyl)methanone (**4fa**) [3]. Reaction of **3fa** following the general procedure followed by purification by column chromatography (silica gel, 0–20% EtOAc-hexane) yielded pure **4fa** as a colourless viscous liquid (yield: 214 mg, 84%); FT-IR (neat) ν_{max} 3400, 2959, 1629, 1482, 968 cm^{-1}; ^{1}H NMR (500 MHz, $CDCl_3$) δ (ppm): 11.86 (s, 1H), 7.69 (d, *J* = 7.5 Hz, 2H), 7.63-7.55 (m, 3H), 7.52 (t, *J* = 7.5 Hz, 2H), 7.02 (d, *J* = 8.0 Hz, 1H), 1.25 (s, 9H); $^{13}C\{^{1}H\}$ NMR (125 MHz, $CDCl_3$) δ = 201.6, 160.9, 141.3, 138.1, 133.9, 131.9, 129.8, 129.2, 128.3, 118.4, 117.9, 34.0, 31.2. Anal. Found: C, 80.50; H, 7.10. Calcd. for $C_{17}H_{18}O_2$: C, 80.28; H, 7.13 %.

(5-(tert-Butyl)-2-hydroxyphenyl)(4-methoxyphenyl)methanone (**4fb**). Reaction of **3fb** following the general procedure followed by purification by column chromatography (silica gel, 0–20% EtOAc-hexane) yielded pure **4fb** as a colourless solid (yield: 245 mg, 86%). mp: 92-93 °C; FT-IR (neat) ν_{max} 2919, 1626, 1565, 1029 cm^{-1}; ^{1}H NMR (500 MHz, $CDCl_3$) δ (ppm): 11.76 (s, 1H), 7.73 (d, *J* = 9.0 Hz, 2H), 7.62 (d, *J* = 2 Hz, 1H), 7.56-7.53 (m, 1H), 7.01 (d, *J* = 8.5 Hz, 3H), 3.91 (s, 3H), 1.27 (s, 9H); $^{13}C\{^{1}H\}$ NMR (125 MHz, $CDCl_3$) δ = 200.1, 162.9, 160.6, 141.1, 133.3, 131.8, 130.6, 129.6, 118.7, 117.8, 113.6, 55.5, 34.1, 31.3. Anal. Found: C, 75.87; H, 6.88. Calcd. for $C_{18}H_{20}O_3$: C, 76.03; H, 7.09 %.

(5-(tert-Butyl)-2-hydroxyphenyl)(4-fluorophenyl)methanone (**4fd**). Reaction of **3fd** following the general procedure followed by purification by column chromatography (silica gel, 0–20% EtOAc-hexane) yielded pure **4fd** as a colourless solid (yield: 150 mg, 55%). mp: 168-169 °C; FT-IR (neat) ν_{max} 3402, 2918, 1784, 1319, 1027 cm^{-1}; ^{1}H NMR (500 MHz, $CDCl_3$) δ (ppm):11.69 (s, 1H), 7.74-7.72 (m, 2H), 7.58-7.56 (m, 1H), 7.53 (d, *J* = 2.5 Hz 1H),7.21 (t, *J* = 9.0 Hz, 2H), 7.02 (d, *J* = 9.0 Hz 1H), 1.25 (s, 9H); $^{13}C\{^{1}H\}$ NMR (125 MHz, $CDCl_3$) δ = 200.0, 166.0, 163.9, 160.9, 141.4, 133.9, 131.8, 131.7, 129.4, 118.3, 118.0, 115.6, 115.4, 34.1, 31.2. Anal. Found: C, 75.17; H, 6.52. Calcd. for $C_{17}H_{17}FO_2$: C, 74.98; H, 6.29 %.

(2-Hydroxy-5-methoxyphenyl)(phenyl)methanone (**4ga**) [3]. Reaction of **3ga** following the general procedure followed by purification by column chromatography (silica gel, 0–20% EtOAc-hexane) yielded pure **4ga** as a colourless solid (yield: 178 mg, 78%). mp: 81-82 °C; FT-IR (neat) ν_{max} 2917, 1633, 1483, 1039 cm^{-1}; ^{1}H NMR (500 MHz, $CDCl_3$) δ (ppm): 11.59 (s, 1H), 7.69 (d, *J* = 8.0 Hz, 2H), 7.60 (t, *J* = 8.5 Hz, 1H), 7.51 (t, *J* = 7.5 Hz, 2H), 7.14 (d, *J* = 8.5, Hz, 1H), 7.08-6.99 (m, 2H), 3.70 (s, 3H); ^{13}C{^{1}H} NMR (125 MHz, $CDCl_3$) δ = 201.1, 157.5, 151.4, 137.9, 131.9, 129.1, 128.4, 124.0, 119.2, 118.7, 116.3, 55.9. Anal. Found: C, 73.54; H, 5.29. Calcd. for $C_{14}H_{12}O_3$: C, 73.67; H, 5.30 %.

(5-Ethoxy-2-hydroxyphenyl)(phenyl)methanone (**4ha**). Reaction of **3ha** following the general procedure followed by purification by column chromatography (silica gel, 0–20% EtOAc-hexane) yielded pure **4ha** as a colourless viscous liquid (yield: 174 mg, 72%). FT-IR (neat) ν_{max} 2980, 1632, 1445, 1046 cm^{-1}; ^{1}H NMR (500 MHz, $CDCl_3$) δ (ppm): 11.58 (s, 1H), 7.69 (d, *J* = 7.5 Hz, 2H), 7.59 (t, *J* = 7.5 Hz, 1H), 7.51 (t, *J* = 8.0 Hz, 2H), 7.14 (dd, *J* = 9.0 Hz, 1H), 7.05 (d, *J* = 3.0, Hz, 1H), 7.00 (d, *J* = 9.0 Hz, 1H), 3.89, (q, *J* = 7.0 Hz, 2H), 1.35 (t, *J* = 7.0 Hz, 3H); ^{13}C{^{1}H} NMR (125 MHz, $CDCl_3$) δ = 201.2, 157.4, 150.7, 137.9, 131.9, 129.0, 128.3, 124.7, 119.1, 118.7, 117.3, 64.3, 14.7. Anal. Found: C, 74.51; H, 6.02. Calcd. for $C_{15}H_{14}O_3$: C, 74.36; H, 5.82 %.

(2-Hydroxy-5-(nonyloxy)phenyl)(phenyl)methanone (**4ia**). Reaction of **3ia** following the general procedure followed by purification by column chromatography (silica gel, 0–20% EtOAc-hexane) yielded pure **4ia** as a colourless viscous liquid (Yield: 249 mg, 73%). FT-IR (neat) ν_{max} 2925, 1603, 1483, 1029 cm^{-1}; ^{1}H NMR (500 MHz, $CDCl_3$) δ (ppm): 11.56 (s, 1H), 7.68 (d, *J* = 7.0 Hz, 2H), 7.59 (t, *J* = 7.5 Hz, 1H), 7.51 (t, *J* = 8.0 Hz, 2H), 7.13 (dd, *J* = 3.0 and 9.0 Hz, 1H), 7.04 (d, *J* = 3.0 Hz, 1H), 7.99 (d, *J* = 4.0 Hz, 1H), 3.81, (t, *J* = 6.5 Hz, 2H), 1.72-1.66 (m, 2H), 1.41-1.35 (m, 2H), 1.32-1.24 (m, 10H), 0.86 (t, *J* = 7.0 Hz, 3H); ^{13}C{^{1}H} NMR (125 MHz, $CDCl_3$) δ = 201.2, 157.4, 150.9, 137.9, 131.9, 129.1, 128.3, 124.7, 119.1, 118.7, 117.3, 69.0, 31.8, 29.4, 29.3, 29.2, 29.1, 26.0, 22.6, 14.1. Anal. Found: C, 77.26; H, 8.07. Calcd. for $C_{22}H_{28}O_3$: C, 77.61; H, 8.29 %.

(5-Bromo-2-hydroxyphenyl)(phenyl)methanone (**4ja**). Reaction of **3ja** following the general procedure followed by purification by column chromatography (silica gel, 0–20% EtOAc-hexane) yielded pure **4ja** as a colourless solid (yield: 233 mg, 94%); mp: 111 -112 °C; IR (neat) v_{max} 3300, 2917, 1620, 1466, 1080 cm^{-1}; ^{1}H NMR (500 MHz, $CDCl_3$) δ (ppm): 11.93 (s, 1H), 7.72-7.65 (m, 3H), 7.64-7.51 (m, 4H), 6.99 (d, *J* = 8.5 Hz, 1H),; ^{13}C{^{1}H} NMR (125 MHz, $CDCl_3$) δ = 200.5, 162.1, 138.9, 137.2, 135.3, 132.4, 129.1, 128.6, 120.5, 120.4, 110.2. Anal. Found: C, 56.41; H, 3.64. Calcd. for $C_{13}H_9BrO_2$: C, 56.35; H, 3.27 %.

(3-(tert-Butyl)-2-hydroxy-5-methylphenyl)(phenyl)methanone (**4ka**). Reaction of **3ka** following the general procedure followed by purification by column chromatography (silica gel, 0–20% EtOAc-hexane) yielded pure **4ka** as a colourless viscous liquid (yield: 225 mg, 84%). FT-IR (neat) v_{max} 3556, 2956, 1621, 1431, 986 cm^{-1}; ^{1}H NMR (500 MHz, $CDCl_3$) δ (ppm): 12.7 (s, 1H), 7.64 (d, *J* = 7.5, Hz, 2H), 7.58 (t, *J* = 7.5, Hz, 1H), 7.50 (t, *J* = 8, Hz, 2H), 7.32 (d, *J* = 1.5, Hz, 1H), 7.20 (s, 1H), 2.23 (s, 3H), 1.45 (s, 9H); ^{13}C{^{1}H} NMR (125 MHz, $CDCl_3$) δ = 202.4, 160.8, 138.7, 138.4, 134.8, 131.4, 131.2, 128.2, 126.4, 118.6, 34.8, 29.3, 20.8. Anal. Found: C, 80.42; H, 7.32. Calcd. for $C_{18}H_{20}O_2$: C, 80.56; H, 7.51 %.

(2-Hydroxy-[1,1'-biphenyl]-3-yl)(phenyl)methanone (**4ma**). A solution of **3ma** in 2 mL of freshly distilled THF was heated at 50 °C in the presence of 1.0 mmol Cs_2CO_3 under open atmosphere. After completion of the reaction (cf. TLC), the mixture was cooled to room temperature and THF was evaporated under vacuum. The residue was purified by preparative TLC (hexane/EtOAc) to obtain pure **4ma** as a colourless solid (yield: 217 mg, 53% in two steps). mp: 102-103 °C; FT-IR (neat) v_{max} 2921, 1608, 1446, 1024 cm^{-1}; ^{1}H NMR (500 MHz, $CDCl_3$) δ (ppm): 12.56 (s, 1H), 7.74-7.69 (m, 2H), 7.66-7.57 (m, 5H), 7.53 (t, *J* = 8.5 Hz, 2H), 7.47 (t, *J* = 8.5 Hz, 2H), 7.38 (t, *J* = 6.5 Hz, 1H), 6.95 (t, *J* = 8.0 Hz, 1H). ^{13}C{^{1}H} NMR (125 MHz, $CDCl_3$) δ = 202.0, 160.5, 138.1, 137.2, 137.0, 133.0, 131.9, 131.2, 129.4, 129.2, 128.3, 128.2, 127.5, 119.3, 118.5. Anal. Found: C, 83.30; H, 5.23. Calcd. for $C_{19}H_{14}O_2$: C, 83.19 H, 5.14 %.

Synthesis of 5-chloro-3-hydroxy-4,6-dimethyl-3-phenylbenzofuran-2(3H)-one (**5**). As described before, a solution of **3na** (1.0 mmol) in 2 mL of freshly distilled THF was heated at 50 ℃ in the presence of 1.0 mmol Cs_2CO_3 under open atmosphere. After completion of the reaction (cf. TLC), the mixture was cooled to room temperature and THF was evaporated under vacuum. The residue was purified by preparative TLC (hexane/EtOAc) to obtain pure **5** as a colourless solid (yield: 216 mg, 75%). mp: 161-162 °C; FT-IR (neat) ν_{max} 3480, 2956, 1787, 1451, 1027 cm^{-1}; ^1H NMR (500 MHz, $CDCl_3$) δ (ppm): 7.38-7.31 (m, 4H), 6.97 (s, 1H), 3.34 (s, 1H), 2.44 (s, 3H), 2.13 (s, 1H). $^{13}C\{^1H\}$ NMR (125 MHz, $CDCl_3$) δ = 175.9, 151.4, 139.8, 137.5, 135.5, 131.3, 129.2, 128.9, 128.8, 126.1, 125.1, 111.0, 77.7, 21.6, 15.8. Anal. Found: C, 65.71; H, 4.25. Calcd. for $C_{16}H_{13}ClO_3$: C, 66.56 H, 4.54 %.

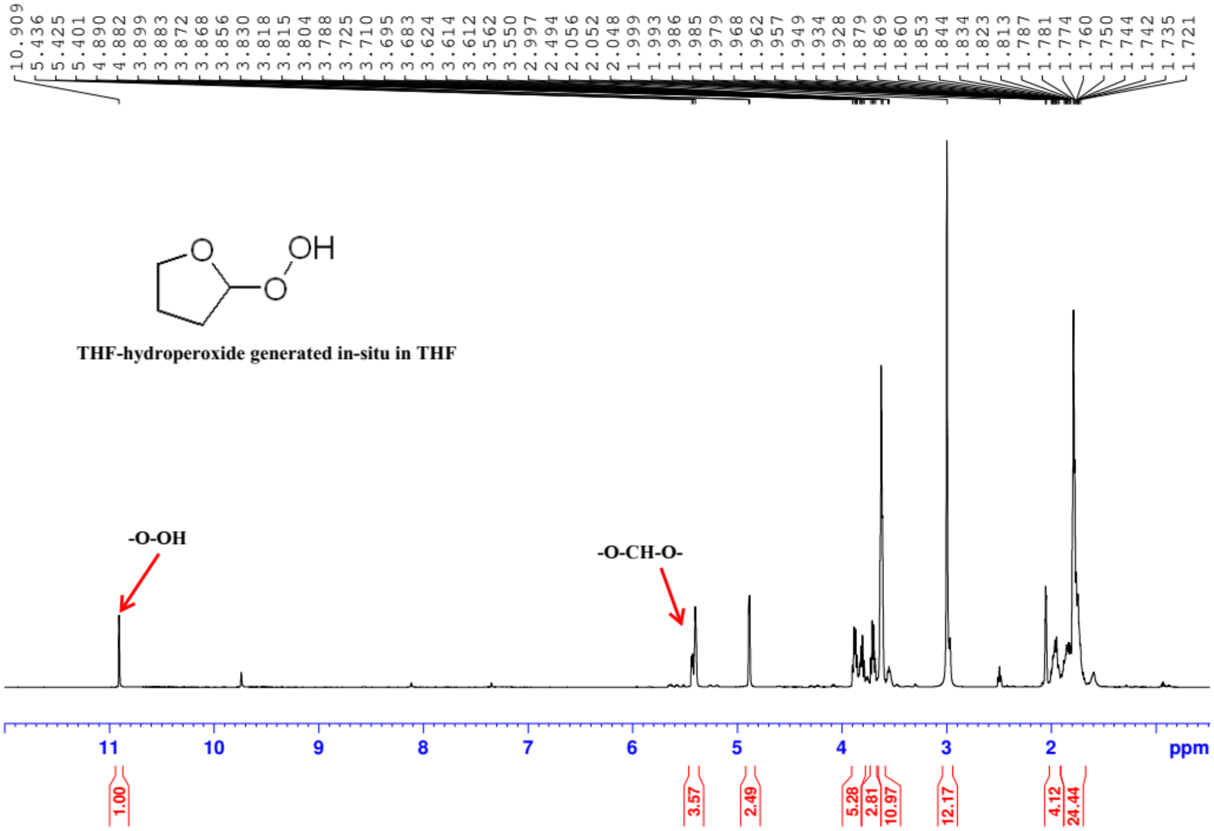

Figure S1. ^{1}H NMR showing formation of THF-hydroperoxide in situ in THF.

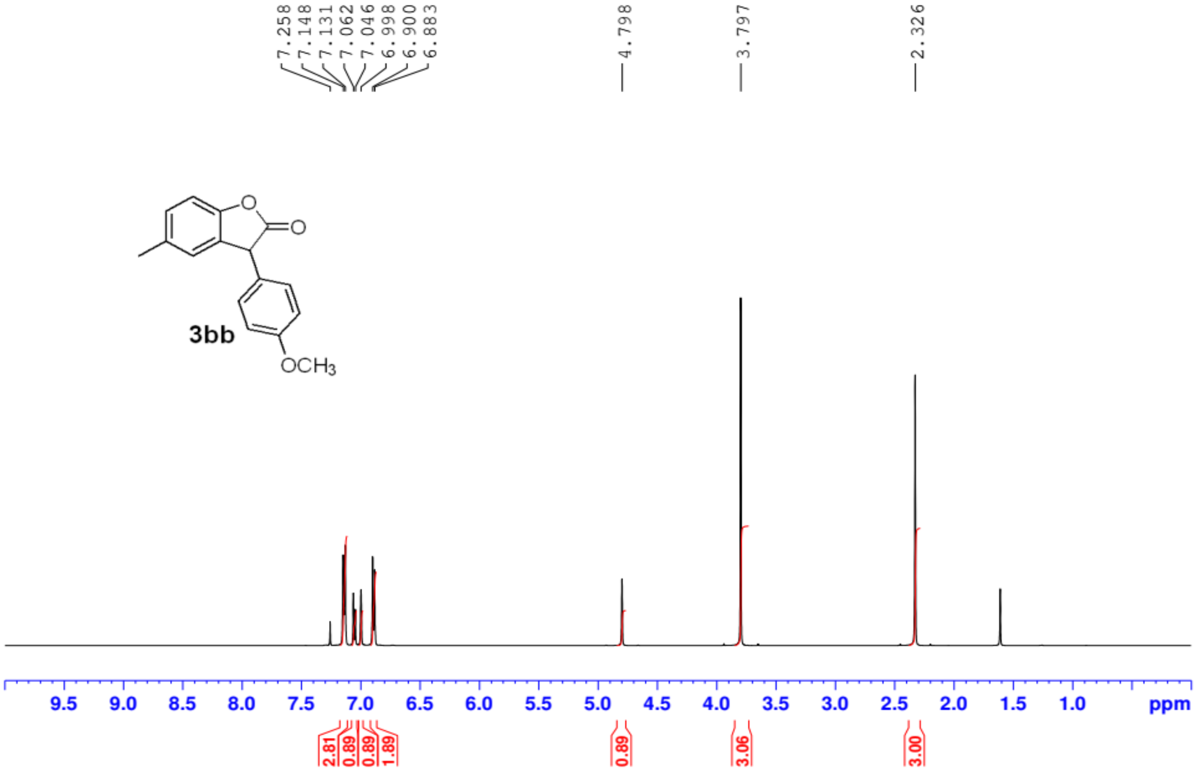

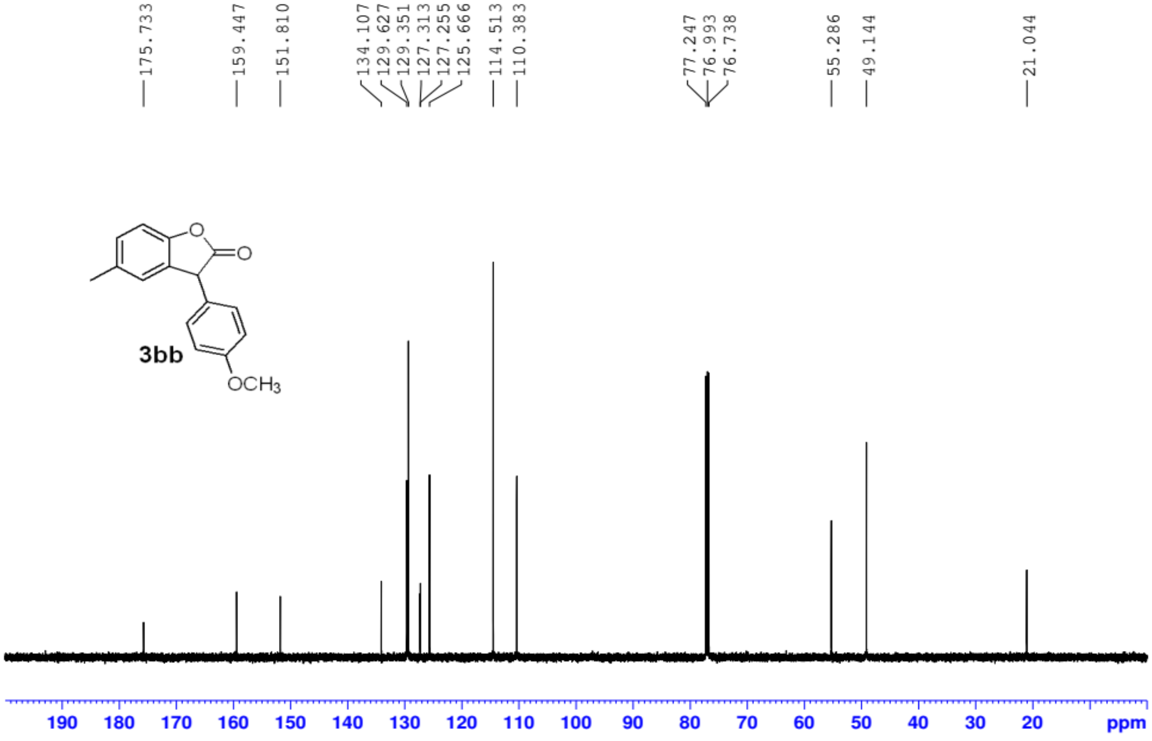

Figure S2. ^{1}H and ^{13}C NMR spectrum of **3bb**.

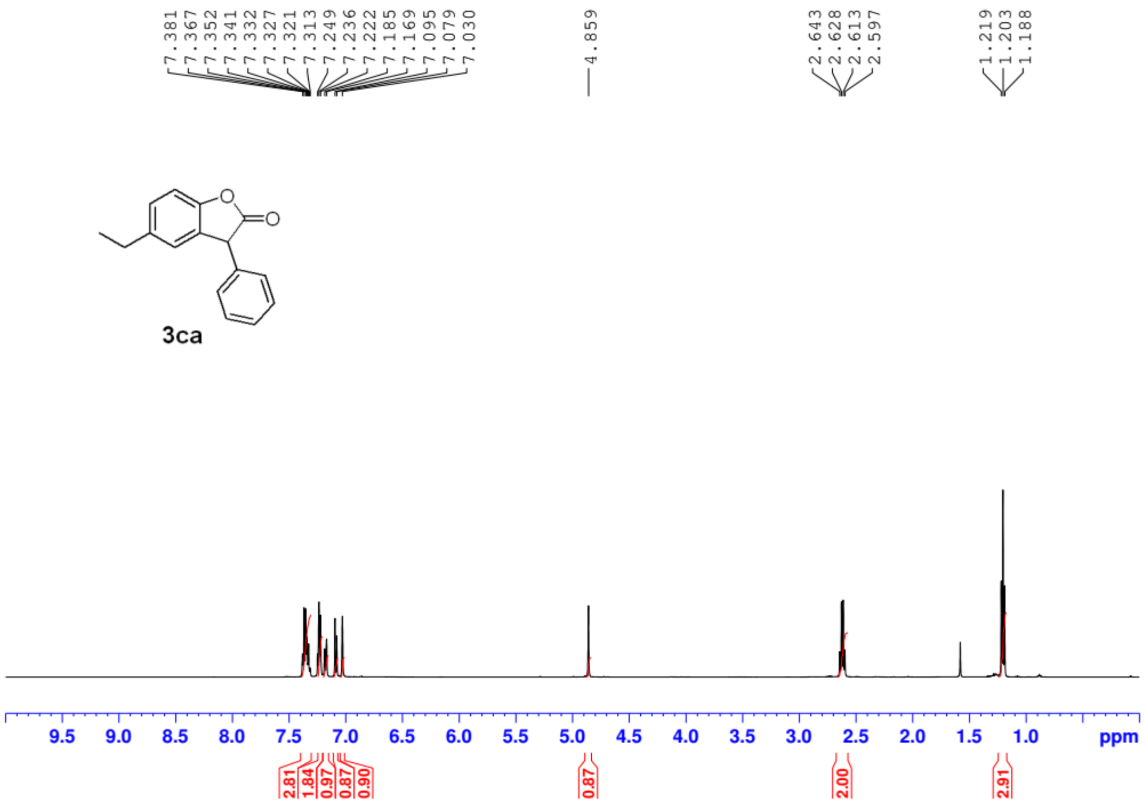

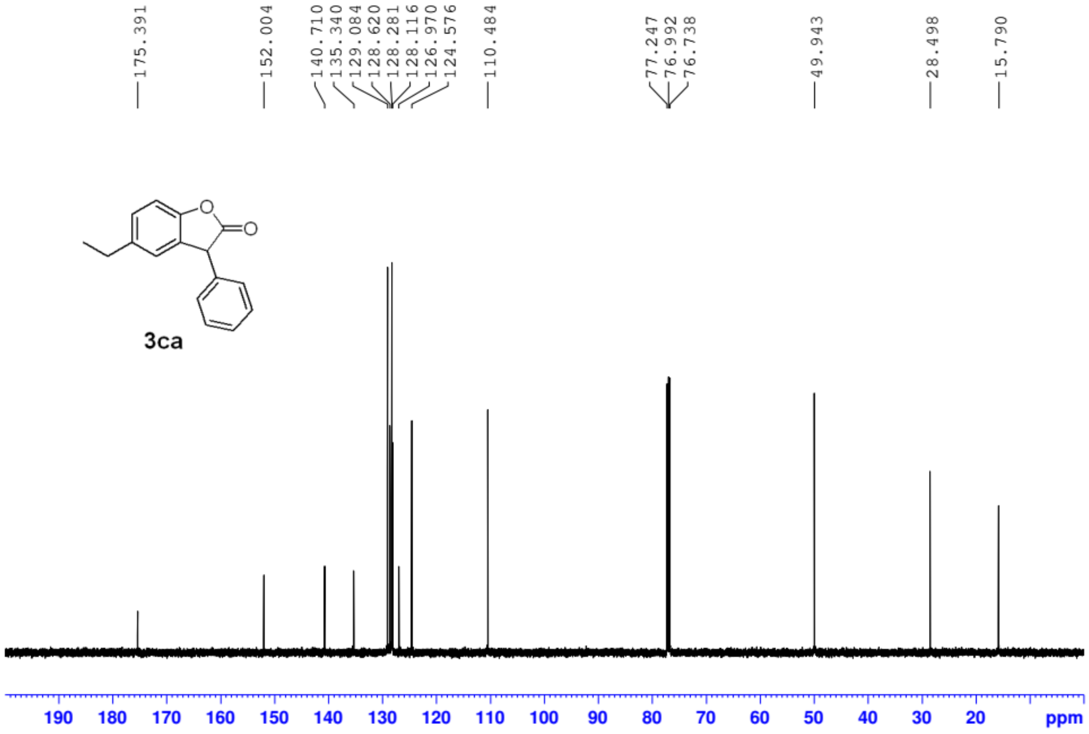

Figure S3. ^{1}H and ^{13}C NMR spectrum of **3ca**.

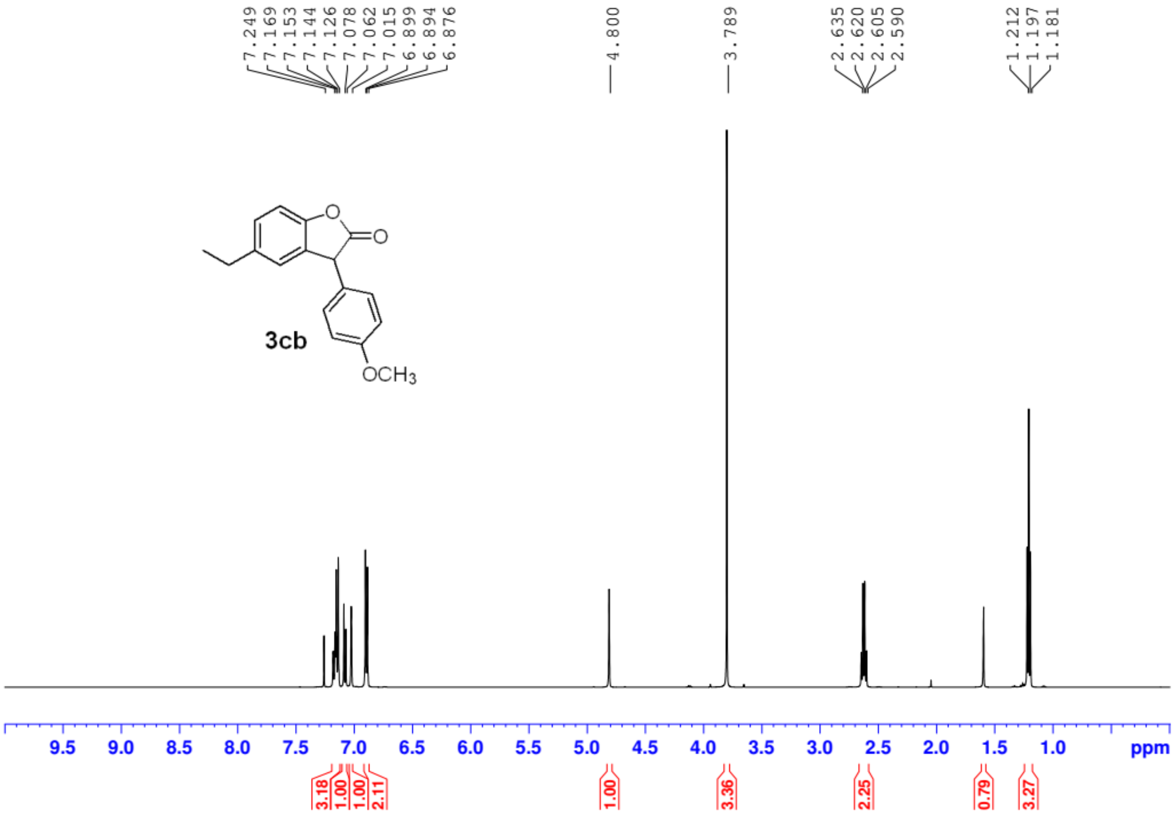

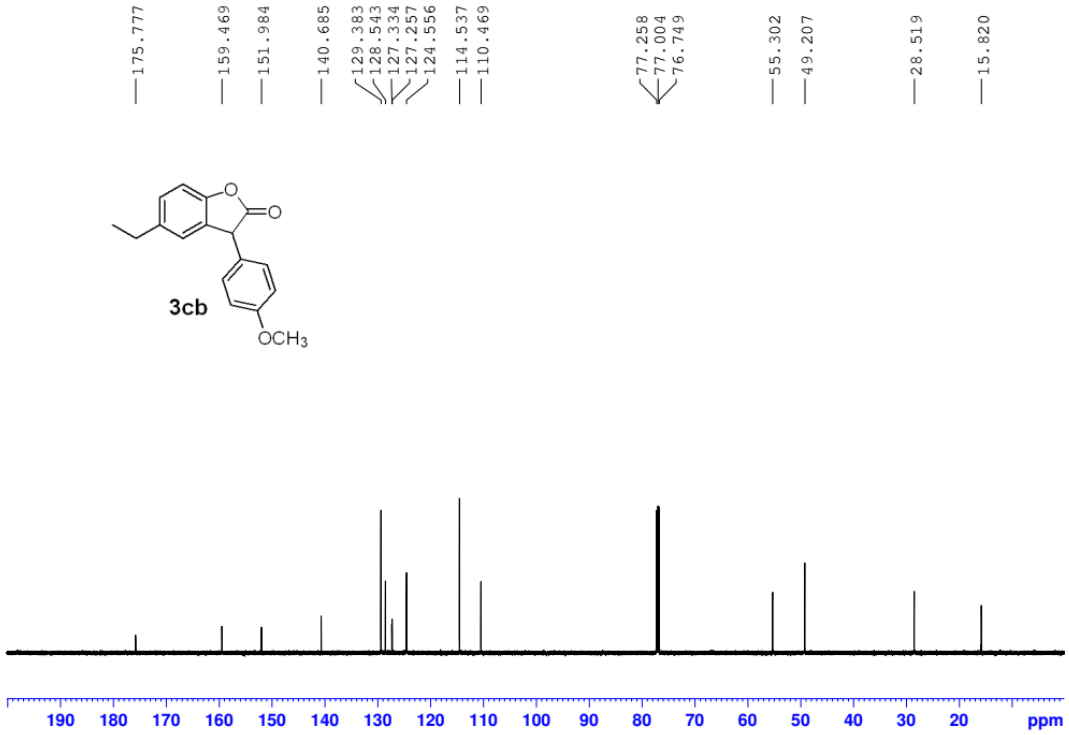

Figure S4. ^{1}H and ^{13}C NMR spectrum of **3cb**.

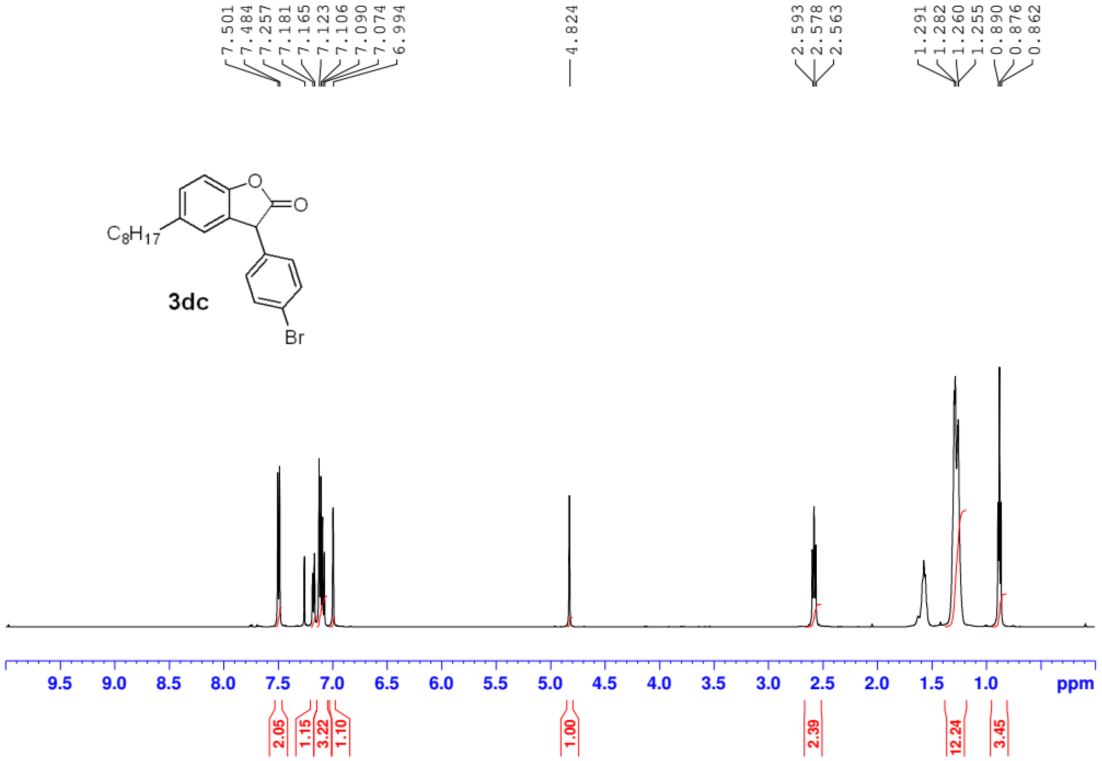

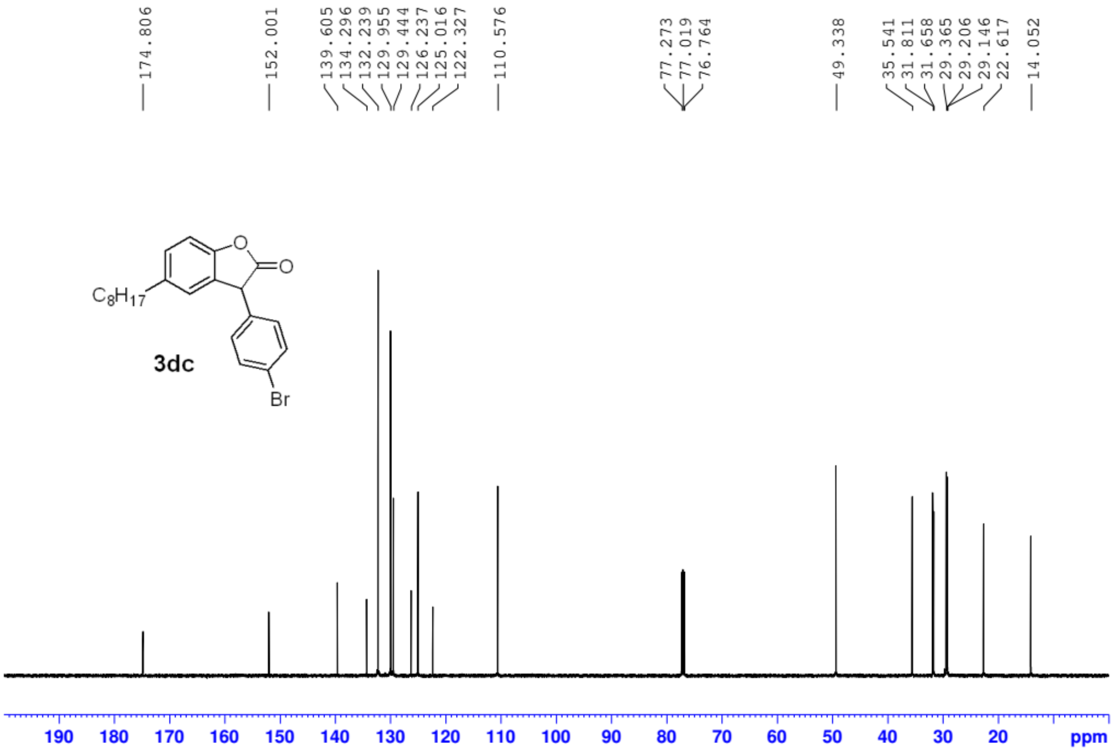

Figure S5. ^{1}H and ^{13}C NMR spectrum of **3dc**.

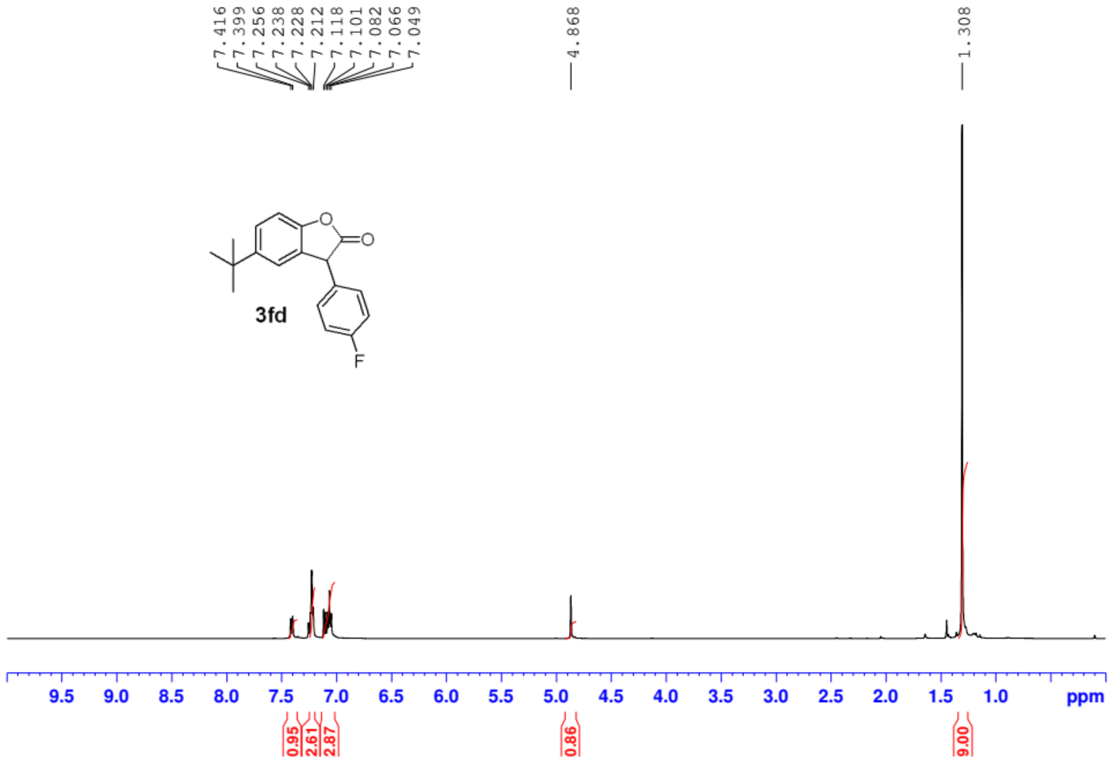

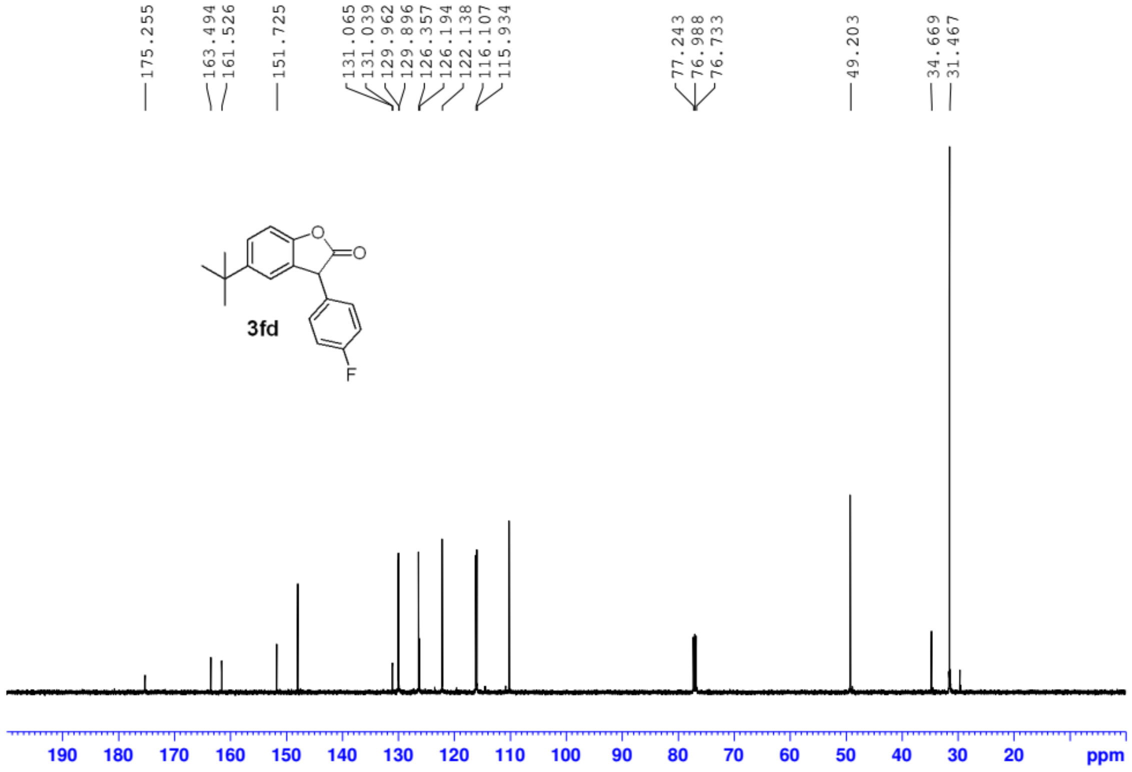

Figure S6. ^{1}H and ^{13}C NMR spectrum of **3fd**.

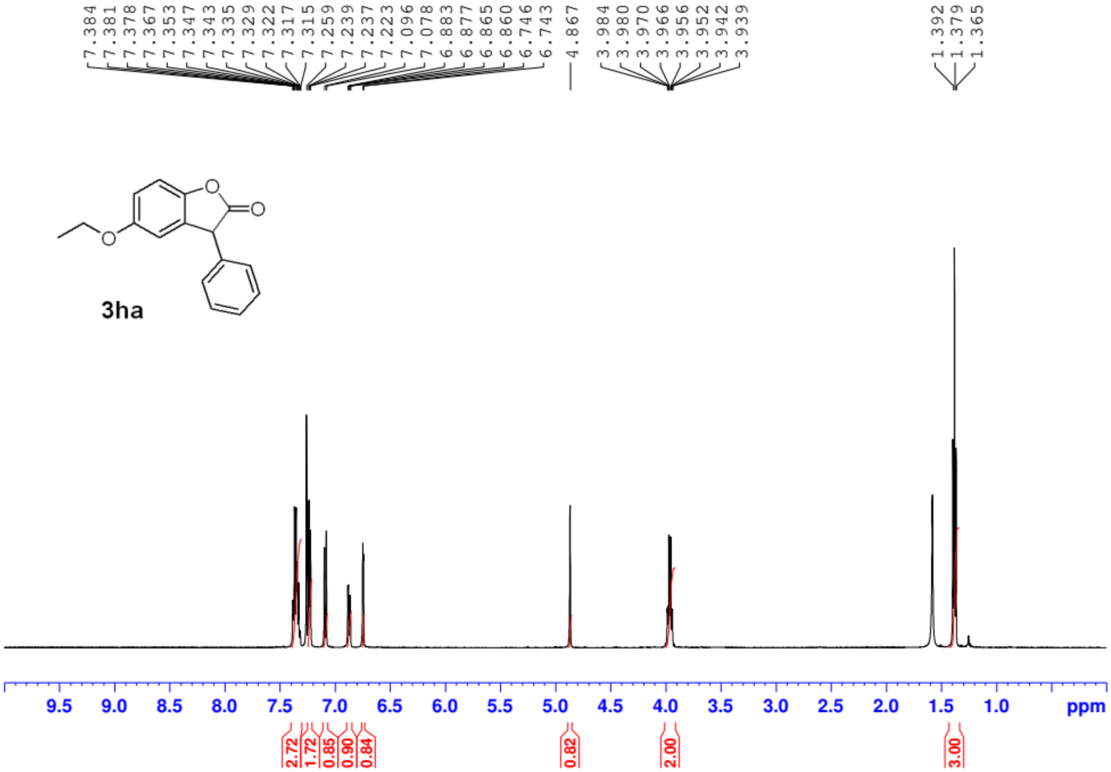

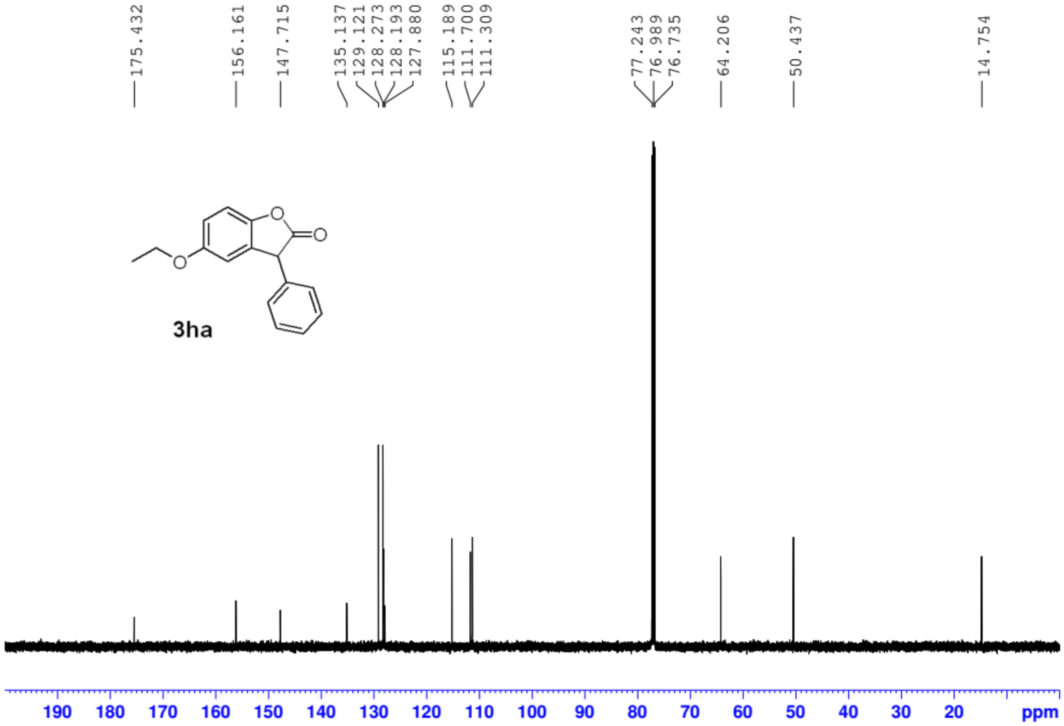

Figure S7. ^{1}H and ^{13}C NMR spectrum of **3ha**.

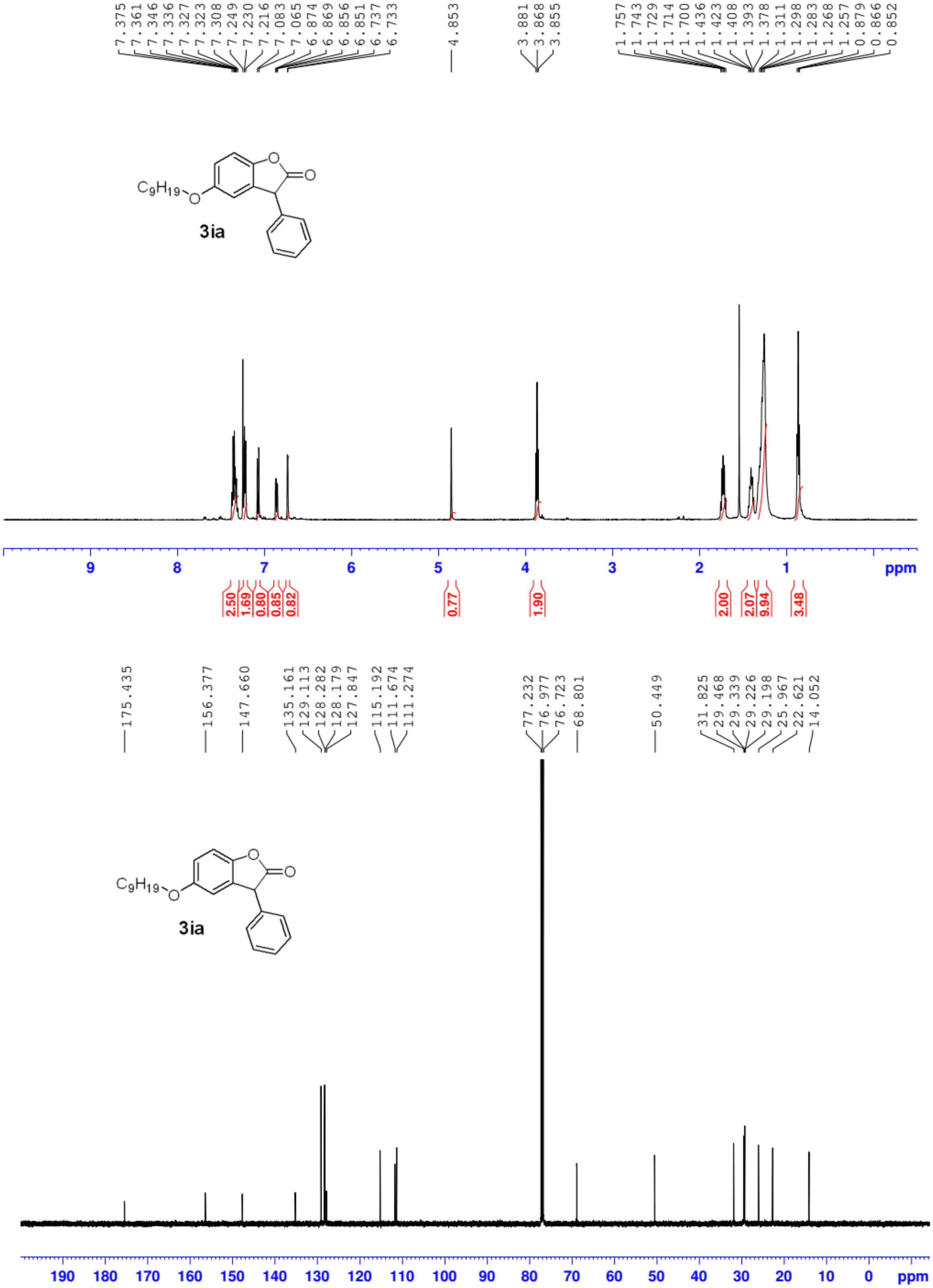

Figure S8. ^{1}H and ^{13}C NMR spectrum of **3ia**.

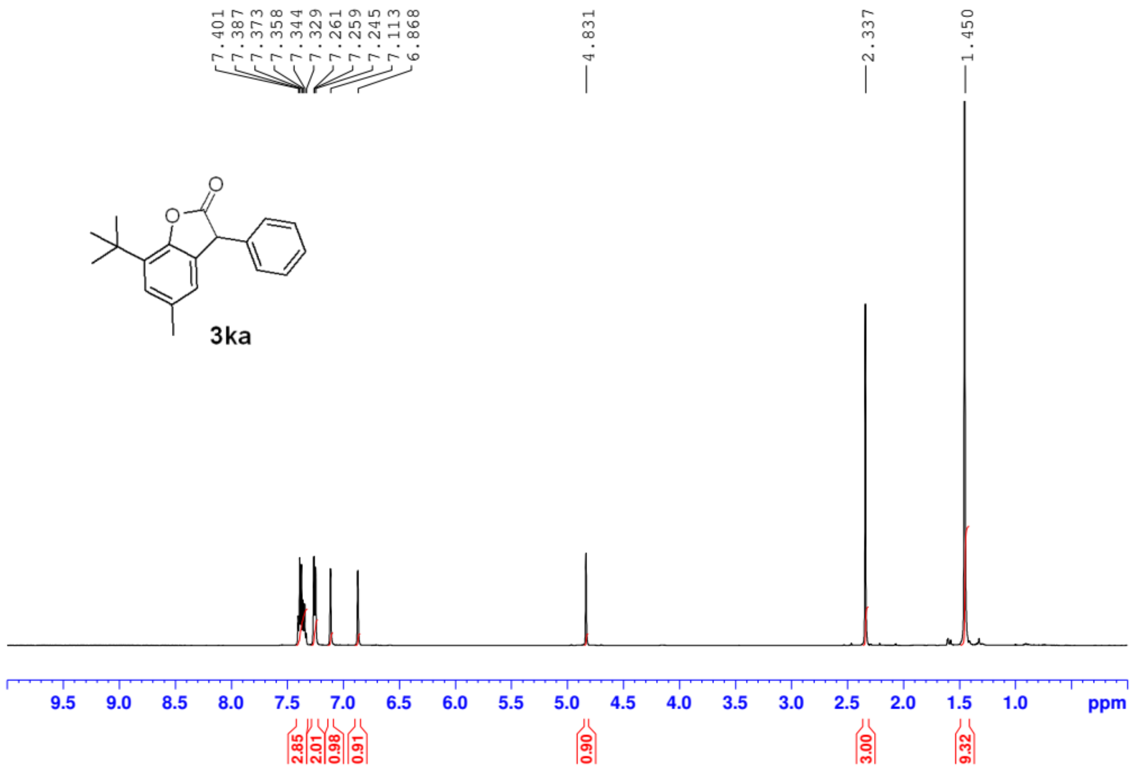

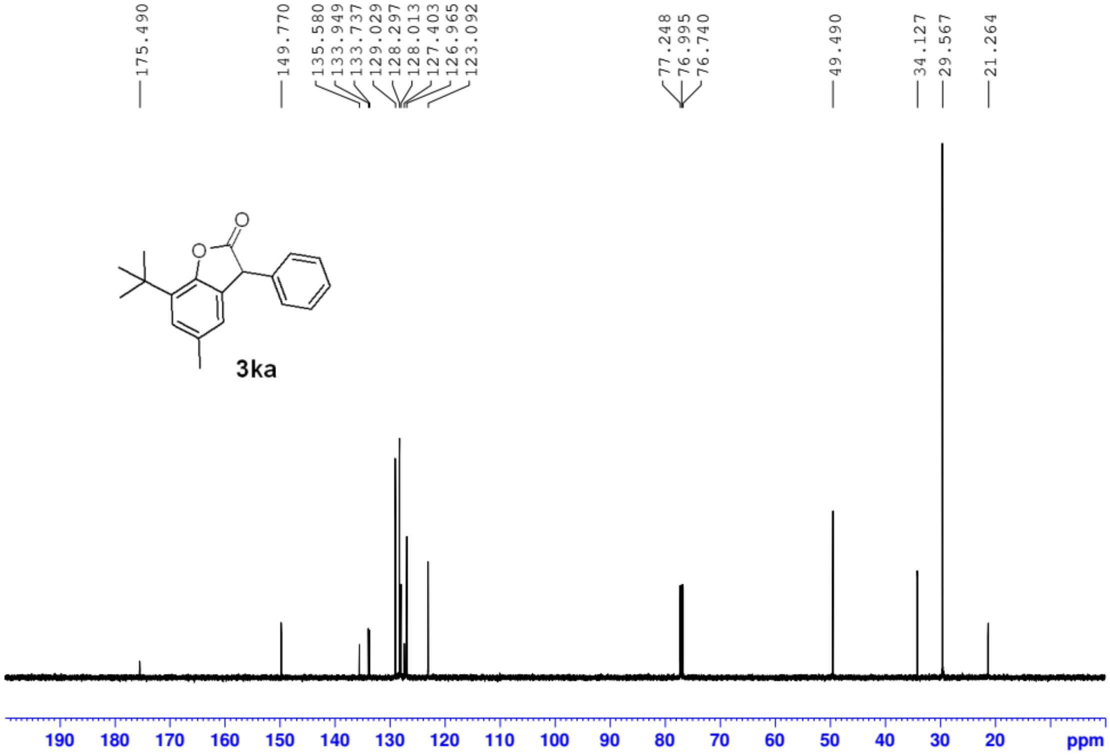

Figure S9. ^{1}H and ^{13}C NMR spectrum of **3ka**.

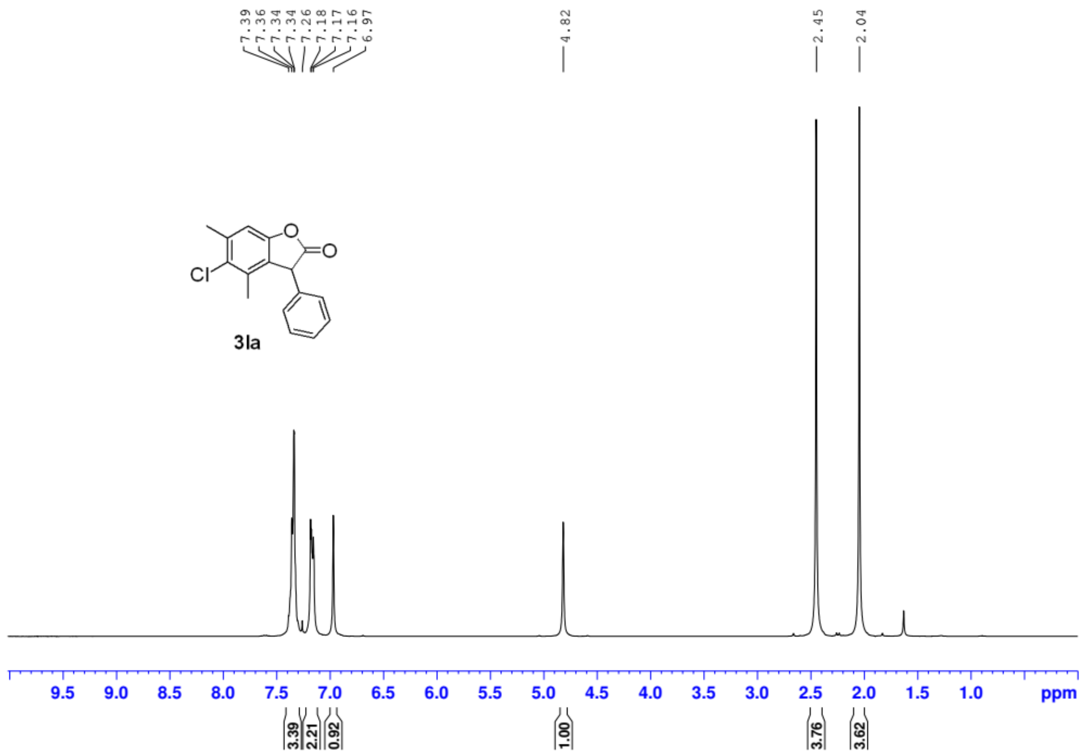

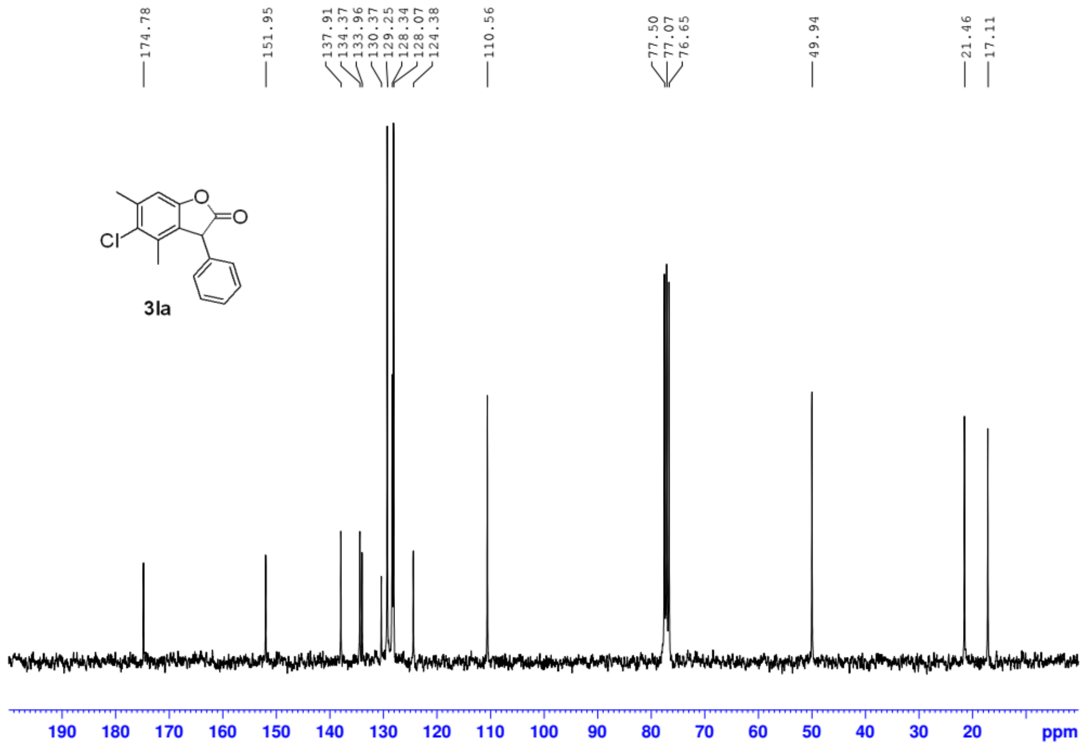

Figure S10. ^{1}H and ^{13}C NMR spectrum of **3la**.

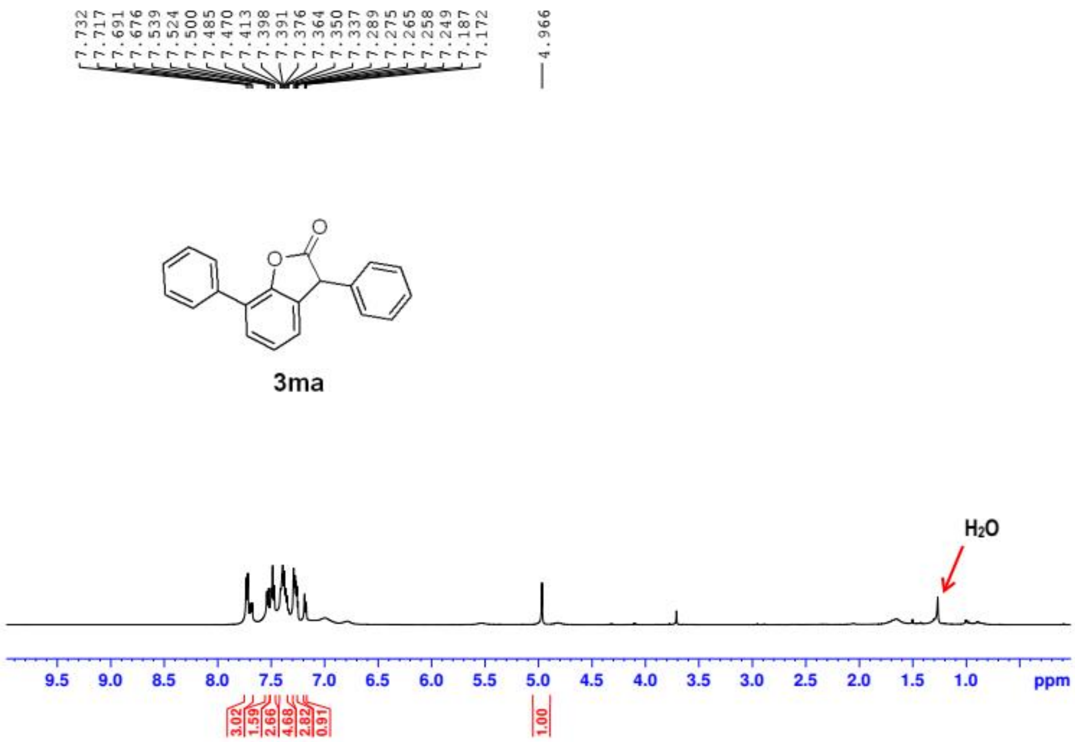

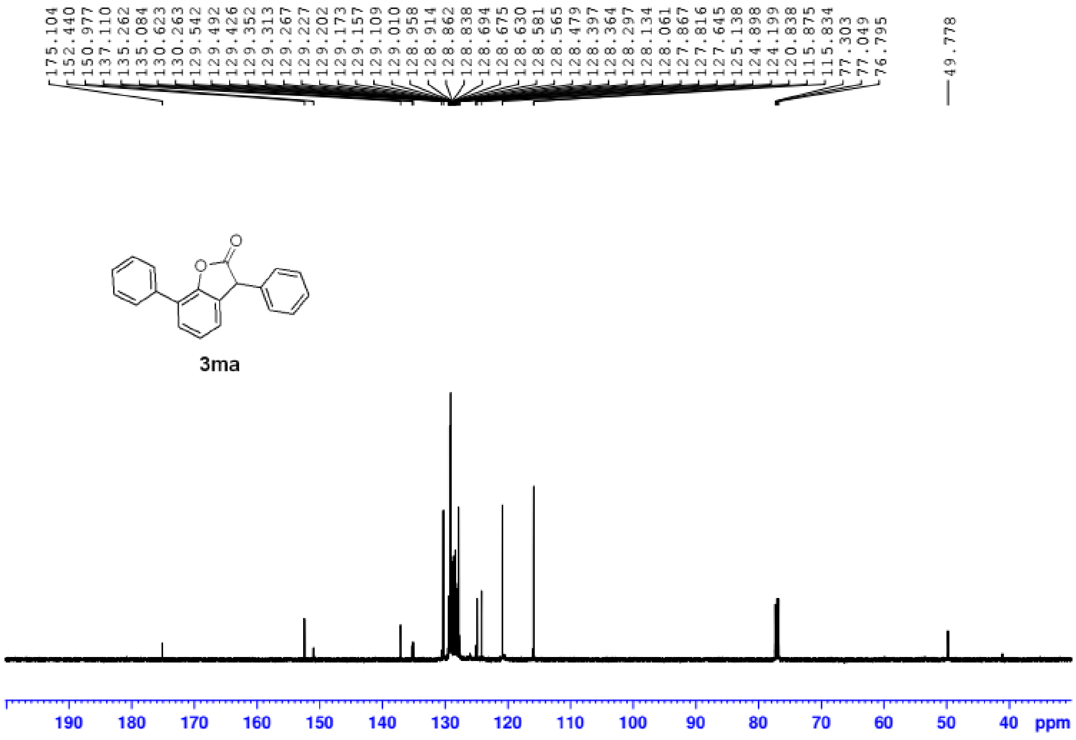

Figure S11. ^{1}H and ^{13}C NMR spectrum of **3ma**.

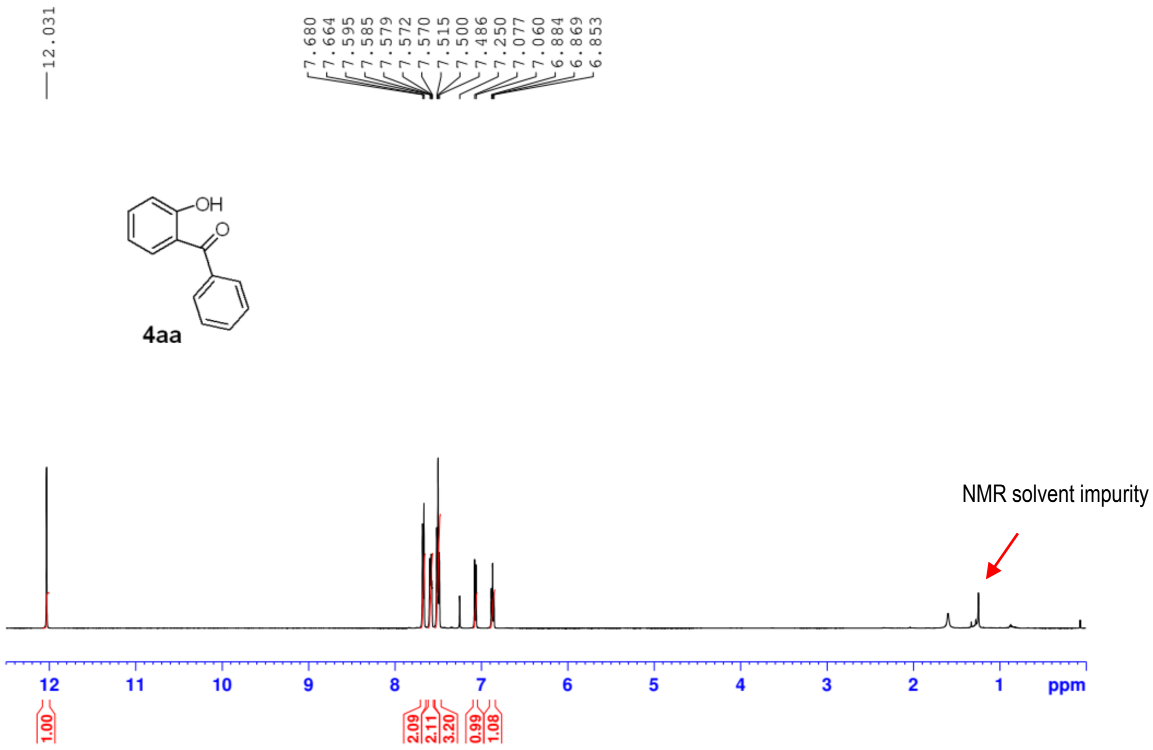

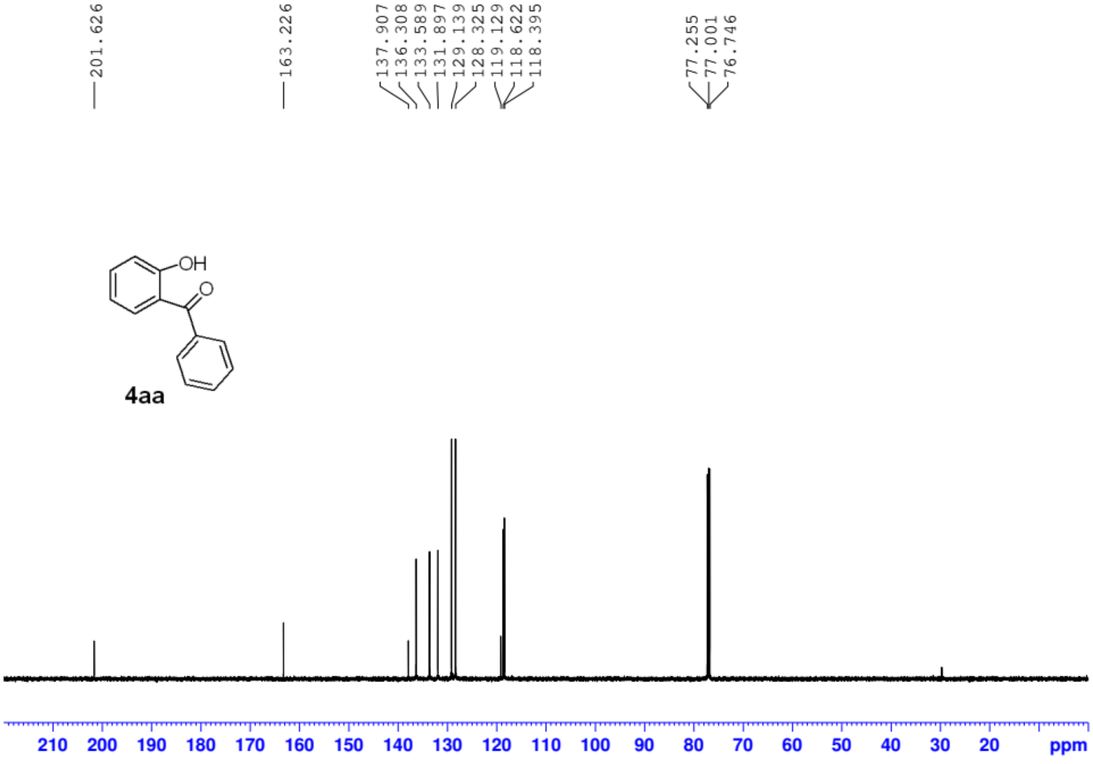

F

Figure S12. ^{1}H and ^{13}C NMR spectrum of **4aa**.

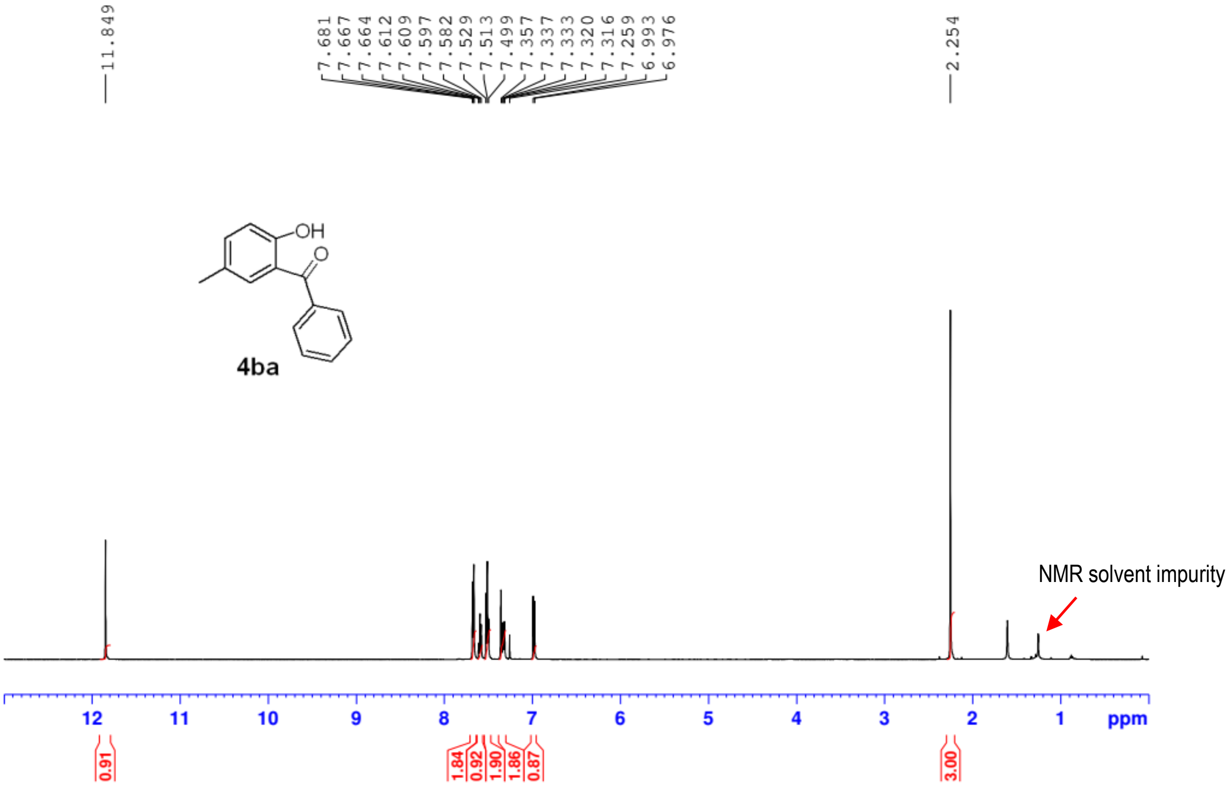

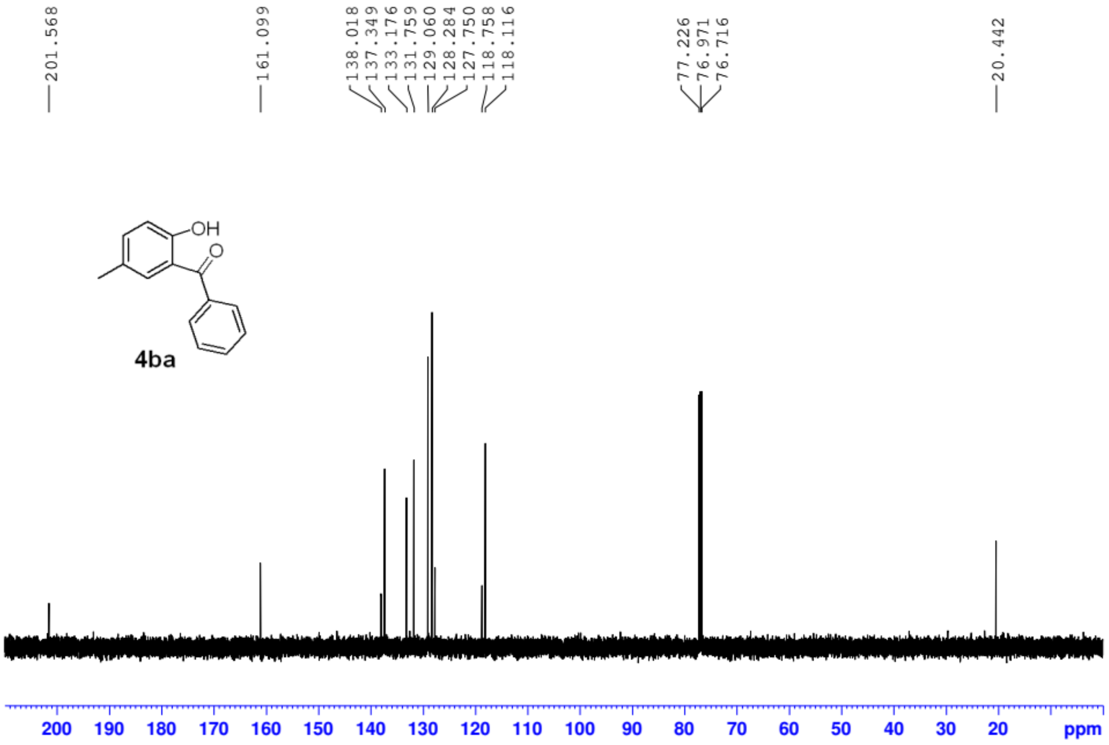

Figure S13. ^{1}H and ^{13}C NMR spectrum of **4ba**.

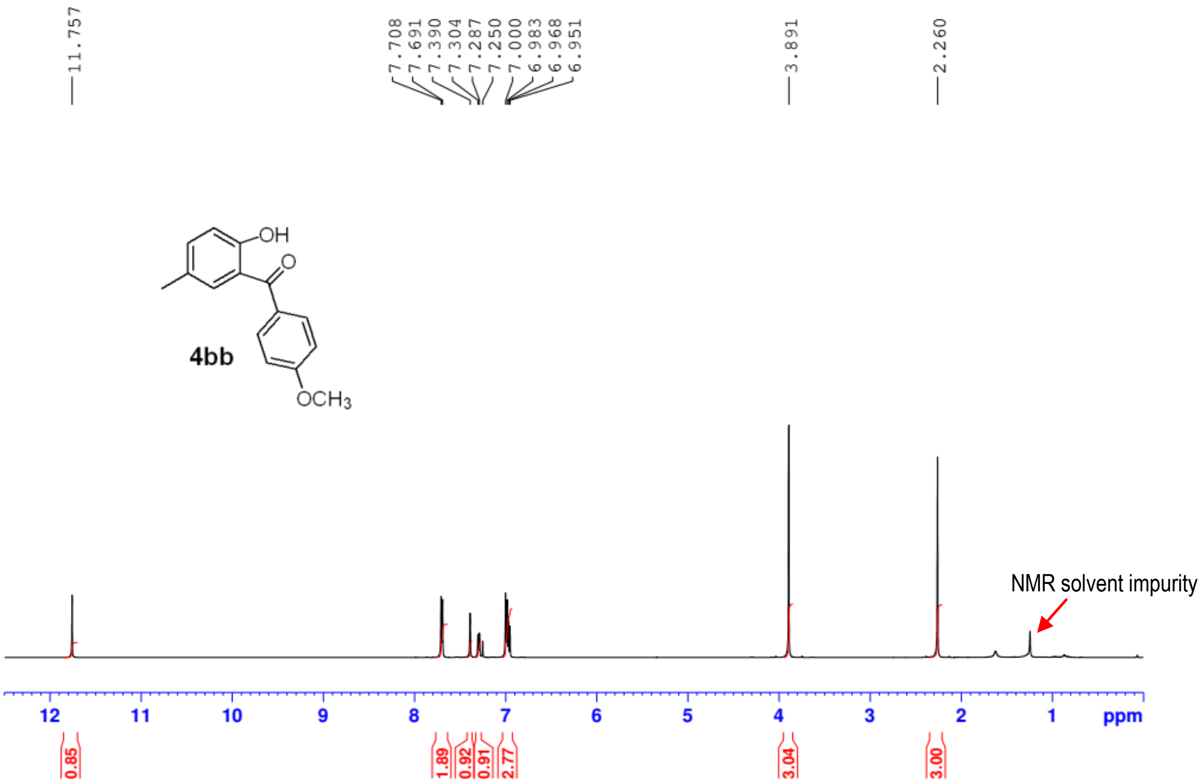

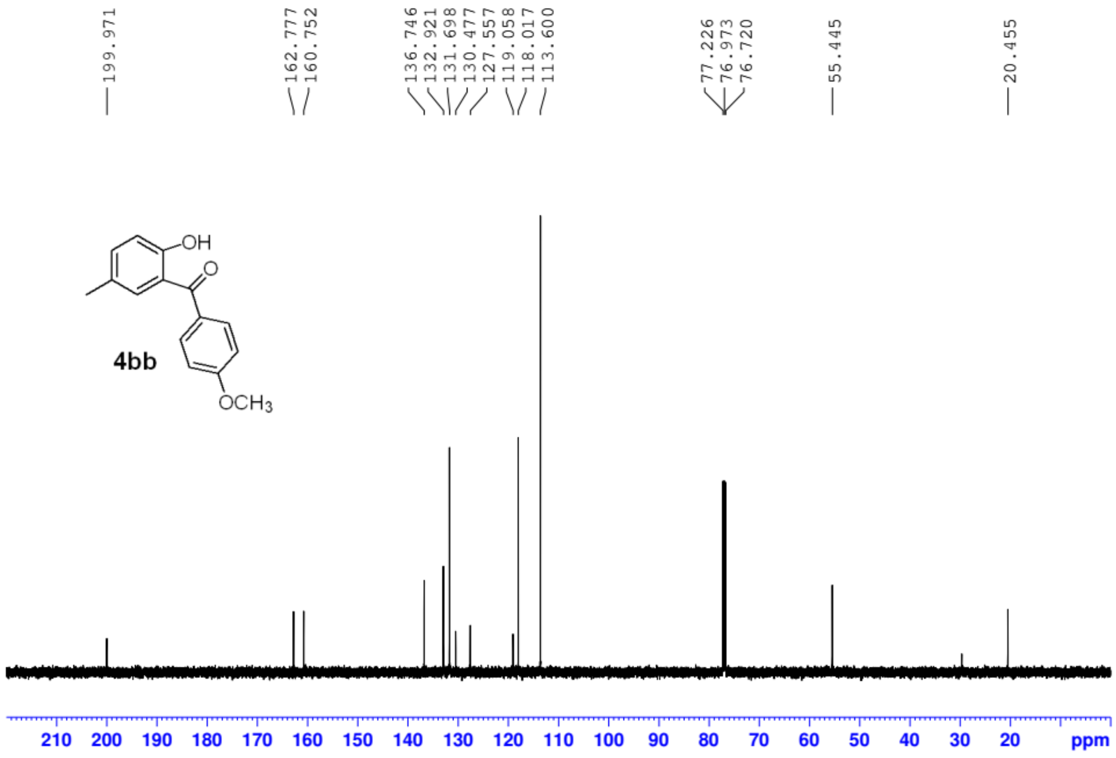

Figure S14. ^{1}H and ^{13}C NMR spectrum of **4bb**.

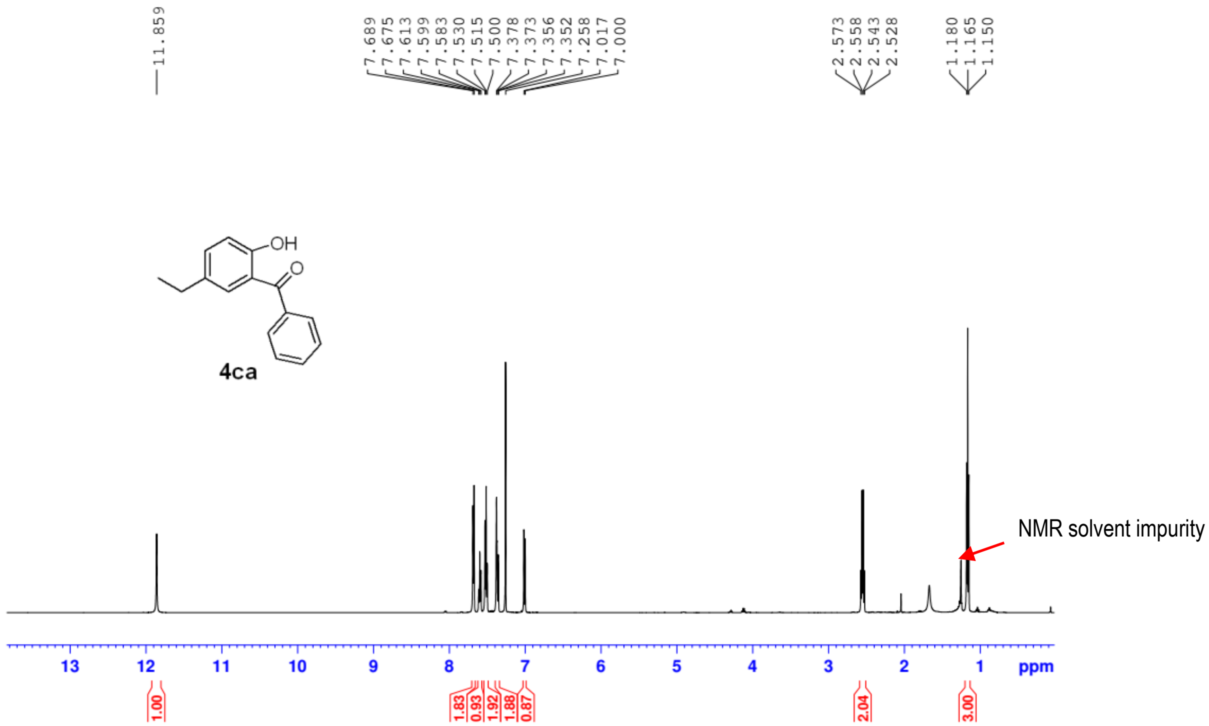

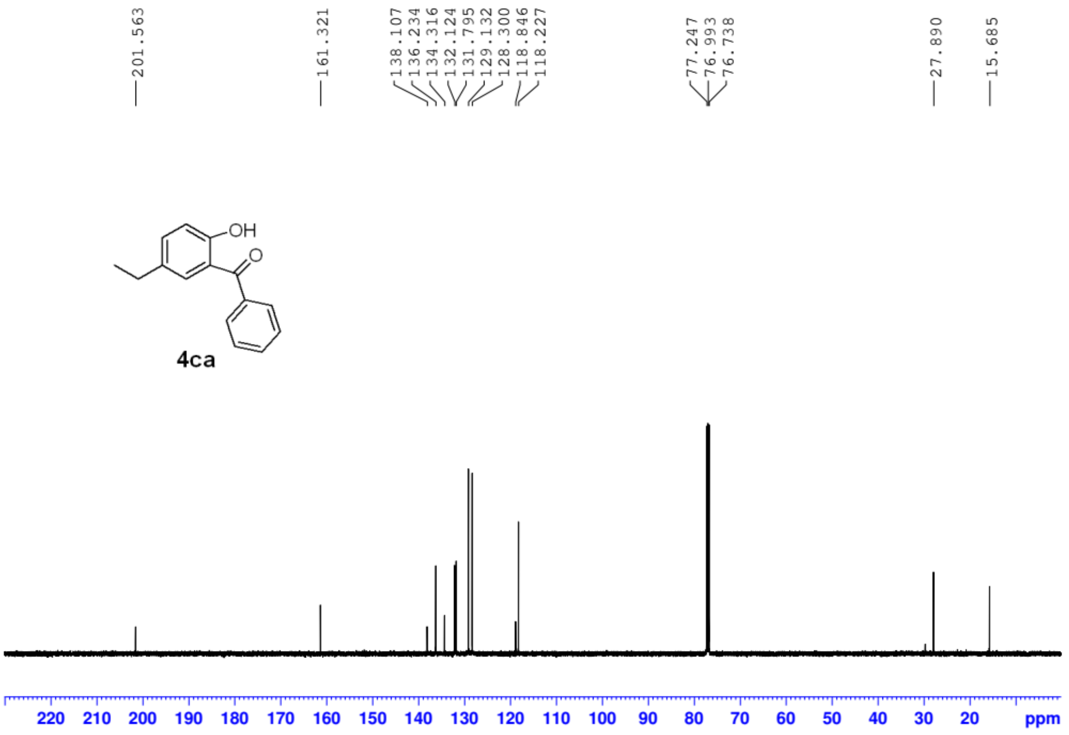

Figure S15. ^{1}H and ^{13}C NMR spectrum of **4ca**.

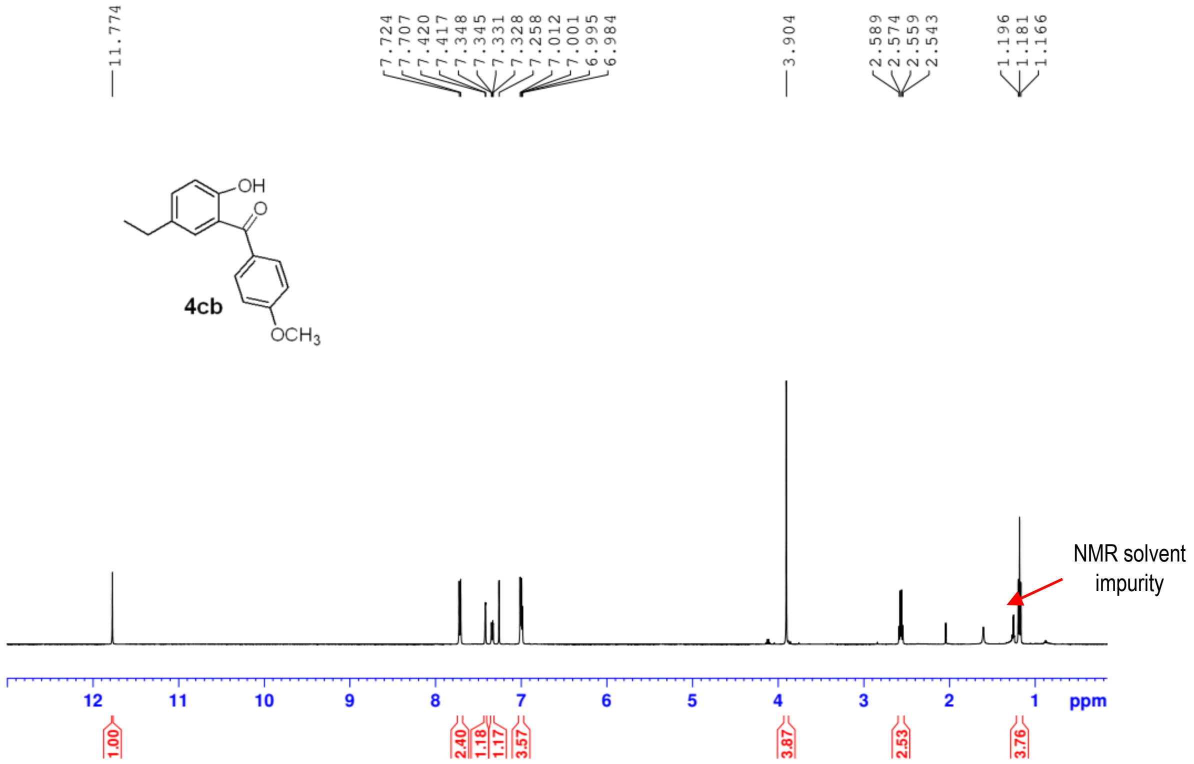

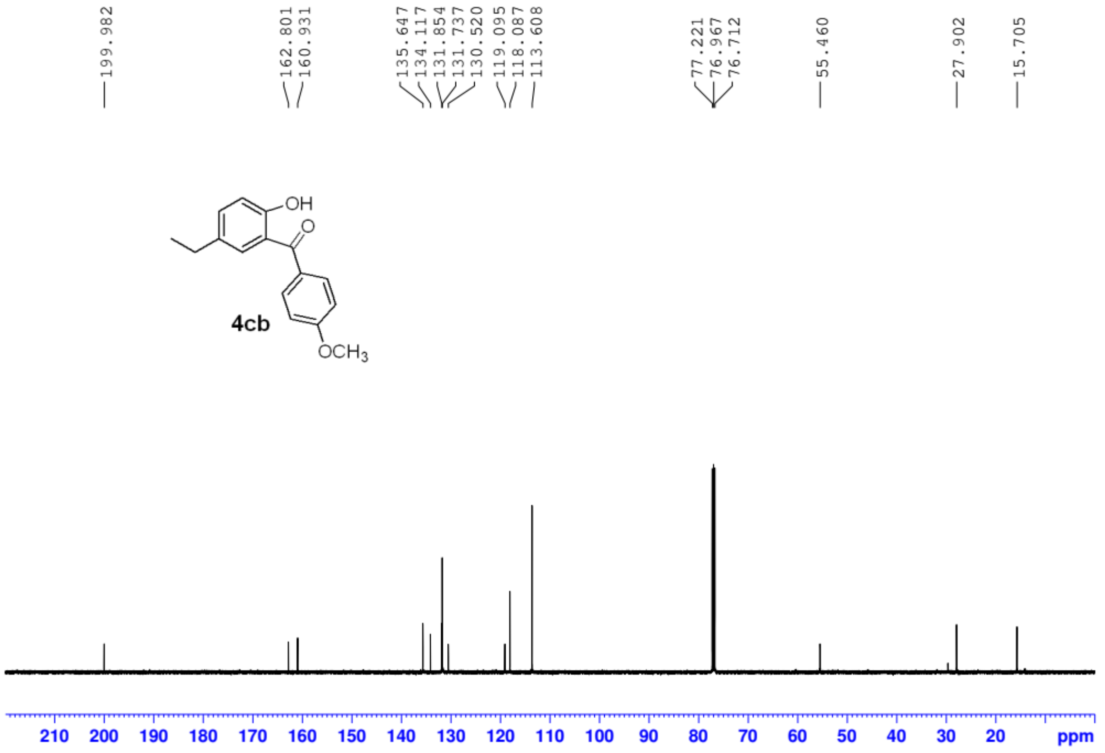

Figure S16. ^{1}H and ^{13}C NMR spectrum of **4cb**

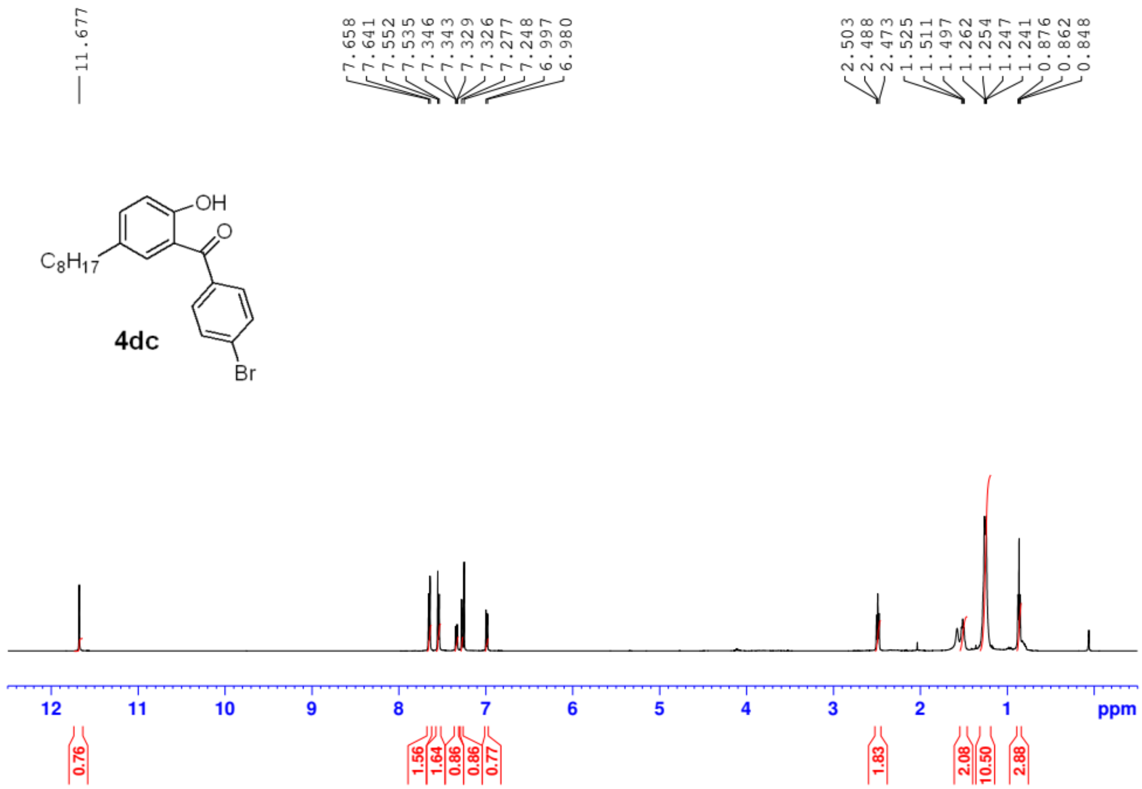

.

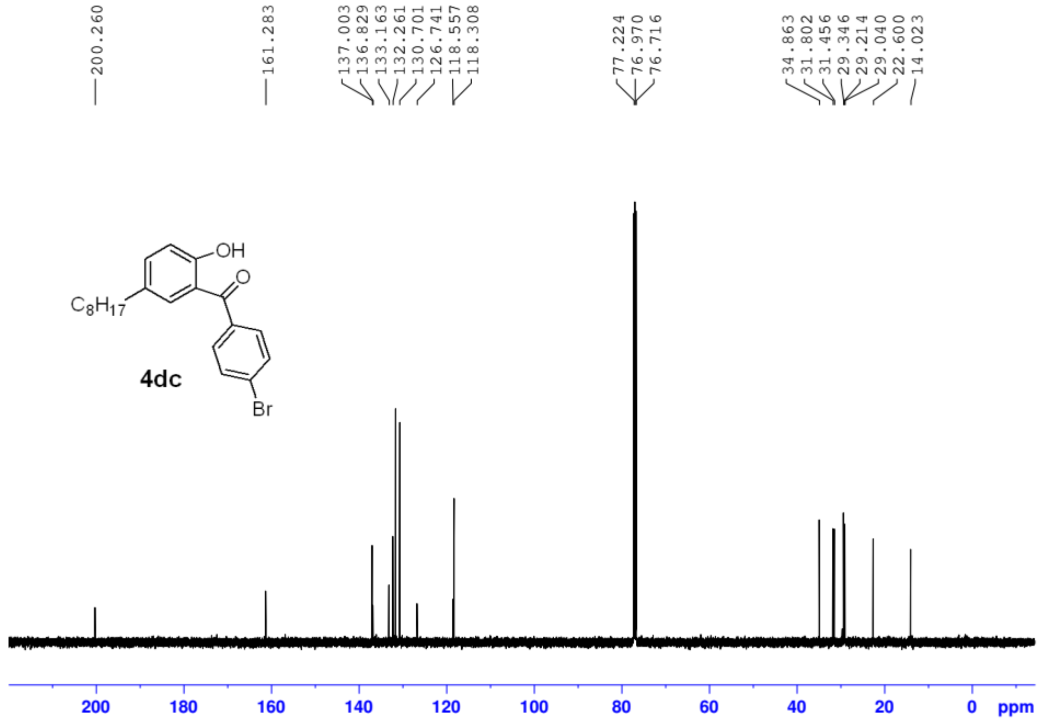

Figure S17. ^{1}H and ^{13}C NMR spectrum of **4dc**.

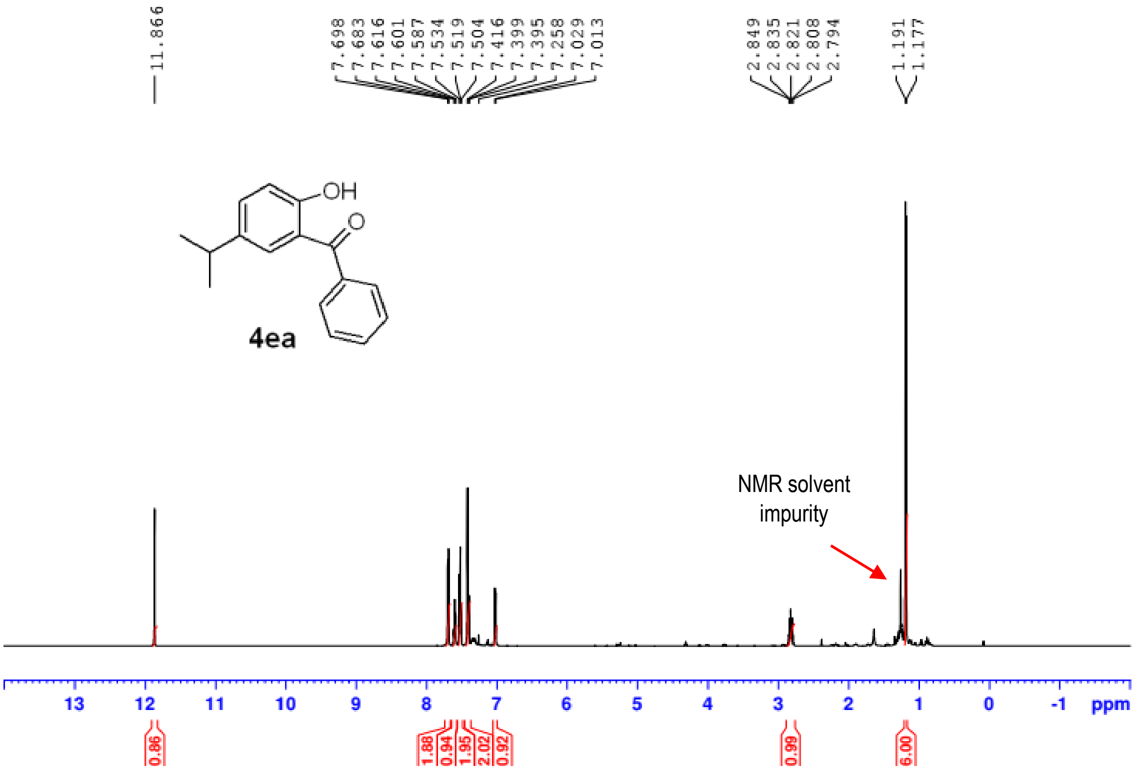

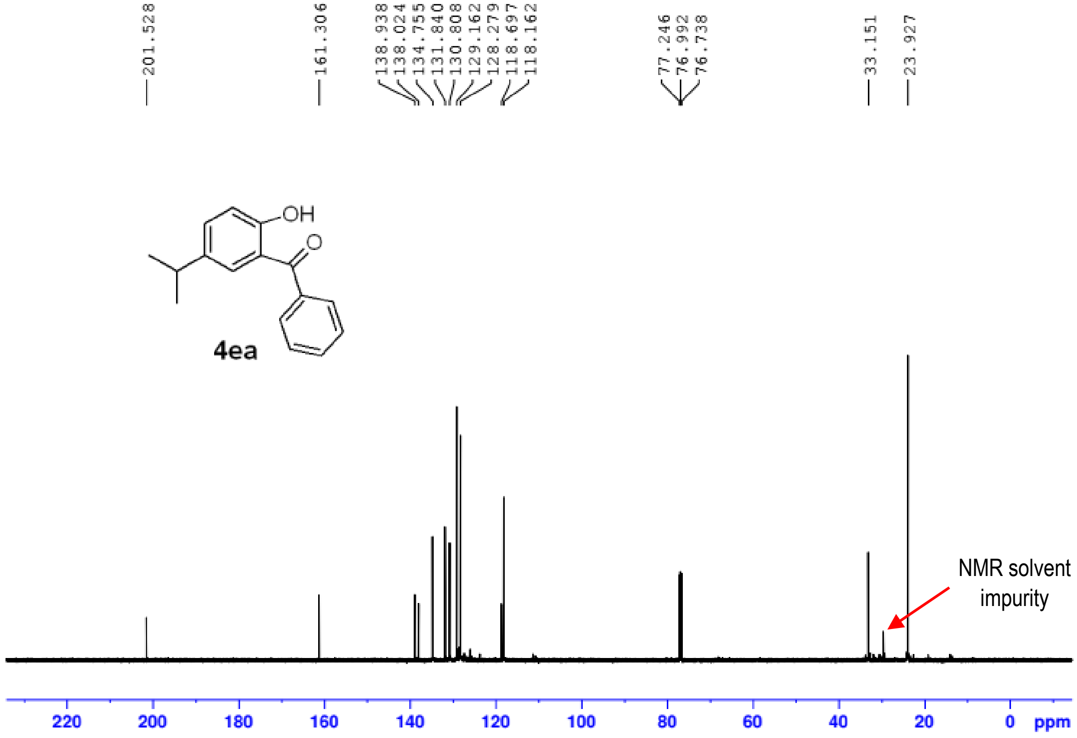

Figure S18. ^{1}H and ^{13}C NMR spectrum of **4ea**.

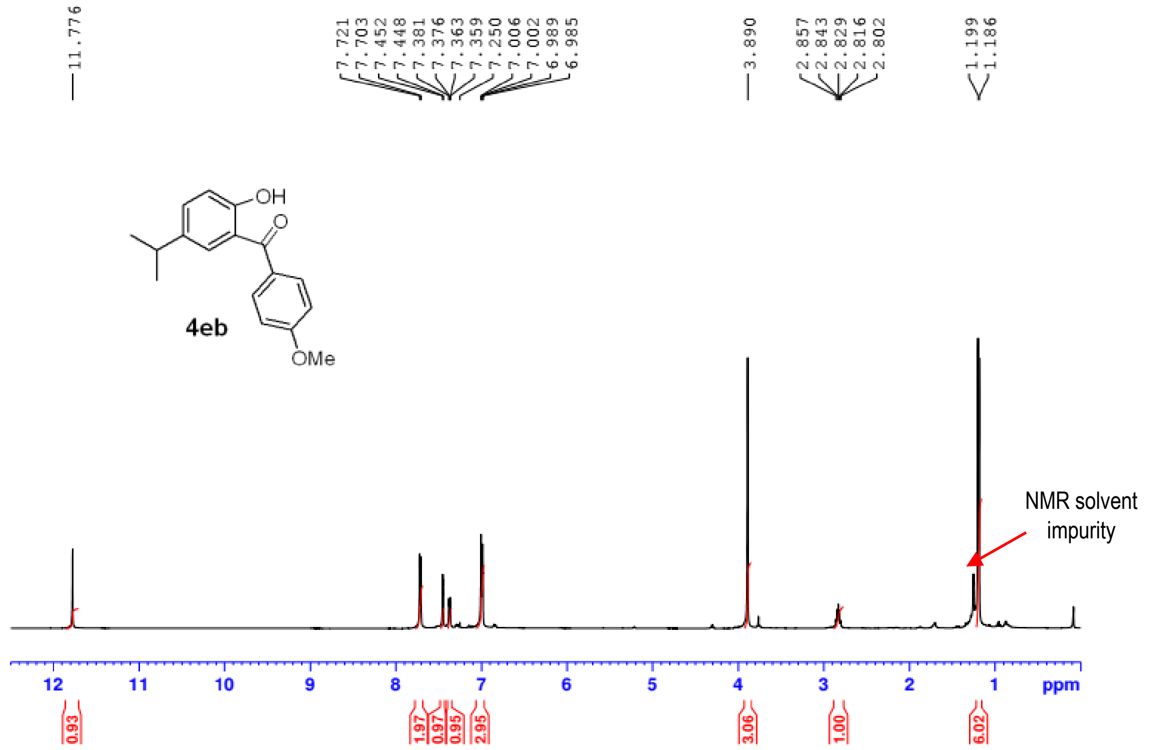

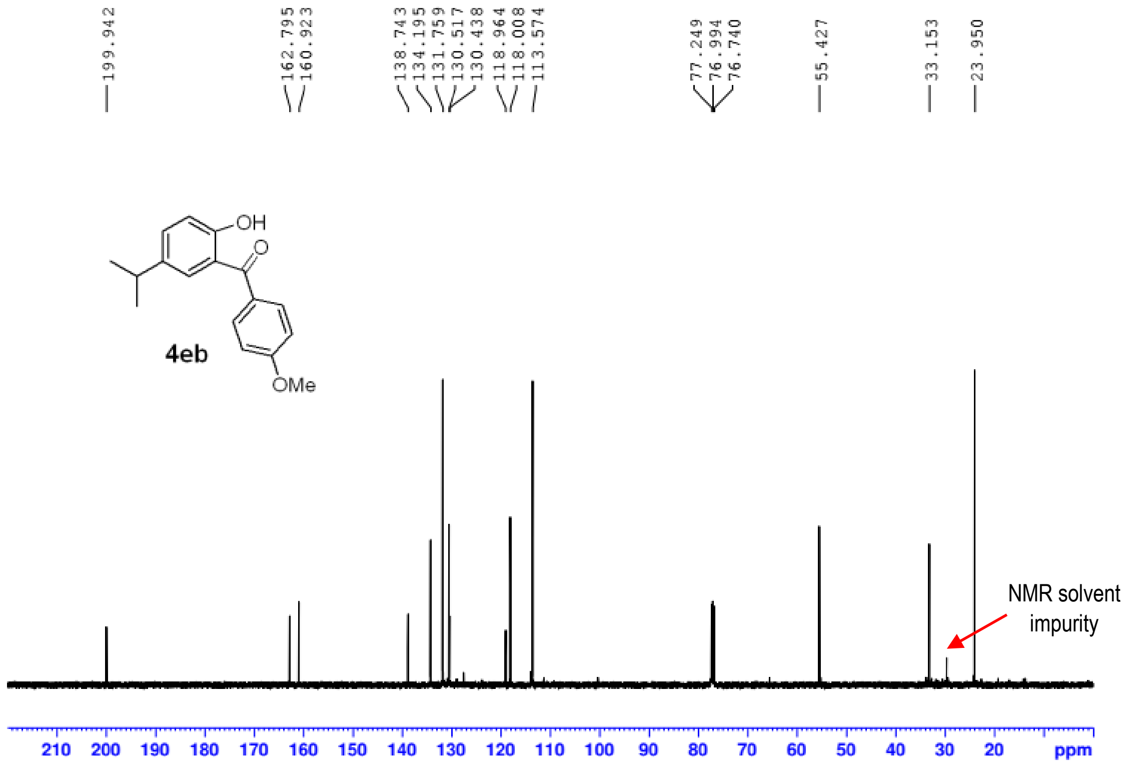

Figure S19. ^{1}H and ^{13}C NMR spectrum of **4eb**.

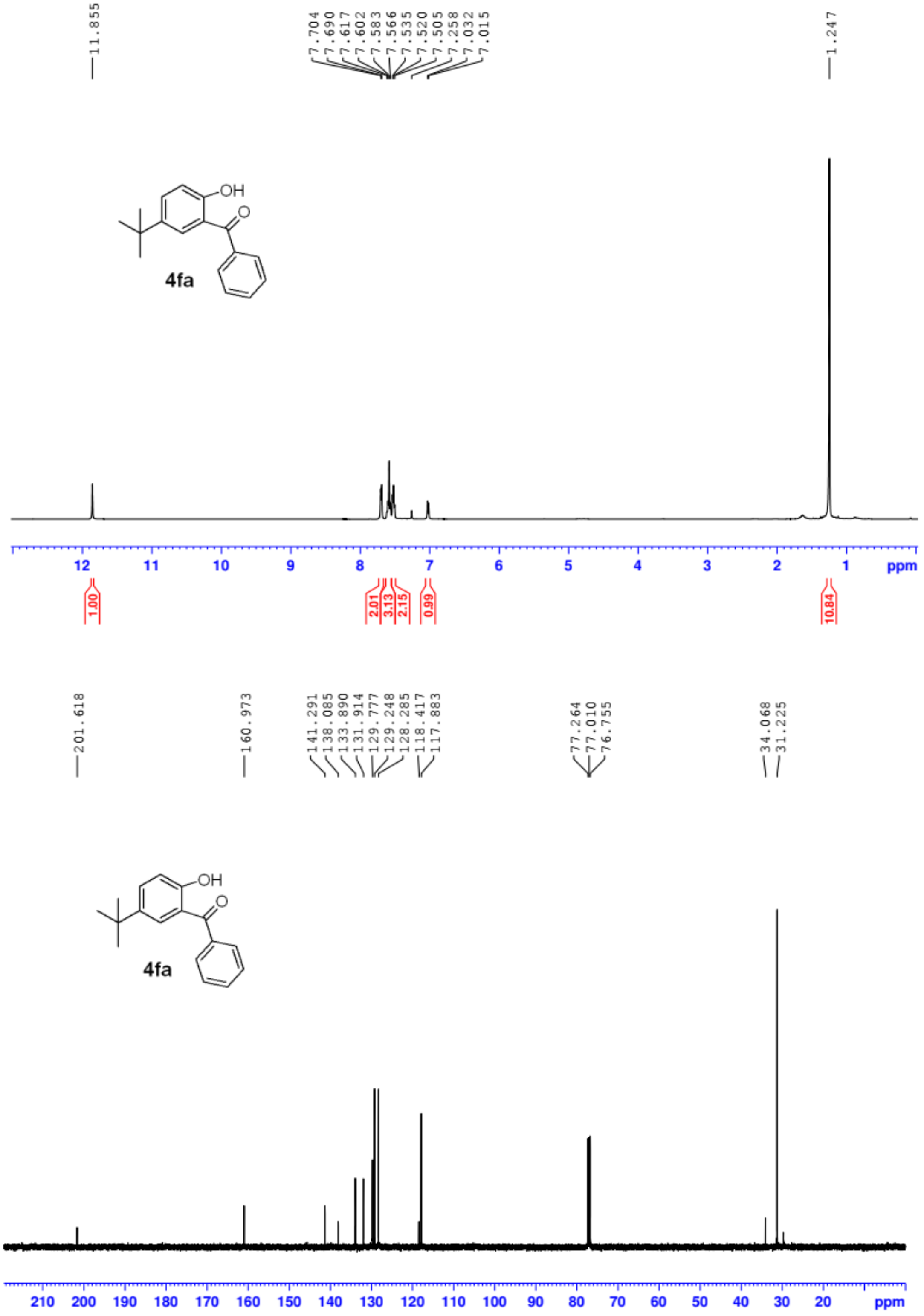

Figure S20. ^{1}H and ^{13}C NMR spectrum of **4fa**.

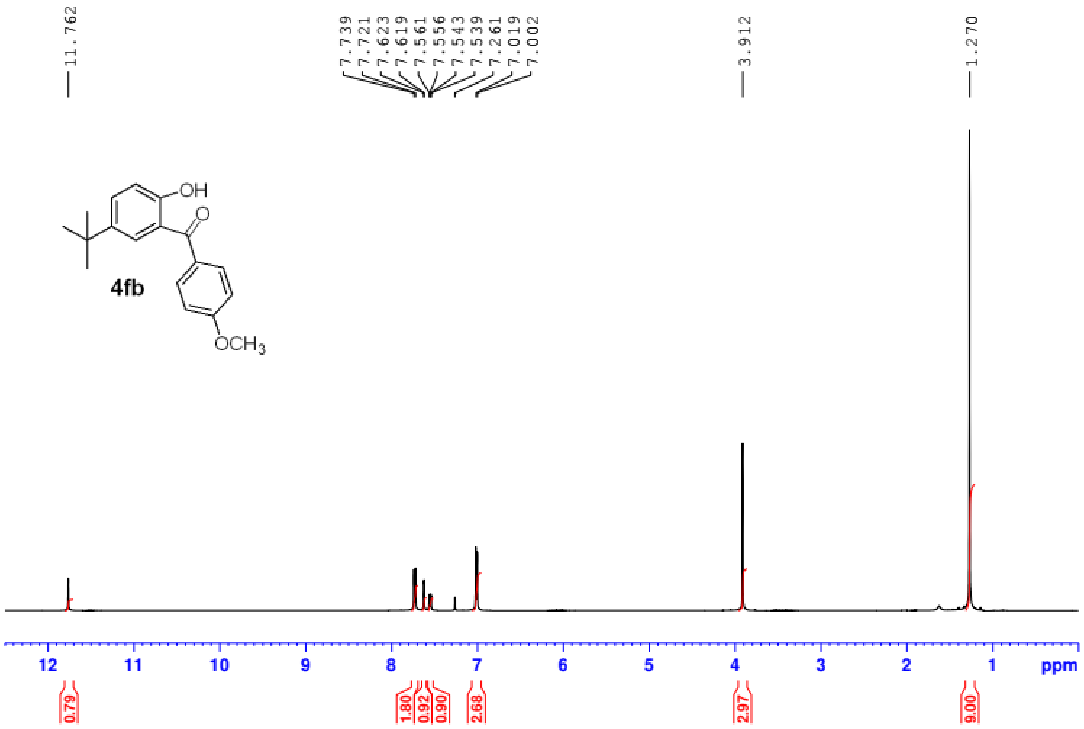

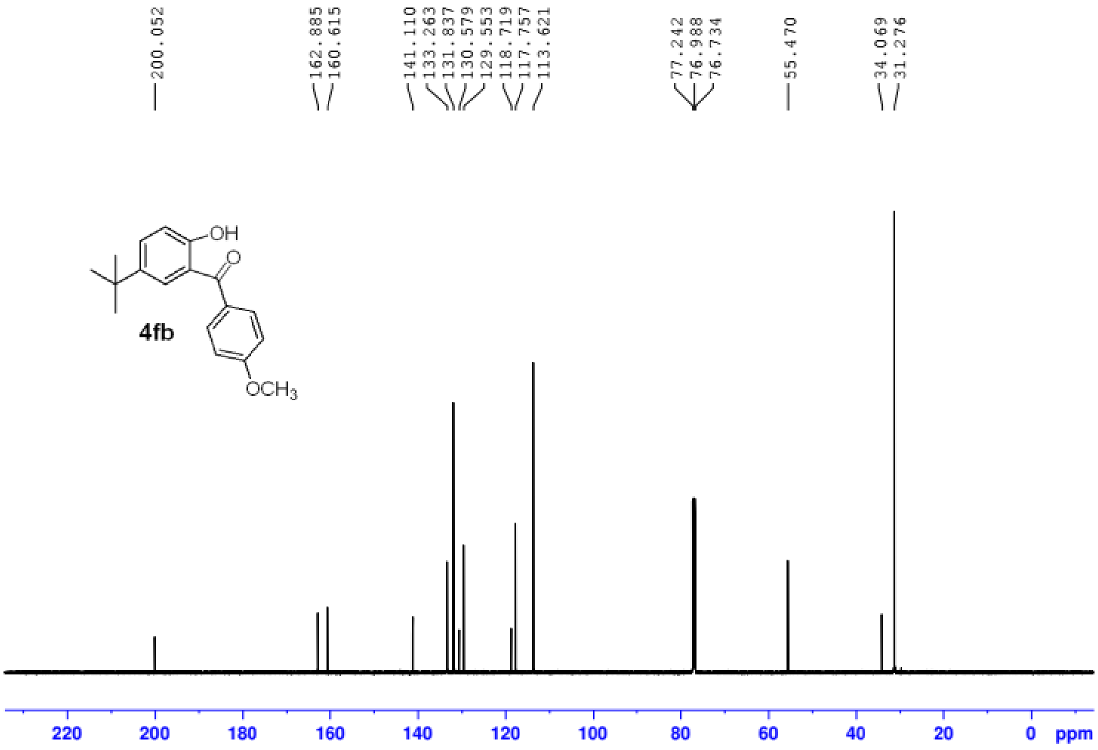

Figure S21. ^{1}H and ^{13}C NMR spectrum of **4fb**.

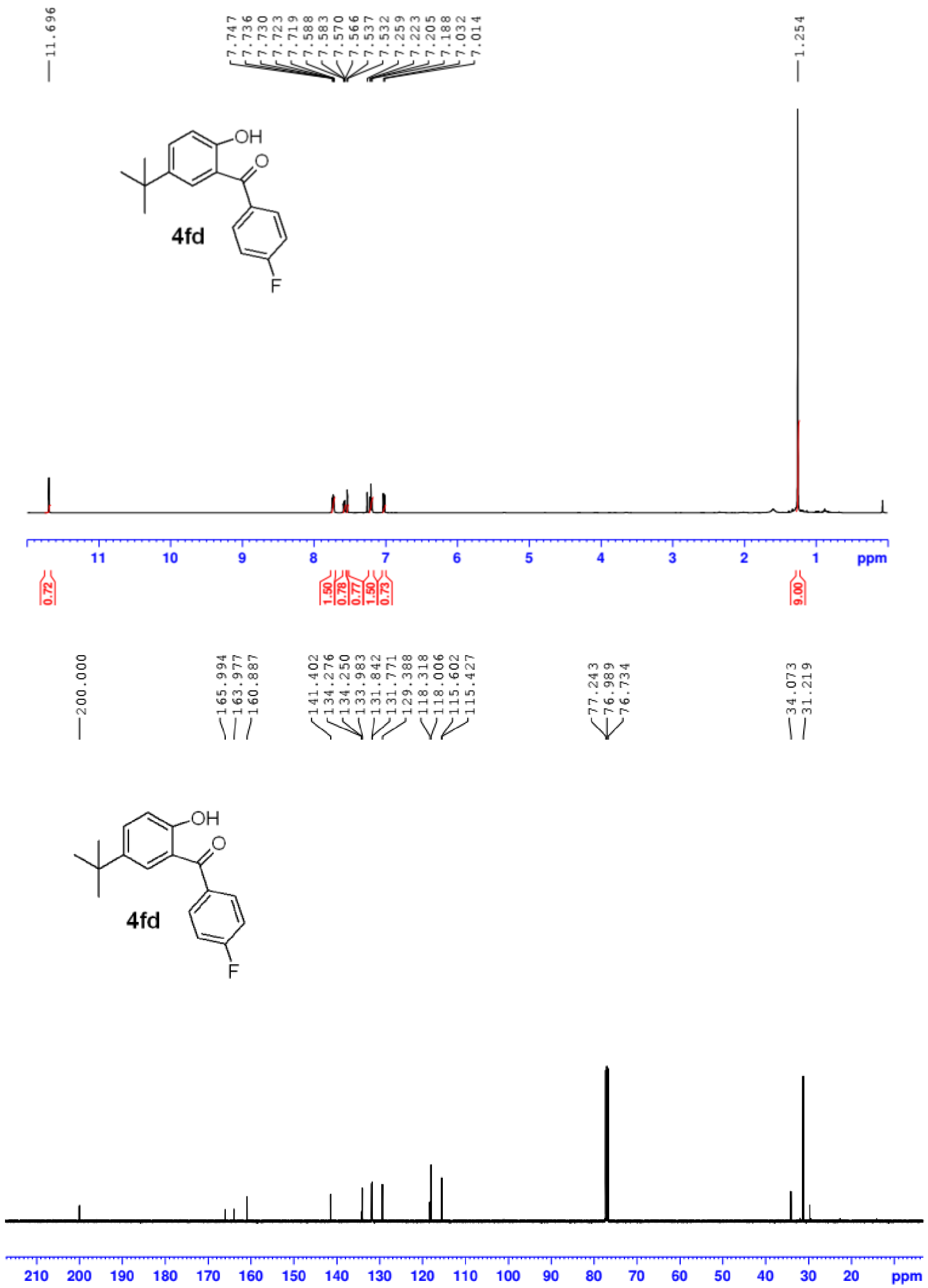

Figure S22. ^{1}H and ^{13}C NMR spectrum of **4fd**.

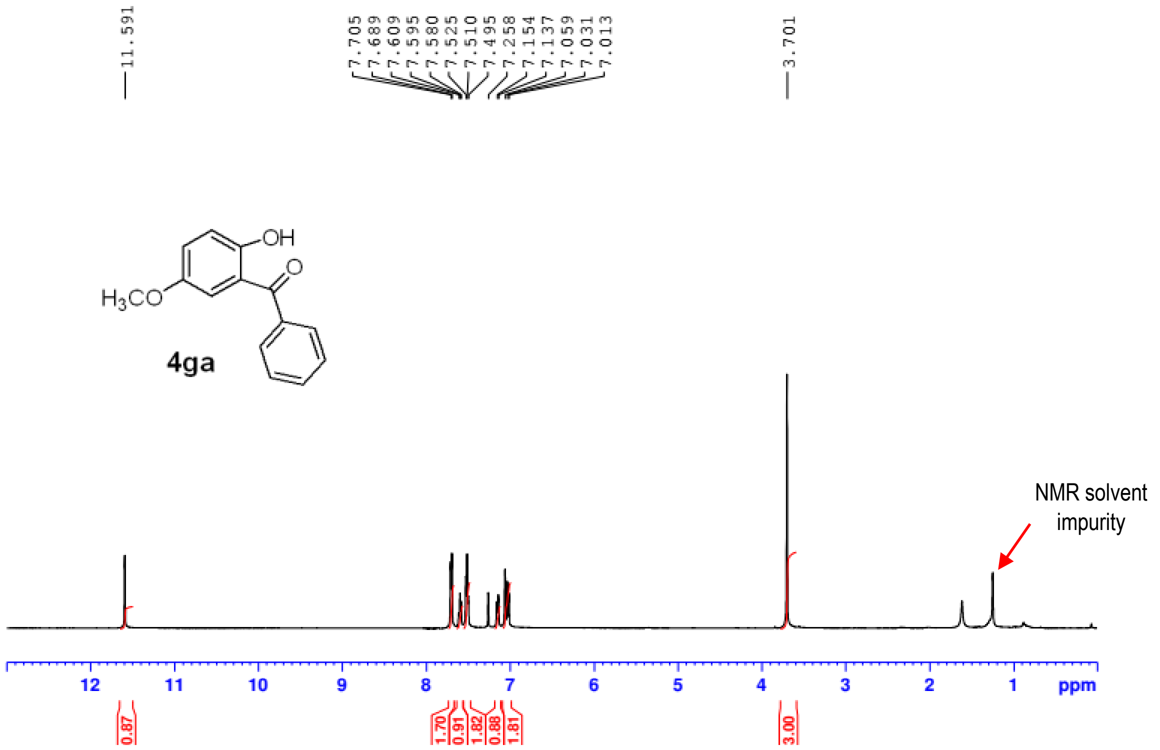

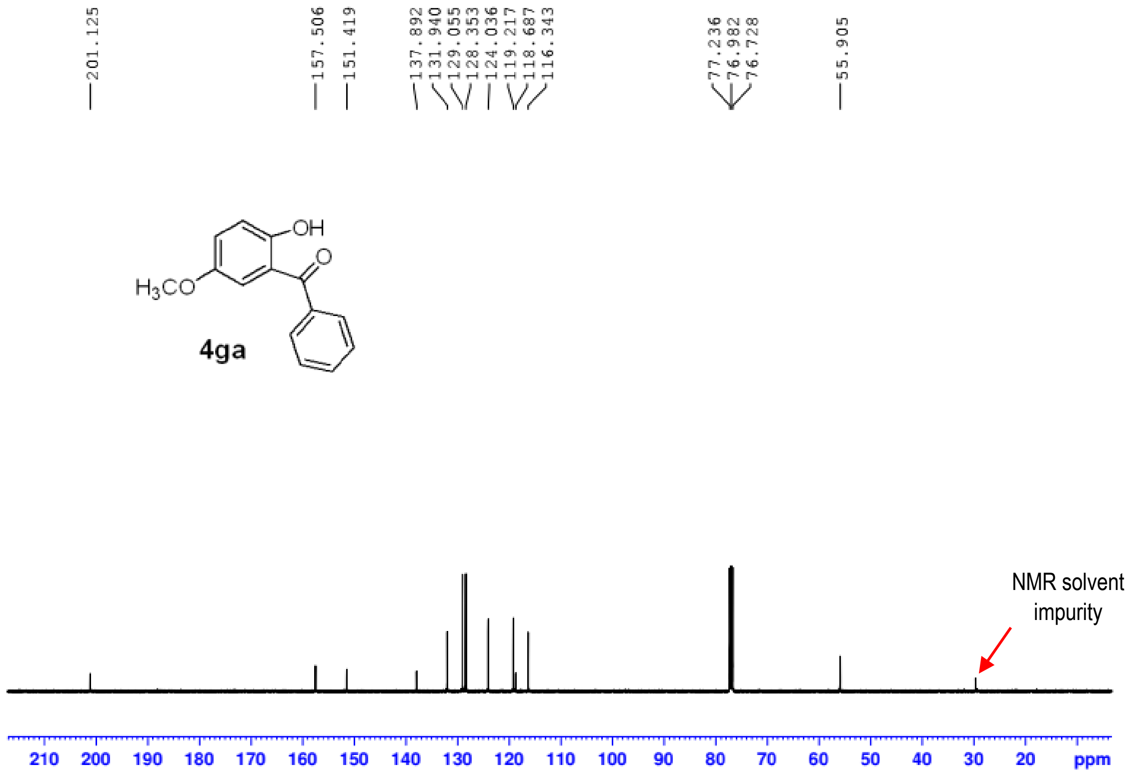

Figure S23. ^{1}H and ^{13}C NMR spectrum of **4ga**.

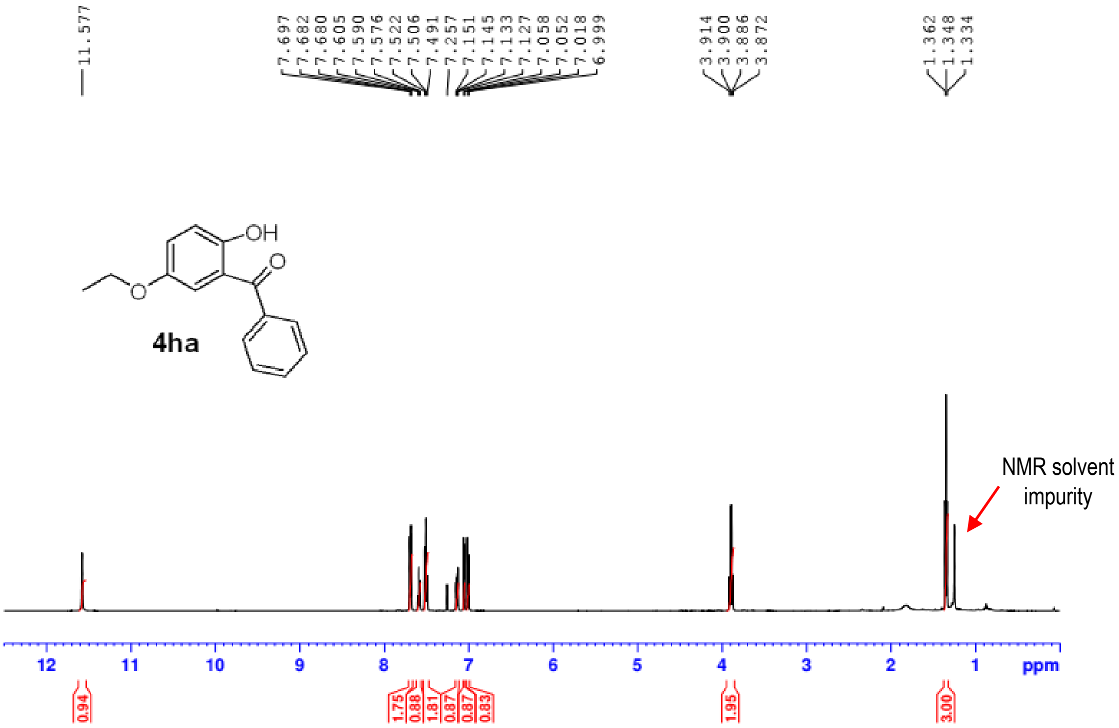

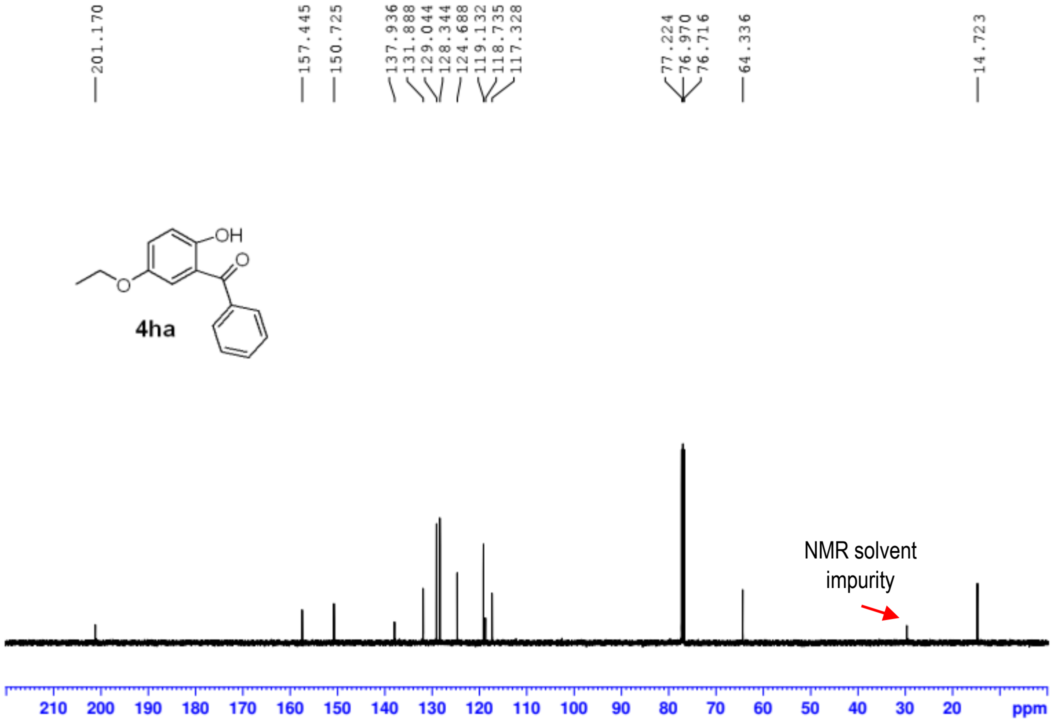

Figure S24. ^{1}H and ^{13}C NMR spectrum of **4ha**.

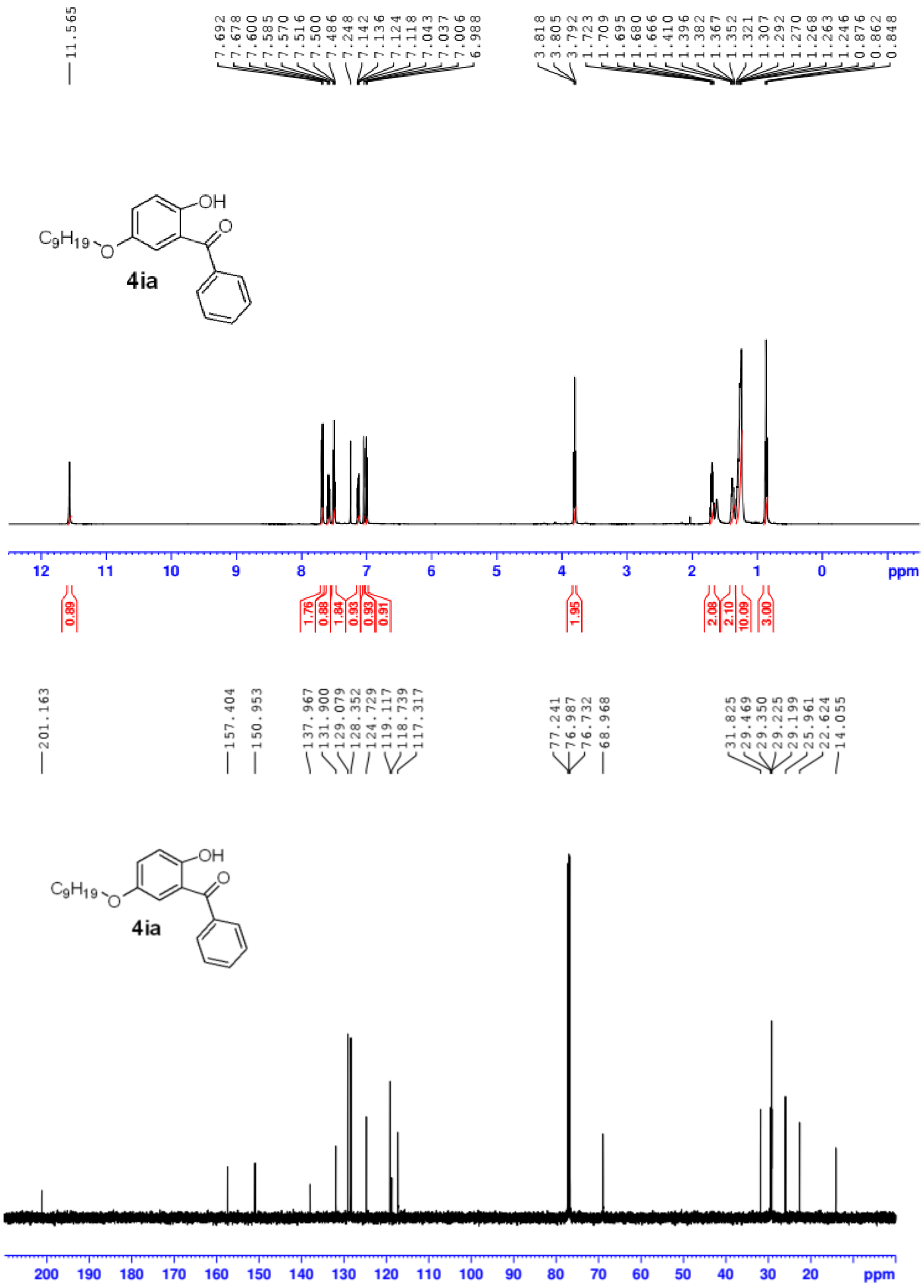

Figure S25. ^{1}H and ^{13}C NMR spectrum of **4ia**.

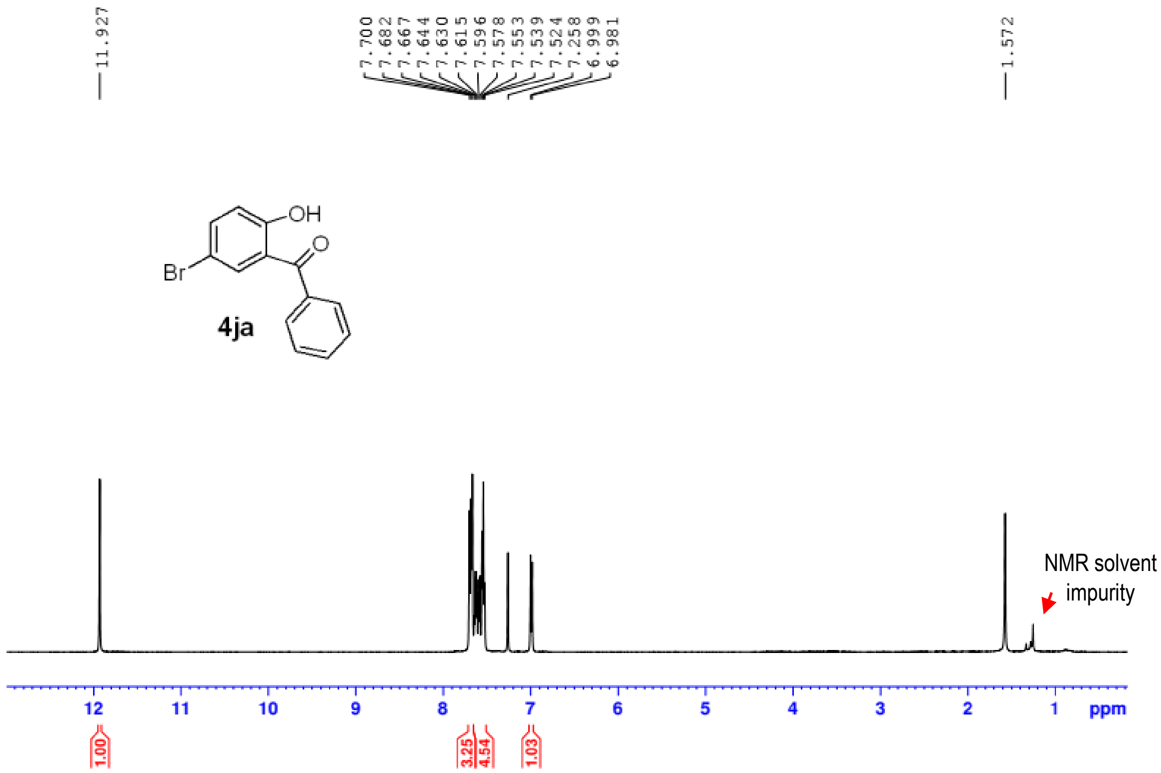

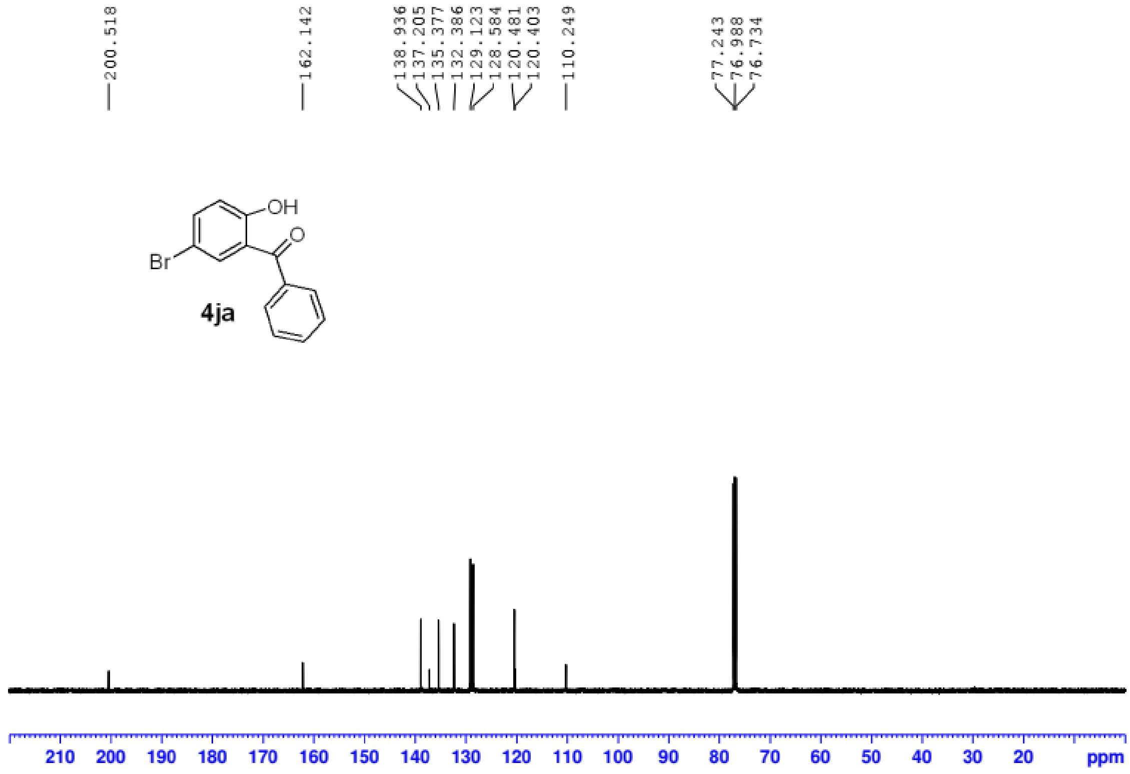

Figure S26. ^{1}H and ^{13}C NMR spectrum of **4ja**.

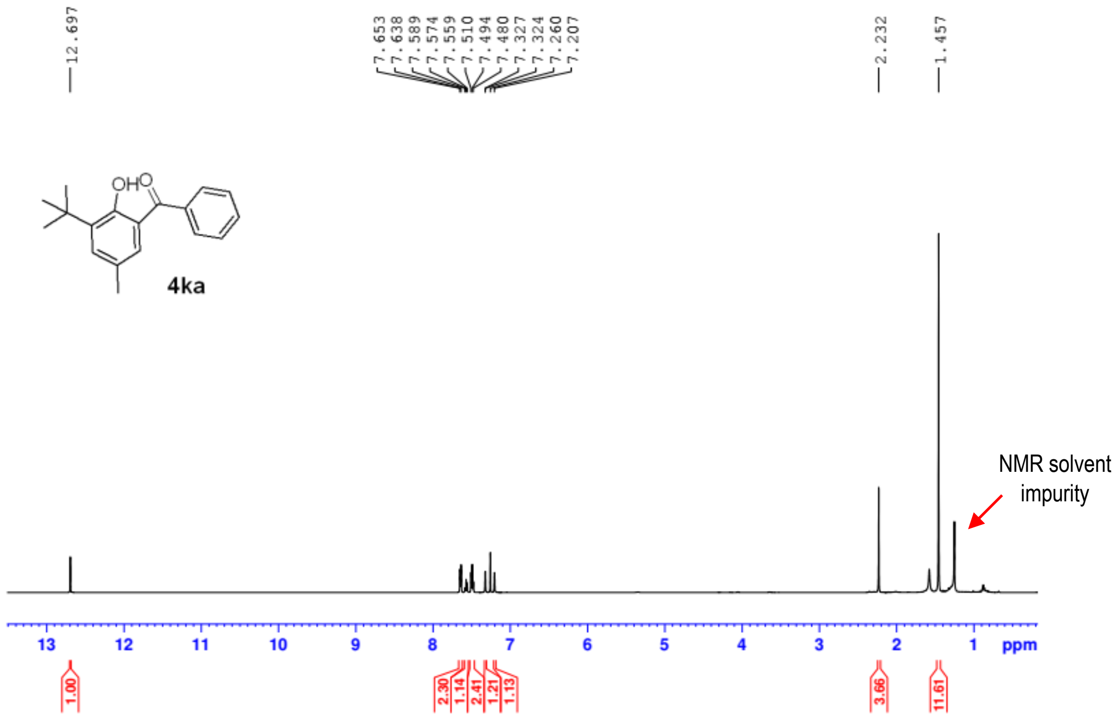

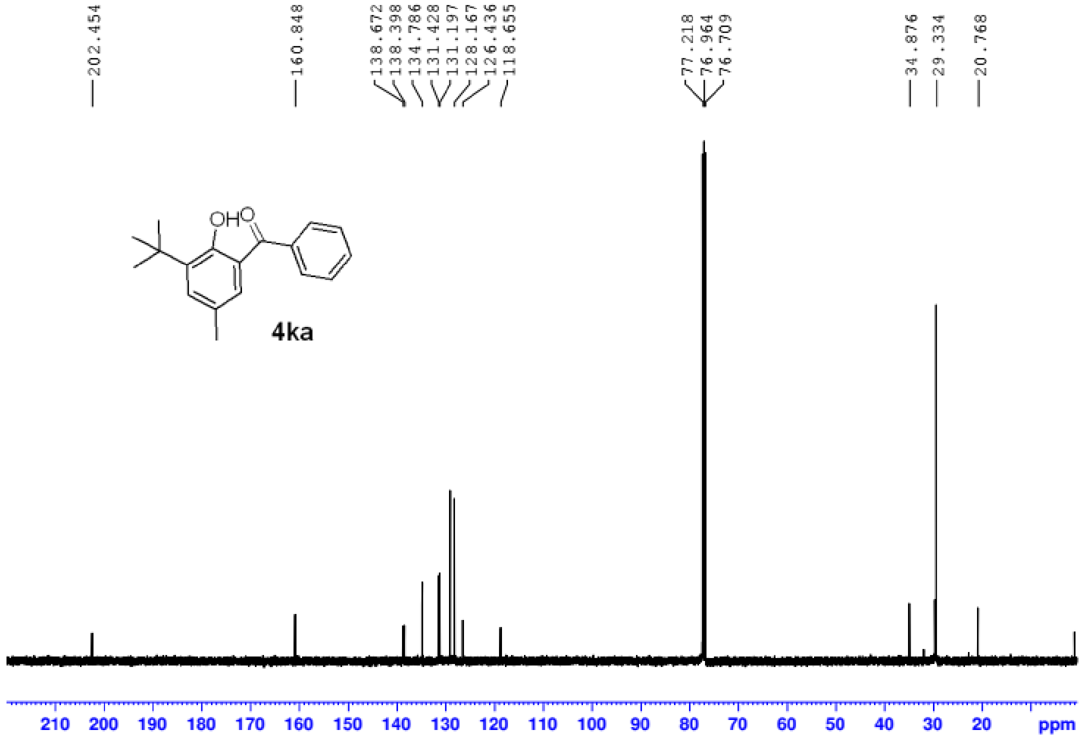

Figure S27. ^{1}H and ^{13}C NMR spectrum of **4ka**.

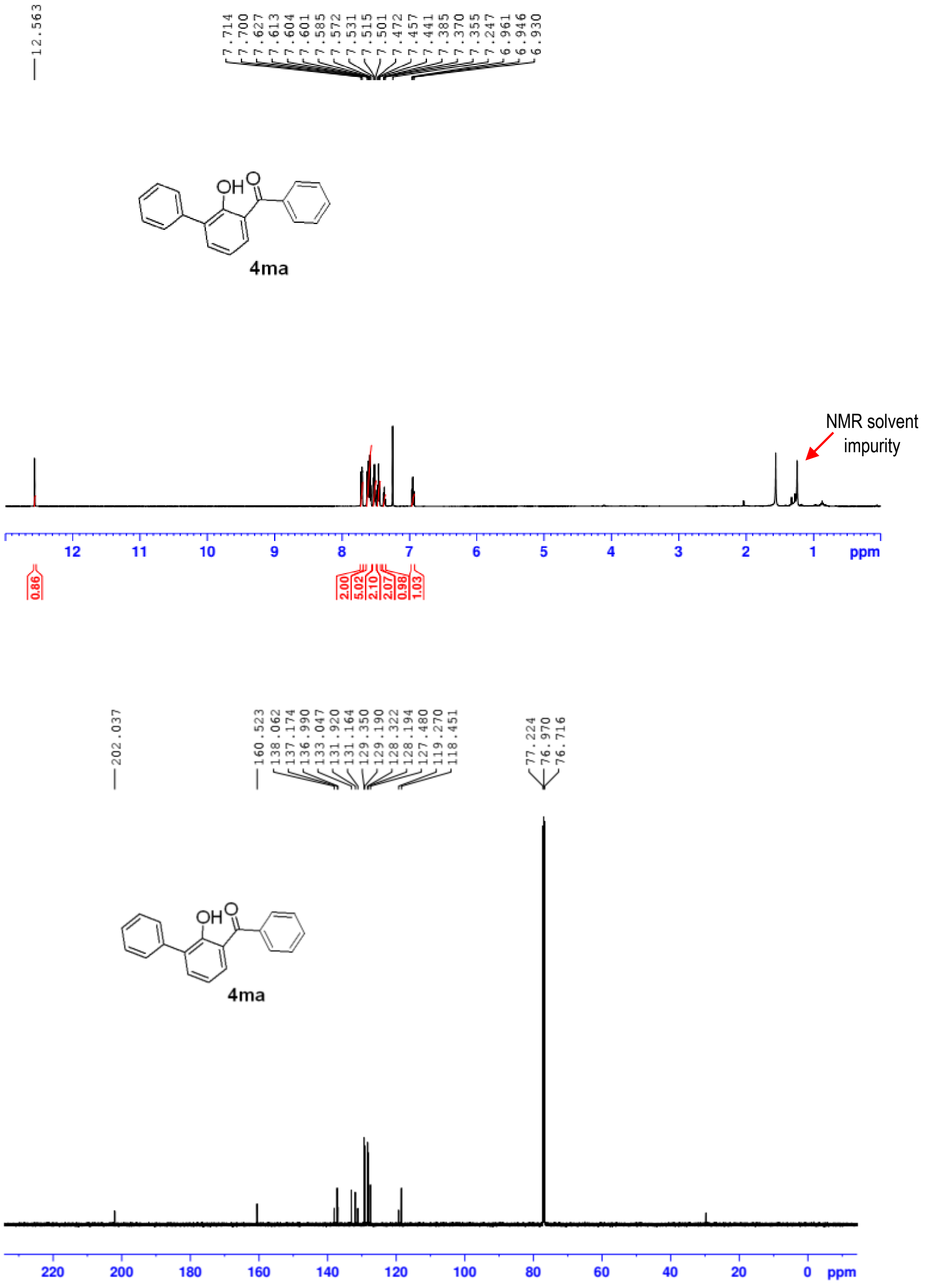

Figure S28. ^{1}H and ^{13}C NMR spectrum of **4ma**.

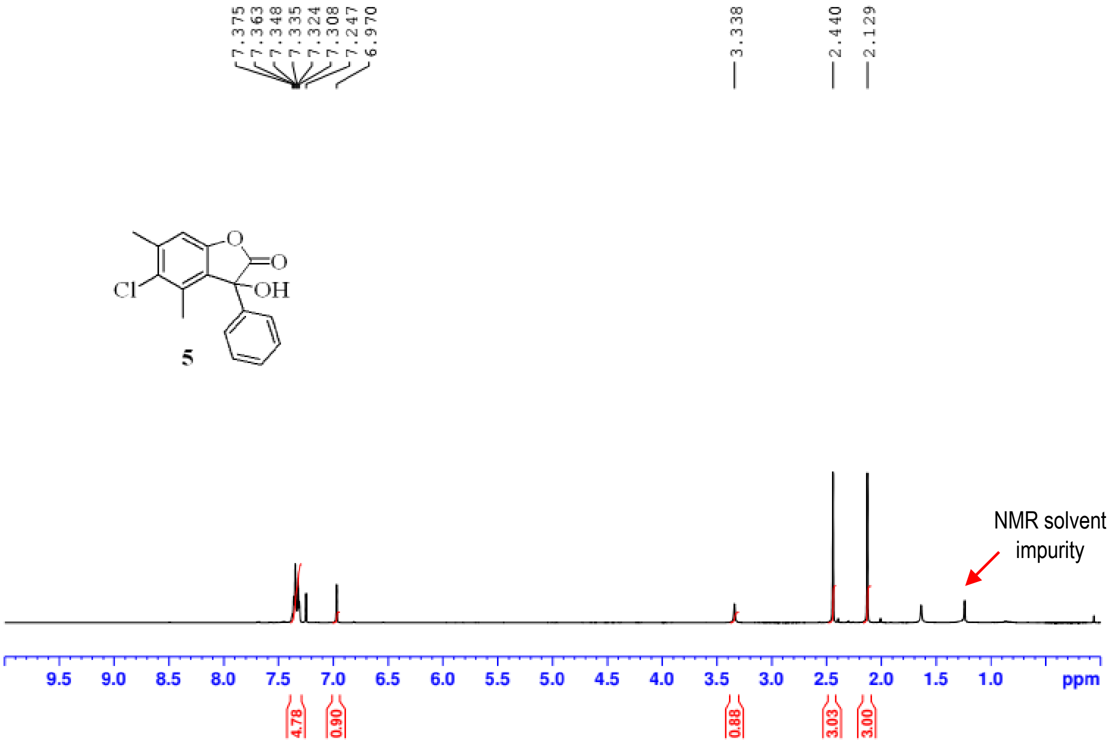

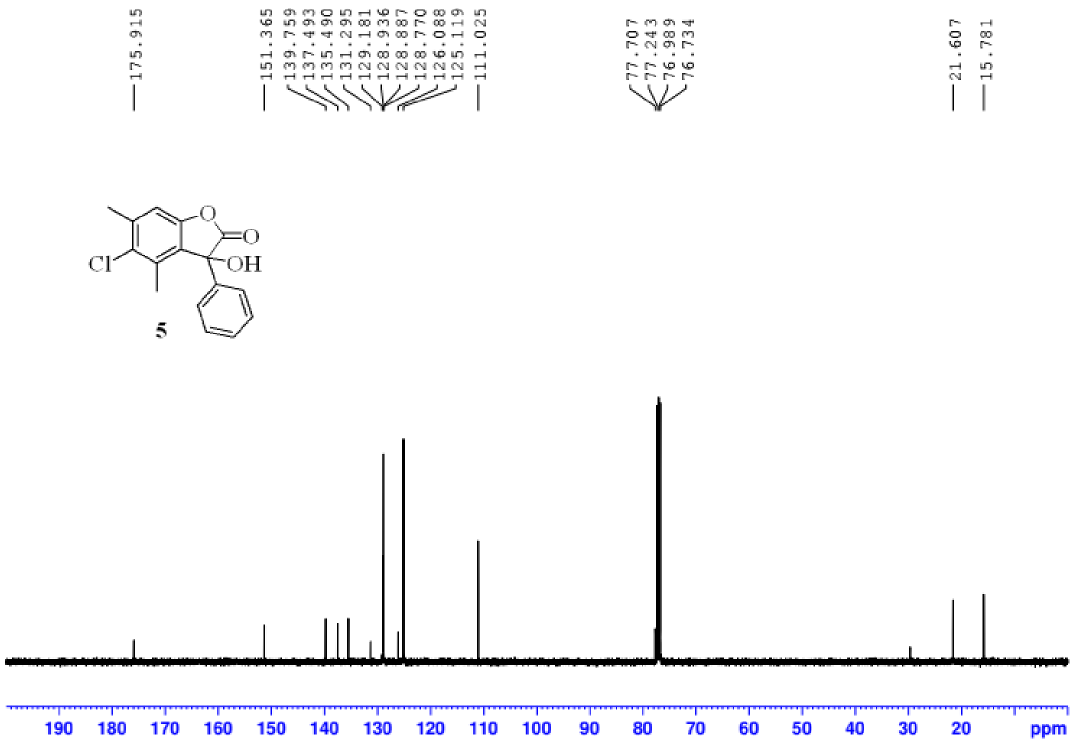

Figure S29. ^{1}H and ^{13}C NMR spectrum of **5**.

Single crystal XRD data for compound **4ja (CCDC no. 2348642)**

checkCIF/PLATON report

Structure factors have been supplied for datablock(s) bod-bd-cs-23-10

THIS REPORT IS FOR GUIDANCE ONLY. IF USED AS PART OF A REVIEW PROCEDURE FOR PUBLICATION, IT SHOULD NOT REPLACE THE EXPERTISE OF AN EXPERIENCED CRYSTALLOGRAPHIC REFEREE.

No syntax errors found.   CIF dictionary   Interpreting this report

Datablock: bod-bd-cs-23-10

```
Bond precision:   C-C = 0.0033 A          Wavelength=1.54184

Cell:            a=15.9403(3)    b=5.8978(1)        c=12.1235(2)
                 alpha=90        beta=106.196(2)    gamma=90
Temperature:     298 K
```

	Calculated	Reported
Volume	1094.53(4)	1094.53(3)
Space group	P 21/c	P 1 21/c 1
Hall group	-P 2ybc	-P 2ybc
Moiety formula	C13 H9 Br O2	C13 H9 Br O2
Sum formula	C13 H9 Br O2	C13 H9 Br O2
Mr	277.10	277.11
Dx,g cm-3	1.682	1.682
Z	4	4
Mu (mm-1)	4.964	4.964
F000	552.0	552.0
F000'	550.59	
h,k,lmax	20,7,15	19,7,15
Nref	2317	2238
Tmin,Tmax	0.639,0.609	0.668,1.000
Tmin'	0.580	

```
Correction method= # Reported T Limits: Tmin=0.668 Tmax=1.000
AbsCorr = MULTI-SCAN

Data completeness= 0.966          Theta(max)= 77.081

R(reflections)= 0.0308( 2018)                  wR2(reflections)=
                                               0.1053( 2238)
S = 1.136                 Npar= 146
```

The following ALERTS were generated. Each ALERT has the format
test-name_ALERT_alert-type_alert-level.
Click on the hyperlinks for more details of the test.

Alert level C

```
PLAT911_ALERT_3_C Missing FCF Refl Between Thmin & STh/L=    0.600         2 Report
                0  7  2, -17  0 10,
```

Alert level G

```
PLAT007_ALERT_5_G Number of Unrefined Donor-H Atoms .............          1 Report
              H1
PLAT912_ALERT_4_G Missing # of FCF Reflections Above STh/L=  0.600        75 Note
PLAT969_ALERT_5_G The 'Henn et al.' R-Factor-gap value ...........      3.98 Note
              Predicted wR2: Based on SigI**2  2.65 or SHELX Weight  9.59
PLAT978_ALERT_2_G Number C-C Bonds with Positive Residual Density.         3 Info
```

0 **ALERT level A** = Most likely a serious problem - resolve or explain
0 **ALERT level B** = A potentially serious problem, consider carefully
1 **ALERT level C** = Check. Ensure it is not caused by an omission or oversight
4 **ALERT level G** = General information/check it is not something unexpected

0 ALERT type 1 CIF construction/syntax error, inconsistent or missing data
1 ALERT type 2 Indicator that the structure model may be wrong or deficient
1 ALERT type 3 Indicator that the structure quality may be low
1 ALERT type 4 Improvement, methodology, query or suggestion
2 ALERT type 5 Informative message, check

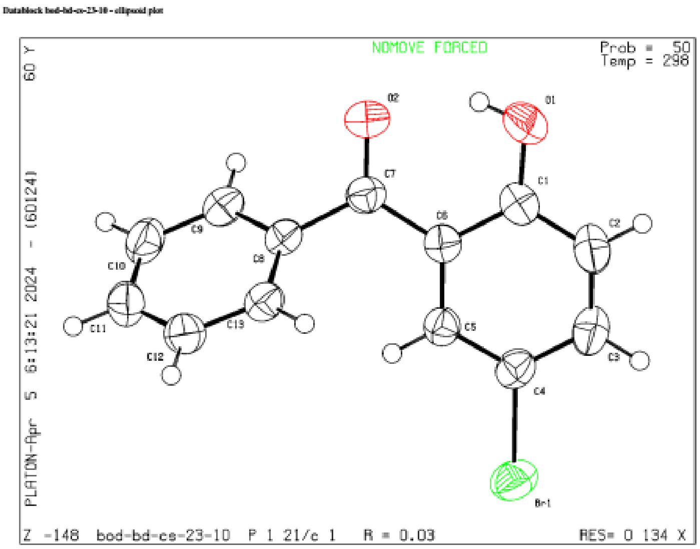

Table S2. Crystal data and structure refinement for **4ja (CCDC no.** 2348642**)**

CCDC number	
Empirical formula	$C_{13}H_9BrO_2$
Formula weight	277.11
Temperature [K]	298
Crystal system	monoclinic
Space group (number)	$P2_1/c$ (14)
a [Å]	15.9403(3)
b [Å]	5.89780(10)
c [Å]	12.1235(2)
α [Å]	90
β [Å]	106.196(2)
γ [Å]	90
Volume [Å^3]	1094.53(3)
Z	4
ρ_{calc} [g/cm^3]	1.682
μ [mm^{-1}]	4.964
F(000)	552
Crystal size [mm^3]	0.1×0.1×0.1
Crystal colour	colourless
Crystal shape	block
Radiation	Cu K_α (λ=1.54184 Å)
2θ range [°]	5.77 to 154.16 (0.79 Å)
Index ranges	$-19 \le h \le 19$<br>$-7 \le k \le 7$<br>$-14 \le l \le 15$
Reflections collected	12336
Independent reflections	2238<br>R_{int} = 0.0590<br>R_{sigma} = 0.0297
Completeness to θ = 67.684°	99.9 %
Data / Restraints / Parameters	2238/0/146
Goodness-of-fit on F^2	1.136
Final R indexes [$I \ge 2\sigma(I)$]	R_1 = 0.0308<br>wR_2 = 0.0838
Final R indexes [all data]	R_1 = 0.0554<br>wR_2 = 0.1053
Largest peak/hole [eÅ^3]	0.63/-1.35

Table S3. Bond lengths and angles for **4ja (CCDC no. 2348642)**

Atom–AtomAtom–Atom–Atom	Length [Å]Angle [°]
Br1–C4	1.897(3)
O1–H1	0.8200
O1–C1	1.344(3)
C1–C2	1.385(4)
C1–C6	1.413(3)
O2–C7	1.231(3)
C2–C3	1.374(4)
C3–C4	1.388(4)
C4–C5	1.377(3)
C5–C6	1.398(3)
C6–C7	1.472(3)
C7–C8	1.496(3)
C8–C9	1.389(3)
C8–C13	1.390(4)
C9–C10	1.387(4)
C10–C11	1.377(4)
C11–C12	1.377(4)
C12–C13	1.383(4)
C1–O1–H1	109.5
O1–C1–C2	117.8(2)
O1–C1–C6	122.5(2)
C2–C1–C6	119.7(2)
C3–C2–C1	120.8(2)
C2–C3–C4	119.4(2)
C3–C4–Br1	119.60(19)
C5–C4–Br1	119.08(18)
C5–C4–C3	121.3(2)
C4–C5–C6	119.6(2)
C1–C6–C7	119.2(2)
C5–C6–C1	119.1(2)
C5–C6–C7	121.6(2)
O2–C7–C6	121.4(2)
O2–C7–C8	118.1(2)
C6–C7–C8	120.4(2)
C9–C8–C7	118.1(2)
C9–C8–C13	119.8(2)
C13–C8–C7	122.0(2)
C10–C9–C8	119.9(2)
C11–C10–C9	119.9(3)
C10–C11–C12	120.5(3)
C11–C12–C13	120.2(3)
C12–C13–C8	119.7(2)

Table S4. Torsion angles for **4ja (CCDC no.** 2348642**)**.

Atom–Atom–Atom–Atom	Torsion Angle [°]
Br1–C4–C5–C6	-177.32(17)
O1–C1–C2–C3	-176.8(3)
O1–C1–C6–C5	176.2(2)
O1–C1–C6–C7	-0.2(4)
C1–C2–C3–C4	0.0(4)
C1–C6–C7–O2	9.4(4)
C1–C6–C7–C8	-170.9(2)
O2–C7–C8–C9	48.7(3)
O2–C7–C8–C13	-127.0(3)
C2–C1–C6–C5	-3.6(4)
C2–C1–C6–C7	-180.0(2)
C2–C3–C4–Br1	176.7(2)
C2–C3–C4–C5	-2.5(4)
C3–C4–C5–C6	2.0(4)
C4–C5–C6–C1	1.1(3)
C4–C5–C6–C7	177.4(2)
C5–C6–C7–O2	-166.9(2)
C5–C6–C7–C8	12.8(3)
C6–C1–C2–C3	3.1(4)
C6–C7–C8–C9	-131.0(2)
C6–C7–C8–C13	53.2(3)
C7–C8–C9–C10	-176.6(2)
C7–C8–C13–C12	174.0(2)
C8–C9–C10–C11	2.1(4)
C9–C8–C13–C12	-1.8(4)
C9–C10–C11–C12	-1.1(4)
C10–C11–C12–C13	-1.4(4)
C11–C12–C13–C8	2.8(4)
C13–C8–C9–C10	-0.7(4)

Single crystal XRD data for compound **4fb (CCDC no. 2348641)**

checkCIF/PLATON report

Structure factors have been supplied for datablock(s) shelx

THIS REPORT IS FOR GUIDANCE ONLY. IF USED AS PART OF A REVIEW PROCEDURE FOR PUBLICATION, IT SHOULD NOT REPLACE THE EXPERTISE OF AN EXPERIENCED CRYSTALLOGRAPHIC REFEREE.

No syntax errors found. CIF dictionary Interpreting this report

Datablock: shelx

```
Bond precision:   C-C = 0.0081 A          Wavelength=1.54184

Cell:            a=13.9577(3)    b=13.9083(3)       c=33.1018(8)
                 alpha=90        beta=100.790(2)    gamma=90
Temperature:     298 K

                 Calculated                 Reported
Volume           6312.4(2)                  6312.4(2)
Space group      C c                        C c
Hall group       C -2yc                     C -2yc
Moiety formula   C18 H20 O3                 ?
Sum formula      C18 H20 O3                 C18 H20 O3
Mr               284.34                     284.34
Dx,g cm-3        1.197                      1.197
Z                16                         16
Mu (mm-1)        0.645                      0.645
F000             2432.0                     2432.0
F000'            2439.30
h,k,lmax         17,17,42                   17,17,41
Nref             13828[ 6918]               11098
Tmin,Tmax        0.940,0.994                0.633,1.000
Tmin'            0.938

Correction method= # Reported T Limits: Tmin=0.633 Tmax=1.000
AbsCorr = MULTI-SCAN

Data completeness= 1.60/0.80       Theta(max)= 80.243

                                                 wR2(reflections)=
R(reflections)= 0.0748( 8167)                    0.2966( 11098)

S = 1.188               Npar= 874
```

```
The following ALERTS were generated. Each ALERT has the format
       test-name_ALERT_alert-type_alert-level.
Click on the hyperlinks for more details of the test.
```

Alert level A

```
SHFSU01_ALERT_2_A  The absolute value of parameter shift to su ratio > 0.20
            Absolute value of the parameter shift to su ratio given   0.541
            Additional refinement cycles may be required.
```

Author Response: May be due to rotational disorder in t-but groups and the oscillating H-atom on hydroxyl group in para paostion to it The refinement did not converge even after multiple L.S. cycles.

```
PLAT080_ALERT_2_A Maximum Shift/Error ............................       0.54 Why ?
```

Author Response: May be due to rotational disorder in t-but groups and the oscillating H-atom on hydroxyl group in para paostion to it The refinement did not converge even after multiple L.S. cycles.

Alert level C

```
STRVA01_ALERT_4_C          Flack parameter is too small
           From the CIF: _refine_ls_abs_structure_Flack   -0.300
           From the CIF: _refine_ls_abs_structure_Flack_su    0.400
PLAT084_ALERT_3_C High wR2 Value (i.e. > 0.25) ...................       0.30 Report
PLAT213_ALERT_2_C Atom C15B           has ADP max/min Ratio .....        3.4 prolat
PLAT213_ALERT_2_C Atom C51A           has ADP max/min Ratio .....        3.6 prolat
PLAT213_ALERT_2_C Atom C53B           has ADP max/min Ratio .....        3.4 prolat
PLAT220_ALERT_2_C NonSolvent   Resd 3  C    Ueq(max)/Ueq(min) Range       3.3 Ratio
PLAT242_ALERT_2_C Low    'MainMol' Ueq as Compared to Neighbors of       C14 Check
PLAT242_ALERT_2_C Low    'MainMol' Ueq as Compared to Neighbors of       C32 Check
PLAT242_ALERT_2_C Low    'MainMol' Ueq as Compared to Neighbors of       C50 Check
PLAT242_ALERT_2_C Low    'MainMol' Ueq as Compared to Neighbors of       C68 Check
PLAT340_ALERT_3_C Low Bond Precision on  C-C Bonds ...............    0.00815 Ang.
PLAT911_ALERT_3_C Missing FCF Refl Between Thmin & STh/L=    0.600          2 Report
              -16  0  6,    9  3 19,
PLAT927_ALERT_1_C Reported and Calculated  wR2 Differ by .........    -0.0025 Check
```

Alert level G

```
PLAT002_ALERT_2_G Number of Distance or Angle Restraints on AtSite         28 Note
PLAT003_ALERT_2_G Number of Uiso or Uij Restrained non-H Atoms ...         68 Report
PLAT007_ALERT_5_G Number of Unrefined Donor-H Atoms ..............          4 Report
              H3A   H6A   H9    H12
PLAT032_ALERT_4_G Std. Uncertainty on Flack Parameter Value High .      0.400 Report
PLAT172_ALERT_4_G The CIF-Embedded .res File Contains DFIX Records         16 Report
PLAT177_ALERT_4_G The CIF-Embedded .res File Contains DELU Records         12 Report
PLAT178_ALERT_4_G The CIF-Embedded .res File Contains SIMU Records         12 Report
PLAT301_ALERT_3_G Main Residue  Disorder ..............(Resd    1)        14% Note
PLAT301_ALERT_3_G Main Residue  Disorder ..............(Resd    2)        14% Note
```

```
PLAT301_ALERT_3_G Main Residue  Disorder ..............(Resd    3)        14% Note
PLAT301_ALERT_3_G Main Residue  Disorder ..............(Resd    4)        14% Note
PLAT412_ALERT_2_G Short Intra XH3 .. XHn     H11      ..H16D     .       2.03 Ang.
                                                     x,y,z  =     1_555 Check
PLAT412_ALERT_2_G Short Intra XH3 .. XHn     H29      ..H33A     .       1.97 Ang.
                                                     x,y,z  =     1_555 Check
PLAT412_ALERT_2_G Short Intra XH3 .. XHn     H31      ..H34A     .       2.05 Ang.
                                                     x,y,z  =     1_555 Check
PLAT412_ALERT_2_G Short Intra XH3 .. XHn     H47      ..H51C     .       1.97 Ang.
                                                     x,y,z  =     1_555 Check
PLAT412_ALERT_2_G Short Intra XH3 .. XHn     H49      ..H53A     .       2.14 Ang.
                                                     x,y,z  =     1_555 Check
PLAT412_ALERT_2_G Short Intra XH3 .. XHn     H49      ..H53E     .       2.01 Ang.
                                                     x,y,z  =     1_555 Check
PLAT412_ALERT_2_G Short Intra XH3 .. XHn     H65      ..H69B     .       2.04 Ang.
                                                     x,y,z  =     1_555 Check
PLAT412_ALERT_2_G Short Intra XH3 .. XHn     H67      ..H70F     .       2.12 Ang.
                                                     x,y,z  =     1_555 Check
PLAT721_ALERT_1_G Bond    Calc     0.97000, Rep     0.96000 Dev...       0.01 Ang.
              C17B     -H17D          1_555   1_555 ........     #   47 Check
PLAT721_ALERT_1_G Bond    Calc     0.97000, Rep     0.96000 Dev...       0.01 Ang.
              C51B     -H51D          1_555   1_555 ........     #  147 Check
PLAT722_ALERT_1_G Angle   Calc      111.00, Rep      109.50 Dev...       1.50 Degree
              H15D    -C15B    -H15E       1_555  1_555  1_555     #   72 Check
PLAT722_ALERT_1_G Angle   Calc      109.00, Rep      110.10 Dev...       1.10 Degree
              C14     -C15B    -H15F       1_555  1_555  1_555     #   73 Check
PLAT722_ALERT_1_G Angle   Calc      109.00, Rep      110.10 Dev...       1.10 Degree
              C14     -C16B    -H16D       1_555  1_555  1_555     #   76 Check
PLAT722_ALERT_1_G Angle   Calc      110.00, Rep      108.90 Dev...       1.10 Degree
              C14     -C16B    -H16E       1_555  1_555  1_555     #   77 Check
PLAT722_ALERT_1_G Angle   Calc      108.00, Rep      109.10 Dev...       1.10 Degree
              C14     -C17B    -H17D       1_555  1_555  1_555     #   82 Check
PLAT722_ALERT_1_G Angle   Calc      111.00, Rep      109.50 Dev...       1.50 Degree
              H17E    -C17B    -H17F       1_555  1_555  1_555     #   87 Check
PLAT860_ALERT_3_G Number of Least-Squares Restraints .............        656 Note
PLAT883_ALERT_1_G No Info/Value for _atom_sites_solution_primary .     Please Do !
PLAT899_ALERT_4_G SHELXL2018 is Deprecated and Succeeded by SHELXL     2019/3 Note
PLAT912_ALERT_4_G Missing # of FCF Reflections Above STh/L=  0.600        294 Note
PLAT915_ALERT_3_G No Flack x Check Done: Low Friedel Pair Coverage         65 %
PLAT916_ALERT_2_G Hooft y and Flack x Parameter Values Differ by .       0.37 Check
PLAT933_ALERT_2_G Number of HKL-OMIT Records in Embedded .res File         70 Note
                6 -2 -2,   8  2  0,   9 -1-27,  -7  7 26,  11 -7 -2,   9 -9-14,
               10  2 17,   7 -3 -2,  10  4 17,  11  7  8,  10  4 15,   9 -3-27,
                9  1 20,  10  6 12,   1  1 32,   9 -9-15,   8-10-13,   8 -8-21,
                1  1 31,   9 -9-16,   8  2-27,  10  6 11,   9  1-27,   9  3-26,
                9  9-15,  10  2 18,   9  3-27,   9  3 20,   8  4-27,  10  2-27,
                9  9-14,   8  4-26,  11  7 -3,  11  7 -4,  11  5 10,   8 10-15,
                3 11 16,   8 10-16,  11  7  1,   7  1 25,  11  7 -1,  -9  3-19,
               11  7  4,   9  7-22,  11  7  7,   9  3 19,  10  2-26,   9  3-25,
                7  1 24,   9  7-20,
PLAT969_ALERT_5_G The 'Henn et al.' R-Factor-gap value ...........       4.50 Note
              Predicted wR2: Based on SigI**2  6.59 or SHELX Weight 26.04
PLAT978_ALERT_2_G Number C-C Bonds with Positive Residual Density.          0 Info
```

---

2 **ALERT level A** = Most likely a serious problem - resolve or explain

0 **ALERT level B** = A potentially serious problem, consider carefully

```
13 ALERT level C = Check. Ensure it is not caused by an omission or oversight
36 ALERT level G = General information/check it is not something unexpected

10 ALERT type 1 CIF construction/syntax error, inconsistent or missing data
23 ALERT type 2 Indicator that the structure model may be wrong or deficient
 9 ALERT type 3 Indicator that the structure quality may be low
 7 ALERT type 4 Improvement, methodology, query or suggestion
 2 ALERT type 5 Informative message, check
```

It is advisable to attempt to resolve as many as possible of the alerts in all categories. Often the minor alerts point to easily fixed oversights, errors and omissions in your CIF or refinement strategy, so attention to these fine details can be worthwhile. In order to resolve some of the more serious problems it may be necessary to carry out additional measurements or structure refinements. However, the purpose of your study may justify the reported deviations and the more serious of these should normally be commented upon in the discussion or experimental section of a paper or in the "special_details" fields of the CIF. checkCIF was carefully designed to identify outliers and unusual parameters, but every test has its limitations and alerts that are not important in a particular case may appear. Conversely, the absence of alerts does not guarantee there are no aspects of the results needing attention. It is up to the individual to critically assess their own results and, if necessary, seek expert advice.

Publication of your CIF in IUCr journals

A basic structural check has been run on your CIF. These basic checks will be run on all CIFs submitted for publication in IUCr journals (*Acta Crystallographica*, *Journal of Applied Crystallography*, *Journal of Synchrotron Radiation*); however, if you intend to submit to *Acta Crystallographica Section C* or *E* or *IUCrData*, you should make sure that full publication checks are run on the final version of your CIF prior to submission.

Publication of your CIF in other journals

Please refer to the *Notes for Authors* of the relevant journal for any special instructions relating to CIF submission.

PLATON version of 06/01/2024; check.def file version of 05/01/2024

Datablock shelx - ellipsoid plot

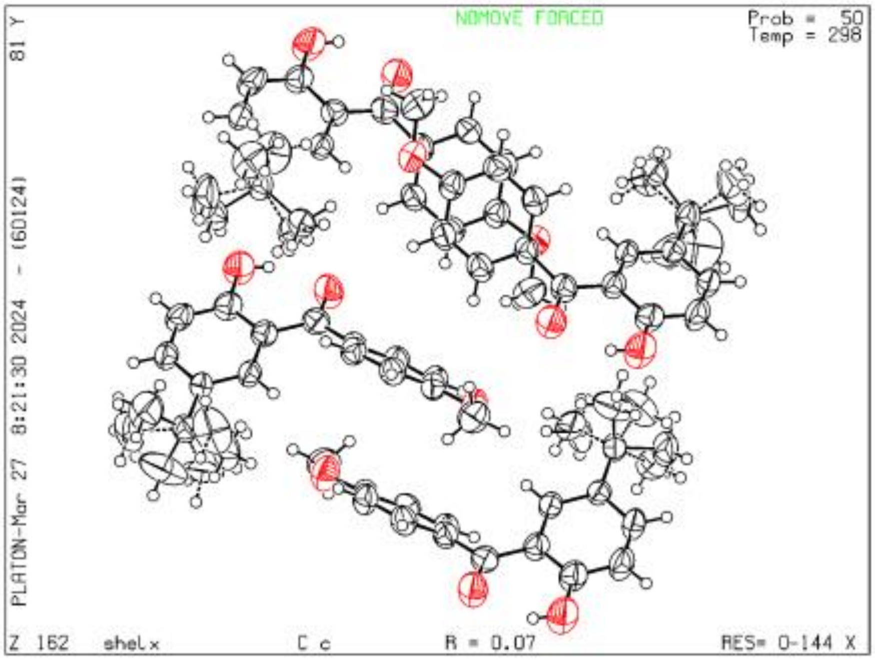

Table S5. Crystal data and structure refinement for shelx (**<u>4fb : CCDC no. 2348641)</u>**

CCDC number	
Empirical formula	$C_{18}H_{20}O_3$
Formula weight	284.34
Temperature [K]	298(2)
Crystal system	monoclinic
Space group (number)	*Cc* (9)
a [Å]	13.9577(3)
b [Å]	13.9083(3)
c [Å]	33.1018(8)
α [Å]	90
β [Å]	100.790(2)
γ [Å]	90
Volume [Å^3]	6312.4(2)
Z	16
ρ_{calc} [g/cm^3]	1.197
μ [mm^{-1}]	0.645
F(000)	2432
Crystal size [mm^3]	0.100×0.080×0.010
Crystal colour	colourless
Crystal shape	block
Radiation	Cu K_α (λ=1.54184 Å)
2Θ range [°]	5.44 to 160.49 (0.78 Å)
Index ranges	-17 ≤ h ≤ 15
	-17 ≤ k ≤ 17
	-41 ≤ l ≤ 41
Reflections collected	44813
Independent reflections	11098
	R_{int} = 0.0907
	R_{sigma} = 0.0618
Completeness to θ = 67.684°	100.0 %
Data / Restraints / Parameters	11098/656/874
Goodness-of-fit on F^2	1.188
Final *R* indexes	R_1 = 0.0748
[I≥2σ(I)]	wR_2 = 0.2099
Final *R* indexes	R_1 = 0.1240
[all data]	wR_2 = 0.2966
Largest peak/hole [eÅ^3]	0.57/-0.37
Flack X parameter	-0.3(4)

Table S6. Bond lengths and angles for shelx (**4fb : CCDC no. 2348641)**

Atom–Atom	Length [Å]	–		–		–	
C1–C2	1.384(9)	C19–C20	1.380(9)	C37–C38	1.384(9)	C55–C56	1.388(9)
C1–C6	1.381(8)	C19–C24	1.392(8)	C37–C42	1.384(8)	C55–C60	1.391(8)
C1–O1	1.372(6)	C19–O4	1.360(6)	C37–O7	1.372(7)	C55–O10	1.351(7)
C2–C3	1.368(8)	C20–C21	1.380(8)	C38–C39	1.369(9)	C56–C57	1.370(8)
C3–C4	1.418(7)	C21–C22	1.409(8)	C39–C40	1.400(8)	C57–C58	1.407(8)
C4–C5	1.379(8)	C22–C23	1.369(8)	C40–C41	1.381(8)	C58–C59	1.380(8)
C4–C7	1.489(7)	C22–C25	1.496(7)	C40–C43	1.498(7)	C58–C61	1.484(7)
C5–C6	1.397(7)	C23–C24	1.391(8)	C41–C42	1.385(8)	C59–C60	1.387(8)
C7–C8	1.475(7)	C25–C26	1.468(8)	C43–C44	1.470(7)	C61–C62	1.486(8)
C7–O2	1.239(7)	C25–O5	1.235(7)	C43–O8	1.232(7)	C61–O11	1.238(7)
C8–C13	1.399(7)	C26–C27	1.418(7)	C44–C45	1.418(7)	C62–C63	1.414(7)
C8–C9	1.421(7)	C26–C31	1.399(7)	C44–C49	1.399(7)	C62–C67	1.398(7)
C9–C10	1.376(9)	C27–C28	1.391(9)	C45–C46	1.408(9)	C63–C64	1.377(9)
C9–O3	1.343(7)	C27–O6	1.343(8)	C45–O9	1.333(7)	C63–O12	1.351(7)
C10–C11	1.369(9)	C28–C29	1.345(9)	C46–C47	1.353(9)	C64–C65	1.388(9)
C11–C12	1.408(7)	C29–C30	1.406(7)	C47–C48	1.408(8)	C65–C66	1.402(7)
C12–C13	1.379(7)	C30–C31	1.390(7)	C48–C49	1.380(7)	C66–C67	1.385(7)
C12–C14	1.528(8)	C30–C32	1.526(8)	C48–C50	1.521(8)	C66–C68	1.518(7)
C14–C15A	1.529(10)	C32–C33A	1.487(14)	C50–C51A	1.495(15)	C68–C69A	1.524(19)
C14–C15B	1.51(2)	C32–C33B	1.582(17)	C50–C51B	1.558(19)	C68–C69B	1.561(11)
C14–C16A	1.557(11)	C32–C34A	1.515(15)	C50–C52A	1.572(17)	C68–C70A	1.55(2)
C14–C16B	1.53(2)	C32–C34B	1.526(13)	C50–C52B	1.54(2)	C68–C70B	1.542(12)
C14–C17A	1.520(11)	C32–C35A	1.614(16)	C50–C53A	1.527(15)	C68–C71A	1.60(2)
C14–C17B	1.58(2)	C32–C35B	1.524(17)	C50–C53B	1.545(17)	C68–C71B	1.522(12)
C18–O1	1.412(9)	C36–O4	1.429(10)	C54–O7	1.418(9)	C72–O10	1.414(9)
O3–H3A	0.8201	O6–H6A	0.8200	O9–H9	0.8200	O12–H12	0.8201
Atom–Atom–Atom	Angle [°]	—		—		—	
C6–C1–C2	120.6(5)	O4–C19–C20	115.5(5)	O7–C37–C38	114.8(5)	O10–C55–C56	115.3(5)
O1–C1–C2	114.4(5)	C20–C19–C24	119.7(5)	C38–C37–C42	120.2(5)	C56–C55–C60	119.3(5)
O1–C1–C6	125.0(5)	O4–C19–C24	124.7(5)	O7–C37–C42	125.1(5)	O10–C55–C60	125.3(5)
C3–C2–C1	120.3(5)	C21–C20–C19	120.9(5)	C39–C38–C37	120.2(5)	C57–C56–C55	121.0(5)
C2–C3–C4	120.4(5)	C20–C21–C22	119.5(5)	C38–C39–C40	120.3(6)	C56–C57–C58	120.1(5)
C5–C4–C3	118.3(5)	C23–C22–C21	119.2(5)	C41–C40–C39	119.0(5)	C59–C58–C57	118.8(5)
C3–C4–C7	117.0(5)	C21–C22–C25	116.5(5)	C39–C40–C43	117.0(5)	C57–C58–C61	117.1(5)
C5–C4–C7	124.6(5)	C23–C22–C25	124.2(5)	C41–C40–C43	123.8(5)	C59–C58–C61	124.0(5)
C4–C5–C6	121.4(5)	C22–C23–C24	121.4(5)	C40–C41–C42	120.7(5)	C58–C59–C60	121.1(5)
C1–C6–C5	119.0(5)	C19–C24–C23	119.2(5)	C41–C42–C37	119.5(5)	C59–C60–C55	119.7(5)
C8–C7–C4	121.0(4)	C26–C25–C22	121.3(4)	C44–C43–C40	121.1(4)	O11–C61–C58	118.1(5)
O2–C7–C4	117.7(5)	O5–C25–C22	117.5(5)	O8–C43–C40	118.0(5)	C58–C61–C62	121.2(4)
O2–C7–C8	121.2(5)	O5–C25–C26	121.1(5)	O8–C43–C44	120.9(5)	O11–C61–C62	120.6(5)
C13–C8–C7	122.3(5)	C27–C26–C25	119.4(5)	C45–C44–C43	119.0(5)	C63–C62–C61	119.2(5)
C9–C8–C7	119.2(5)	C31–C26–C25	122.2(4)	C49–C44–C43	122.6(4)	C67–C62–C61	122.1(5)
C13–C8–C9	118.4(5)	C31–C26–C27	118.4(5)	C49–C44–C45	118.2(5)	C67–C62–C63	118.7(5)
O3–C9–C10	118.7(5)	C28–C27–C26	118.6(5)	C46–C45–C44	118.4(5)	C64–C63–C62	119.3(5)
C10–C9–C8	119.1(5)	O6–C27–C26	122.3(6)	O9–C45–C44	123.1(5)	O12–C63–C62	122.4(5)
O3–C9–C8	122.2(5)	O6–C27–C28	119.0(5)	O9–C45–C46	118.5(5)	O12–C63–C64	118.3(5)
C11–C10–C9	120.9(5)	C29–C28–C27	121.1(5)	C47–C46–C45	120.8(5)	C63–C64–C65	120.7(5)
C10–C11–C12	121.9(5)	C28–C29–C30	123.0(6)	C46–C47–C48	122.7(5)	C64–C65–C66	121.5(5)
C13–C12–C11	116.9(5)	C31–C30–C29	116.0(5)	C49–C48–C47	116.2(5)	C67–C66–C65	117.2(5)
C11–C12–C14	120.1(5)	C29–C30–C32	121.3(5)	C47–C48–C50	120.4(5)	C65–C66–C68	119.9(5)
C13–C12–C14	123.0(5)	C31–C30–C32	122.7(5)	C49–C48–C50	123.3(5)	C67–C66–C68	122.9(5)
C12–C13–C8	122.6(5)	C30–C31–C26	122.8(5)	C48–C49–C44	123.6(5)	C66–C67–C62	122.6(5)
C15B–C14–C12	111(2)	C33A–C32–C30	113.9(9)	C51A–C50–C48	109.6(10)	C66–C68–C69A	112.9(17)
C17A–C14–C12	111.1(7)	C34A–C32–C30	114.1(10)	C48–C50–C51B	111.7(14)	C66–C68–C69B	110.2(6)
C12–C14–C15A	112.3(5)	C34B–C32–C30	110.0(9)	C52B–C50–C51B	109.4(18)	C70B–C68–C69B	107.5(8)
C17A–C14–C15A	107.8(8)	C35B–C32–C30	114.2(12)	C53B–C50–C51B	105.9(14)	C71B–C68–C69B	108.5(8)
C12–C14–C16A	108.6(6)	C30–C32–C33B	106.7(8)	C48–C50–C52A	105.2(11)	C66–C68–C70A	118.8(17)
C15A–C14–C16A	106.9(7)	C34B–C32–C33B	107.4(12)	C51A–C50–C52A	110.8(14)	C69A–C68–C70A	110.8(17)
C17A–C14–C16A	110.1(9)	C35B–C32–C33B	108.1(11)	C53A–C50–C52A	106.9(11)	C66–C68–C70B	110.7(7)
C12–C14–C16B	112.3(19)	C33A–C32–C34A	112.5(12)	C48–C50–C52B	111(2)	C71B–C68–C70B	108.4(9)
C15B–C14–C16B	114.0(19)	C35B–C32–C34B	110.1(13)	C48–C50–C53A	114.3(9)	C66–C68–C71A	98.9(19)
C12–C14–C17B	98.8(19)	C30–C32–C35A	101.8(10)	C51A–C50–C53A	109.9(11)	C69A–C68–C71A	107.7(15)
C15B–C14–C17B	111(2)	C33A–C32–C35A	106.3(11)	C48–C50–C53B	107.8(12)	C70A–C68–C71A	106.2(18)
C16B–C14–C17B	109(2)	C34A–C32–C35A	107.1(11)	C52B–C50–C53B	110.6(17)	C66–C68–C71B	111.4(9)
C1–O1–C18	118.5(5)	C19–O4–C36	119.7(6)	C37–O7–C54	119.3(5)	C55–O10–C72	118.8(5)

Table S7. Hydrogen bonds for shelx (**4fb : CCDC no. 2348641)**

D–H··A [Å]	d(D–H) [Å]	d(H··A) [Å]	d(D··A) [Å]	<(DHA) [°]
O3–H3A··O2	0.82	1.85	2.574(6)	146.1
O6–H6A··O5	0.82	1.86	2.580(7)	145.7
O9–H9··O8	0.82	1.86	2.575(7)	145.5
O12–H12··O11	0.82	1.86	2.565(7)	143.1
O3–H3A··O2	0.82	1.85	2.574(6)	146.1
O6–H6A··O5	0.82	1.86	2.580(7)	145.7
O9–H9··O8	0.82	1.86	2.575(7)	145.5
O12–H12··O11	0.82	1.86	2.565(7)	143.1

Table S8. Torsion angles for shelx (**4fb : CCDC no. 2348641)**

			Torsion Angle [°]	——		——	
C1–C2–C3–C4	-0.6(9)	C37–C38–C39–C40	-4.0(10)	C19–C20–C21–C22	2.9(9)	C55–C56–C57–C58	0.7(9)
C2–C3–C4–C5	0.0(8)	C38–C39–C40–C41	2.7(9)	C20–C21–C22–C23	-1.1(9)	C56–C57–C58–C59	-0.2(9)
C2–C3–C4–C7	177.3(5)	C38–C39–C40–C43	178.6(6)	C20–C21–C22–C25	-178.3(5)	C56–C57–C58–C61	-177.0(6)
C2–C1–C6–C5	1.0(8)	C38–C37–C42–C41	0.1(9)	C20–C19–C24–C23	0.6(8)	C56–C55–C60–C59	-1.8(9)
C2–C1–O1–C18	-176.0(6)	C38–C37–O7–C54	-176.9(6)	C20–C19–O4–C36	176.5(6)	C56–C55–O10–C72	175.6(6)
C3–C4–C5–C6	1.1(8)	C39–C40–C41–C42	0.0(8)	C21–C22–C23–C24	-0.9(9)	C57–C58–C59–C60	-1.3(8)
C3–C4–C7–O2	-42.2(8)	C39–C40–C43–O8	-42.4(8)	C21–C22–C25–O5	42.7(8)	C57–C58–C61–O11	43.0(8)
C3–C4–C7–C8	136.3(6)	C39–C40–C43–C44	136.6(6)	C21–C22–C25–C26	-136.7(6)	C57–C58–C61–C62	-134.2(6)
C4–C5–C6–C1	-1.6(9)	C40–C41–C42–C37	-1.4(9)	C22–C23–C24–C19	1.2(9)	C58–C59–C60–C55	2.3(9)
C4–C7–C8–C13	-11.8(9)	C40–C43–C44–C49	-12.7(9)	C22–C25–C26–C31	13.3(9)	C58–C61–C62–C67	8.7(9)
C4–C7–C8–C9	172.5(6)	C40–C43–C44–C45	171.5(6)	C22–C25–C26–C27	-168.9(6)	C58–C61–C62–C63	-173.9(5)
C5–C4–C7–O2	134.8(7)	C41–C40–C43–O8	133.4(6)	C23–C22–C25–O5	-134.3(7)	C59–C58–C61–O11	-133.6(7)
C5–C4–C7–C8	-46.6(8)	C41–C40–C43–C44	-47.7(8)	C23–C22–C25–C26	46.2(9)	C59–C58–C61–C62	49.1(8)
C6–C1–C2–C3	0.1(9)	C42–C37–C38–C39	2.6(9)	C24–C19–C20–C21	-2.6(9)	C60–C55–C56–C57	0.3(9)
C6–C1–O1–C18	4.1(9)	C42–C37–O7–C54	3.2(10)	C24–C19–O4–C36	-2.9(9)	C60–C55–O10–C72	-5.2(10)
C7–C4–C5–C6	-176.0(5)	C43–C40–C41–C42	-175.6(5)	C25–C22–C23–C24	176.1(5)	C61–C58–C59–C60	175.3(5)
C7–C8–C9–O3	-1.9(10)	C43–C44–C45–O9	-1.4(10)	C25–C26–C27–O6	0.2(11)	C61–C62–C63–O12	0.0(10)
C7–C8–C9–C10	-179.1(6)	C43–C44–C45–C46	179.5(6)	C25–C26–C27–C28	179.7(7)	C61–C62–C63–C64	179.4(6)
C7–C8–C13–C12	179.4(6)	C43–C44–C49–C48	-179.4(6)	C25–C26–C31–C30	179.3(6)	C61–C62–C67–C66	179.9(5)
C8–C9–C10–C11	-2.9(10)	C44–C45–C46–C47	-2.0(11)	C26–C27–C28–C29	1.6(13)	C62–C63–C64–C65	1.5(11)
C9–C10–C11–C12	0.4(10)	C45–C46–C47–C48	0.3(11)	C27–C28–C29–C30	0.1(12)	C63–C64–C65–C66	0.8(10)
C9–C8–C13–C12	-4.8(9)	C45–C44–C49–C48	-3.6(9)	C27–C26–C31–C30	1.5(10)	C63–C62–C67–C66	2.5(9)
C10–C11–C12–C13	0.0(9)	C46–C47–C48–C49	-0.2(10)	C28–C29–C30–C31	-1.1(10)	C64–C65–C66–C67	-1.5(9)
C10–C11–C12–C14	-178.6(6)	C46–C47–C48–C50	-177.7(6)	C28–C29–C30–C32	178.2(7)	C64–C65–C66–C68	178.8(6)
C11–C12–C13–C8	2.3(9)	C47–C48–C49–C44	1.9(9)	C29–C30–C31–C26	0.2(9)	C65–C66–C67–C62	-0.2(9)
C11–C12–C14–C15B	-134(3)	C47–C48–C50–C51A	-33.3(17)	C29–C30–C32–C33A	20.7(19)	C65–C66–C68–C71B	-68.9(11)
C11–C12–C14–C17A	69.1(11)	C47–C48–C50–C53A	-157.2(12)	C29–C30–C32–C34A	151.7(13)	C65–C66–C68–C69A	24(3)
C11–C12–C14–C15A	-170.1(8)	C47–C48–C50–C52B	68(2)	C29–C30–C32–C35B	-66.3(17)	C65–C66–C68–C70B	170.4(9)
C11–C12–C14–C16B	-5(3)	C47–C48–C50–C53B	-170.2(18)	C29–C30–C32–C34B	169.3(15)	C65–C66–C68–C70A	156(2)
C11–C12–C14–C16A	-52.1(9)	C47–C48–C50–C51B	-54(2)	C29–C30–C32–C33B	53.1(15)	C65–C66–C68–C69B	51.6(11)
C11–C12–C14–C17B	109(3)	C47–C48–C50–C52A	85.9(16)	C29–C30–C32–C35A	-93.3(13)	C65–C66–C68–C71A	-89(2)
C13–C8–C9–O3	-177.8(7)	C49–C44–C45–O9	-177.4(7)	C31–C26–C27–O6	178.1(7)	C67–C62–C63–O12	177.4(7)
C13–C8–C9–C10	5.0(9)	C49–C44–C45–C46	3.5(10)	C31–C26–C27–C28	-2.4(11)	C67–C62–C63–C64	-3.1(9)
C13–C12–C14–C15B	47(3)	C49–C48–C50–C51A	149.3(17)	C31–C30–C32–C33A	-160.1(18)	C67–C66–C68–C71B	111.4(11)
C13–C12–C14–C17A	-109.5(11)	C49–C48–C50–C53A	25.4(14)	C31–C30–C32–C34A	-29.1(14)	C67–C66–C68–C69A	-156(3)
C13–C12–C14–C15A	11.4(10)	C49–C48–C50–C52B	-109(2)	C31–C30–C32–C35B	112.9(17)	C67–C66–C68–C70B	-9.2(11)
C13–C12–C14–C16B	176(3)	C49–C48–C50–C53B	12(2)	C31–C30–C32–C34B	-11.5(16)	C67–C66–C68–C70A	-23(2)
C13–C12–C14–C16A	129.4(9)	C49–C48–C50–C51B	128(2)	C31–C30–C32–C33B	-127.7(14)	C67–C66–C68–C69B	-128.1(11)
C13–C12–C14–C17B	-69(3)	C49–C48–C50–C52A	-91.5(16)	C31–C30–C32–C35A	85.9(13)	C67–C66–C68–C71A	91(2)
C14–C12–C13–C8	-179.1(5)	C50–C48–C49–C44	179.4(6)	C32–C30–C31–C26	-179.0(6)	C68–C66–C67–C62	179.5(5)
O1–C1–C2–C3	-179.9(6)	O7–C37–C38–C39	-177.3(6)	O4–C19–C20–C21	178.0(6)	O10–C55–C56–C57	179.6(6)
O1–C1–C6–C5	-179.0(5)	O7–C37–C42–C41	-180.0(6)	O4–C19–C24–C23	179.9(5)	O10–C55–C60–C59	179.0(6)
O2–C7–C8–C13	166.7(6)	O8–C43–C44–C49	166.2(6)	O5–C25–C26–C31	-166.1(7)	O11–C61–C62–C67	-168.4(6)
O2–C7–C8–C9	-9.0(10)	O8–C43–C44–C45	-9.6(10)	O5–C25–C26–C27	11.7(10)	O11–C61–C62–C63	8.9(9)
O3–C9–C10–C11	179.8(7)	O9–C45–C46–C47	178.9(8)	O6–C27–C28–C29	-178.8(8)	O12–C63–C64–C65	-179.0(7)

Single crystal XRD data for compound **4ma (CCDC no. 2348646)**

checkCIF/PLATON report

Structure factors have been supplied for datablock(s) bod-bd-23-88

THIS REPORT IS FOR GUIDANCE ONLY. IF USED AS PART OF A REVIEW PROCEDURE FOR PUBLICATION, IT SHOULD NOT REPLACE THE EXPERTISE OF AN EXPERIENCED CRYSTALLOGRAPHIC REFEREE.

No syntax errors found. CIF dictionary Interpreting this report

Datablock: bod-bd-23-88

Bond precision:	C-C = 0.0040 A	Wavelength=1.54184	
Cell:	a=14.6923(3)	b=13.0967(2)	c=7.5634(2)
	alpha=90	beta=102.745(2)	gamma=90
Temperature:	298 K		

	Calculated	Reported
Volume	1419.50(5)	1419.50(5)
Space group	P 21/c	P 1 21/c 1
Hall group	-P 2ybc	-P 2ybc
Moiety formula	C19 H14 O2	C19 H14 O2
Sum formula	C19 H14 O2	C19 H14 O2
Mr	274.30	274.30
Dx,g cm-3	1.283	1.284
Z	4	4
Mu (mm-1)	0.655	0.655
F000	576.0	576.0
F000'	577.70	
h,k,lmax	18,16,9	18,16,9
Nref	3005	2956
Tmin,Tmax	0.949,0.974	0.760,1.000
Tmin'	0.949	

Correction method= # Reported T Limits: Tmin=0.760 Tmax=1.000
AbsCorr = MULTI-SCAN

Data completeness= 0.984 Theta(max)= 77.188

R(reflections)= 0.0693(2195) wR2(reflections)= 0.2829(2956)

S = 1.226 Npar= 191

```
The following ALERTS were generated. Each ALERT has the format
       test-name_ALERT_alert-type_alert-level.
Click on the hyperlinks for more details of the test.
```

Alert level C

```
PLAT084_ALERT_3_C High wR2 Value (i.e. > 0.25) ...................      0.28 Report
```

Alert level G

```
PLAT007_ALERT_5_G Number of Unrefined Donor-H Atoms ..............         1 Report
              H2
PLAT072_ALERT_2_G SHELXL First  Parameter in WGHT  Unusually Large      0.16 Report
PLAT912_ALERT_4_G Missing # of FCF Reflections Above STh/L=  0.600        48 Note
PLAT969_ALERT_5_G The 'Henn et al.' R-Factor-gap value ...........      9.71 Note
              Predicted wR2: Based on SigI**2  2.91 or SHELX Weight 23.86
PLAT978_ALERT_2_G Number C-C Bonds with Positive Residual Density.         0 Info
PLAT992_ALERT_5_G Repd & Actual _reflns_number_gt Values Differ by         3 Check
```

```
   0 ALERT level A = Most likely a serious problem - resolve or explain
   0 ALERT level B = A potentially serious problem, consider carefully
   1 ALERT level C = Check. Ensure it is not caused by an omission or oversight
   6 ALERT level G = General information/check it is not something unexpected

   0 ALERT type 1 CIF construction/syntax error, inconsistent or missing data
   2 ALERT type 2 Indicator that the structure model may be wrong or deficient
   1 ALERT type 3 Indicator that the structure quality may be low
   1 ALERT type 4 Improvement, methodology, query or suggestion
   3 ALERT type 5 Informative message, check
```

It is advisable to attempt to resolve as many as possible of the alerts in all categories. Often the minor alerts point to easily fixed oversights, errors and omissions in your CIF or refinement strategy, so attention to these fine details can be worthwhile. In order to resolve some of the more serious problems it may be necessary to carry out additional measurements or structure refinements. However, the purpose of your study may justify the reported deviations and the more serious of these should normally be commented upon in the discussion or experimental section of a paper or in the "special_details" fields of the CIF. checkCIF was carefully designed to identify outliers and unusual parameters, but every test has its limitations and alerts that are not important in a particular case may appear. Conversely, the absence of alerts does not guarantee there are no aspects of the results needing attention. It is up to the individual to critically assess their own results and, if necessary, seek expert advice.

Publication of your CIF in IUCr journals

A basic structural check has been run on your CIF. These basic checks will be run on all CIFs submitted for publication in IUCr journals (*Acta Crystallographica*, *Journal of Applied Crystallography*, *Journal of Synchrotron Radiation*); however, if you intend to submit to *Acta Crystallographica Section C* or *E* or *IUCrData*, you should make sure that full publication checks are run on the final version of your CIF prior to submission.

Publication of your CIF in other journals

Please refer to the *Notes for Authors* of the relevant journal for any special instructions relating to CIF submission.

PLATON version of 06/01/2024; check.def file version of 05/01/2024

Datablock bod-bd-23-88 - ellipsoid plot

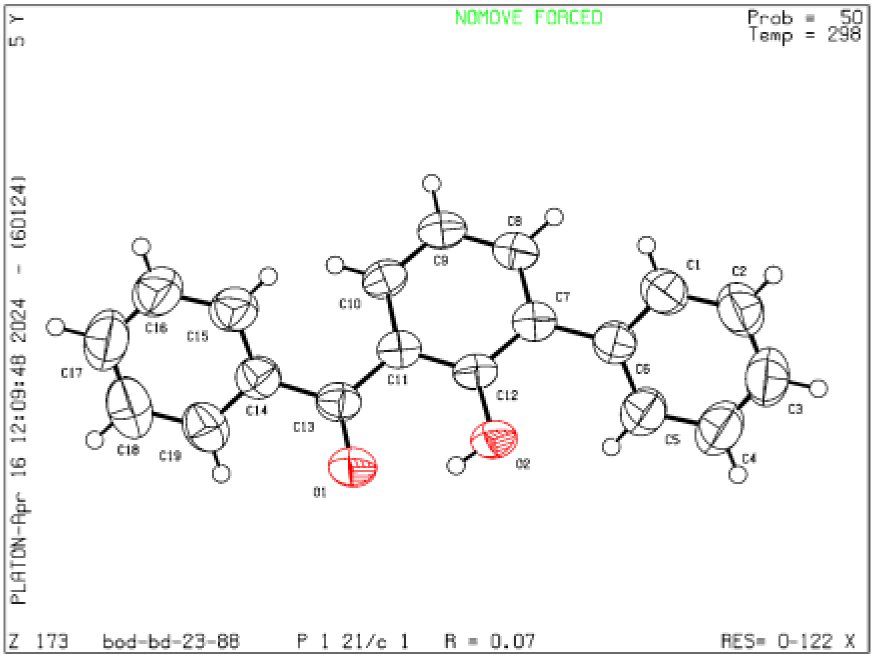

Table S9. Crystal data and structure refinement for **4ma (CCDC no. 2348646)**

Empirical formula	$C_{19}H_{14}O_2$
Formula weight	274.30
Temperature [K]	298
Crystal system	monoclinic
Space group (number)	$P2_1/c$ (14)
a [Å]	14.6923(3)
b [Å]	13.0967(2)
c [Å]	7.5634(2)
α [Å]	90
β [Å]	102.745(2)
γ [Å]	90
Volume [Å^3]	1419.50(5)
Z	4
ρ_{calc} [g/cm^3]	1.284
μ [mm^{-1}]	0.655
F(000)	576
Crystal size [mm^3]	0.08×0.08×0.04
Crystal colour	colourless
Crystal shape	plate
Radiation	Cu K_α (λ=1.54184 Å)
2Θ range [°]	6.17 to 154.38 (0.79 Å)
Index ranges	-18 ≤ h ≤ 18<br>-16 ≤ k ≤ 16<br>-9 ≤ l ≤ 8
Reflections collected	43973
Independent reflections	2956<br>R_{int} = 0.0683<br>R_{sigma} = 0.0237
Completeness to θ = 67.684°	100.0 %
Data / Restraints / Parameters	2956/0/191
Goodness-of-fit on F^2	1.226
Final R indexes [I≥2σ(I)]	R_1 = 0.0693<br>wR_2 = 0.2098
Final R indexes [all data]	R_1 = 0.1012<br>wR_2 = 0.2829
Largest peak/hole [eÅ^3]	0.26/-0.26

Table S10. Bond lengths and angles for **4ma (CCDC no. 2348646)**

Atom–AtomAtom–Atom–Atom	Length [Å]Angle [°]
O2–H2	0.8200
O2–C12	1.342(2)
O1–C13	1.229(3)
C1–C2	1.391(4)
C1–C6	1.391(4)
C2–C3	1.361(6)
C3–C4	1.369(5)
C4–C5	1.381(4)
C5–C6	1.391(4)
C6–C7	1.487(3)
C7–C8	1.388(3)
C7–C12	1.408(3)
C8–C9	1.380(3)
C9–C10	1.373(3)
C10–C11	1.399(3)
C11–C12	1.418(3)
C11–C13	1.474(3)
C13–C14	1.489(3)
C14–C15	1.394(4)
C14–C19	1.388(4)
C15–C16	1.378(4)
C16–C17	1.375(6)
C17–C18	1.374(6)
C18–C19	1.387(4)
C12–O2–H2	109.5
C2–C1–C6	120.5(3)
C3–C2–C1	120.3(3)
C2–C3–C4	120.0(3)
C3–C4–C5	120.6(3)
C4–C5–C6	120.6(3)
C1–C6–C5	118.0(2)
C1–C6–C7	119.9(2)
C5–C6–C7	122.0(2)
C8–C7–C6	120.4(2)
C8–C7–C12	118.0(2)
C12–C7–C6	121.7(2)
C9–C8–C7	122.4(2)
C10–C9–C8	119.4(2)
C9–C10–C11	121.1(2)
C10–C11–C12	118.6(2)
C10–C11–C13	122.2(2)
C12–C11–C13	119.12(19)
O2–C12–C7	117.4(2)
O2–C12–C11	122.27(19)
C7–C12–C11	120.26(19)
O1–C13–C11	121.0(2)
O1–C13–C14	118.2(2)
C11–C13–C14	120.8(2)
C15–C14–C13	123.0(2)
C19–C14–C13	117.5(2)
C19–C14–C15	119.4(3)
C16–C15–C14	119.7(3)
C17–C16–C15	120.4(3)
C18–C17–C16	120.5(3)
C17–C18–C19	119.7(3)
C18–C19–C14	120.2(3)

Table S11. Torsion angles for **4ma (CCDC no. 2348646)**

Atom–Atom–Atom–Atom	Torsion Angle [°]
O1–C13–C14–C15	133.9(3)
O1–C13–C14–C19	-42.6(3)
C1–C2–C3–C4	-0.5(6)
C1–C6–C7–C8	45.0(3)
C1–C6–C7–C12	-135.8(2)
C2–C1–C6–C5	0.6(4)
C2–C1–C6–C7	-177.4(3)
C2–C3–C4–C5	0.5(6)
C3–C4–C5–C6	0.0(5)
C4–C5–C6–C1	-0.5(4)
C4–C5–C6–C7	177.4(3)
C5–C6–C7–C8	-132.9(3)
C5–C6–C7–C12	46.3(3)
C6–C1–C2–C3	-0.1(5)
C6–C7–C8–C9	177.3(2)
C6–C7–C12–O2	0.9(3)
C6–C7–C12–C11	179.11(18)
C7–C8–C9–C10	2.6(3)
C8–C7–C12–O2	-179.89(19)
C8–C7–C12–C11	-1.7(3)
C8–C9–C10–C11	0.4(3)
C9–C10–C11–C12	-3.9(3)
C9–C10–C11–C13	178.2(2)
C10–C11–C12–O2	-177.39(19)
C10–C11–C12–C7	4.5(3)
C10–C11–C13–O1	170.1(2)
C10–C11–C13–C14	-9.6(3)
C11–C13–C14–C15	-46.4(3)
C11–C13–C14–C19	137.1(2)
C12–C7–C8–C9	-1.9(3)
C12–C11–C13–O1	-7.9(3)
C12–C11–C13–C14	172.46(18)
C13–C11–C12–O2	0.6(3)
C13–C11–C12–C7	-177.44(18)
C13–C14–C15–C16	-178.0(3)
C13–C14–C19–C18	179.9(3)
C14–C15–C16–C17	-0.8(5)
C15–C14–C19–C18	3.2(4)
C15–C16–C17–C18	1.4(6)
C16–C17–C18–C19	0.2(6)
C17–C18–C19–C14	-2.6(5)
C19–C14–C15–C16	-1.5(4)

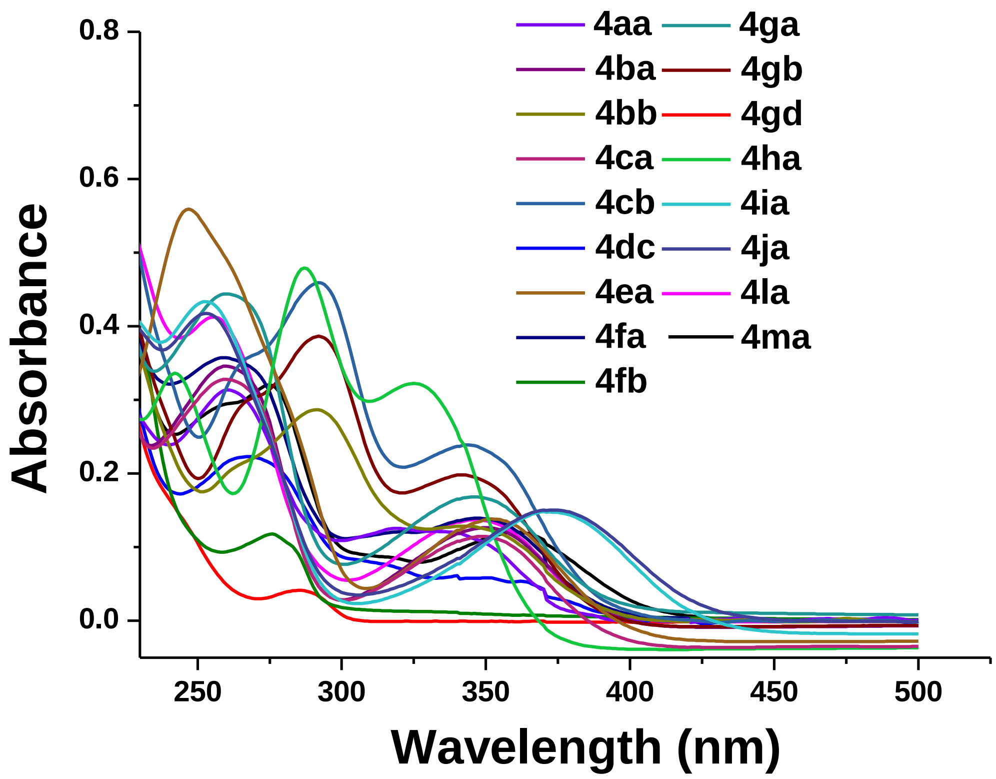

Figure S30. UV–vis absorption spectra of synthesized compounds **4aa–ma** from 225–500 nm.

Table S12. Optical properties of the compounds **4aa–ma**.

No.	λ_{max} (nm)	ε (mol^{-1} cm^{-1}L)	λ_C (nm)	Broad spectrum	UVA/UVB	SPF
**4aa**	261<br>334	7856<br>3051	387	Y	0.91	10.45
**4ba**	259<br>350	8663<br>3168	374	Y	1.73	7.69
**4bb**	292<br>352	7188<br>3140	386	Y	0.76	9.59
**4ca**	260<br>350	8205<br>2858	384	Y	1.11	7.60
**4cb**	291<br>345	11495<br>6050	380	Y	0.64	10.55
**4dc**	269	5615	387	Y	0.88	7.76
**4ea**	264<br>352	8908<br>3463	388	Y	0.95	6.66
**4eb**	292<br>243	10190<br>5233	365	Y	1.17	10.79
**4fa**	261<br>347	11063<br>4180	373	Y	1.25	7.70
**4fb**	292<br>342	9650<br>4940	364	Y	0.42	11.02
**4fd**	286	1050	343	N	0.08	1.97
**4ga**	287<br>326	11968<br>8033	350	Y	0.38	8.95
**4ha**	253<br>372	10828<br>3790	390	Y	2.44	4.90
**4ia**	253<br>373	10450<br>3808	390	Y	1.47	3.78
**4ja**	256<br>347	10298<br>3433	374	Y	1.24	7.37
**4ka**	275<br>362	7998<br>3035	386	Y	0.95	4.32
**4ma**	275<br>362	8077<br>3000	385	Y	1.07	8.07

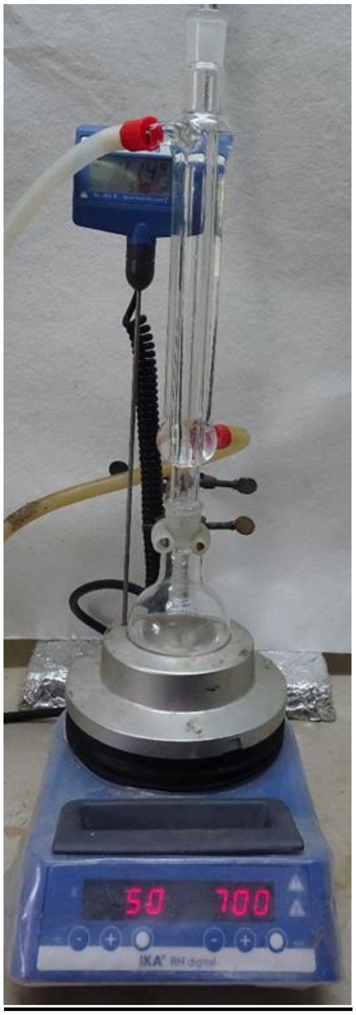

Figure S31. Picture of the reaction set-up for the synthesis of **4aa**-**4ma**.

References

1. Dhotare, B. B.; Kumar, M.; Nayak, S. K. *J. Org. Chem.* **2018**, *83*, 10089-10096.
2. Tang, Z.; Tong, Z.; Xu, Z.; Au, C. T.; Qiu, R.; Yin, S. F. Green Chem. 2019, 21, 2015-2022.
3. Tong, Z.; Tang, Z.; Au, C. T.; Qiu, R. *J. Org. Chem.* **2020**, *85*, 8533-8543.
4. Khanum, S. A.; Shashikanth, S.; Deepak, A. V. Bioorg. Chem. 2004, 32, 211-222.
